# Phenotypic and Genotypic Variation of Cultivated *Panax quinquefolius*

**DOI:** 10.3390/plants13020300

**Published:** 2024-01-19

**Authors:** Abdurraouf Abaya, Geovanna Cristina Zaro, Alvaro De la Mora Pena, Tom Hsiang, Paul H. Goodwin

**Affiliations:** School of Environmental Sciences, University of Guelph, Guelph, ON N1G 2W1, Canada; abayaa@uoguelph.ca (A.A.); gzaro@uoguelph.ca (G.C.Z.); delamora@uoguelph.ca (A.D.l.M.P.); thsiang@uoguelph.ca (T.H.)

**Keywords:** American ginseng, commercial production, phenotypic diversity, genotypic diversity, single nucleotide polymorphisms (SNPs), prolyl-tRNA synthetase, plant traits

## Abstract

American ginseng *(Panax quinquefolius*) is widely used due to its medicinal properties. Ontario is a major producer of cultivated American ginseng, where seeds were originally collected from the wild without any subsequent scientific selection, and thus the crop is potentially very diverse. A collection of 162 American ginseng plants was harvested from a small area in a commercial garden and phenotyped for morphological traits, such as root grade, stem length, and fresh and dry weights of roots, leaves, stems, and seeds. All of the traits showed a range of values, and correlations were observed between root and stem weights, root dry weight and leaf dry weight, as well as root and leaf fresh weights. The plants were also genotyped using single nucleotide polymorphisms (SNPs) at the PW16 locus. SNP analysis revealed 22 groups based on sequence relatedness with some groups showing no SNPs and others being more diverse. The SNP groups correlated with significant differences in some traits, such as stem length and leaf weight. This study provides insights into the genetic and phenotypic diversity of cultivated American ginseng grown under similar environmental conditions, and the relationship between different phenotypes, as well as genotype and phenotype, will aid in future selection programs to develop American ginseng cultivars with desirable agronomic traits.

## 1. Introduction

*Panax quinquefolius* L. (American ginseng) is cultivated commercially due to its roots containing medicinal substances, primarily ginsenosides, that have antioxidant, anti-inflammatory, antiapoptotic, and immunostimulatory activities, and it is widely used in the pharmaceutical, cosmetic, and food industries [1,2]. It is native to the hardwood forests of south-central Canada and eastern USA [3]; however, it is no longer legal to harvest from the wild in Canada after its inclusion in the Endangered Species Act in 2007 [4]. Ontario is a major producer of cultivated American ginseng with an annual value of approx. USD 250,000,000 from 2014 to 2017 [5]. Field cultivation relies on the use of seeds collected from previous crops, which are then transplanted into raised beds of fumigated soil [6,7]. Artificial shades are placed over the beds, and intensive cultivation practices like pesticides and fertilization are used until the harvesting of the roots in the third or fourth year of growth [8]. 

Seeds for field cultivation were originally collected randomly from wild populations in Ontario, and thus, the genotypes of American ginseng seed used for field cultivation are unknown, unlike certain Asian ginseng (*Panax ginseng*), where several elite cultivars are available [6,7]. However, it is possible that there are landraces of American ginseng that have developed from specific growers selecting seed stocks for particular traits, such as root shape and berry color. For instance, in Ontario, three landraces were identified based on ginsenoside and sucrose levels [9].

Phenotyping is a fundamental approach to characterizing the diversity and variation within plant populations. Phenotyping involves the observation and measurement of observable traits, including morphological, physiological, and biochemical characteristics [10]. While there are studies phenotyping wild American ginseng [11,12], the majority of the literature is just reports of average phenotypes for cultivated American ginseng [13,14]. However, in addition to the genetics of the plant, environmental conditions can greatly affect the phenotype of ginseng. For example, *P. ginseng* showed increased leaf area but relatively stable flowering time and petiole lengths when grown in shade and deep-shade compared to sunlight [15]. Another example is temperature, which affected the growth and differentiation of adventitious roots of *P. ginseng*, with lower temperatures resulting in greater differentiation but less growth [16]. Thus, environmental conditions should be highly similar when trying to compare phenotypes and genotypes.

Genotyping is also a fundamental approach to characterizing the diversity within plant populations, focusing on the assessment of genetic markers within an organism’s genome [10]. Genotyping with molecular markers can offer a precise and accurate assessment of genetic variation within the population [17]. A number of approaches have been used to genotype ginseng, including isozymes, restriction fragment length polymorphisms, PCR product length polymorphisms, and DNA sequence analysis to detect insertion/deletions or single nucleotide polymorphisms (SNPs) [6]. These methods have been used to compare elite cultivars of *P. ginseng*, and landraces of *P. quinquefolius*. SNPs based on a genomic library of an elite *P. ginseng* cultivar were able to distinguish between five cultivars [18]. Another example is the identification of SNPs from the Panax dbEST database at GenBank, where 160 SNPs from *P. quinquefolius* and 171 SNPs from *P. ginseng* were found [19]. The SNPs revealed a high level of nucleotide diversity, with one to nine haplotypes per locus for *P. quinquefolius* and one to twelve haplotypes per locus for *P. ginseng*. For *P. quinquefolius*, the locus with the most haplotypes (9), highest haplotype diversity (0.978), and highest nucleotide diversity for total sites (0.035) was PW16, which had a functional annotation of prolyl-tRNA synthetase.

The objective of this study was to phenotype and genotype a collection of cultivated *P. quinquefolius* plants in order to understand their genetic diversity and morphological variation within a population. To limit the impact of the environment on phenotype, plants of the same age were all collected at the same time within a small area of a single raised bed in a commercial ginseng garden. Phenotyping was carried out by measuring a variety of a number of morphological traits, such as root grade based on shape [20], and genotyping was carried out using SNPs at the PW16 locus. The findings of this study will contribute to knowledge of the morphological and genetic variation of cultivated *P. quinquefolius* grown under almost the same environmental conditions. This information will aid in the knowledge needed to design approaches for future work on the selection and breeding of improved *P. quinquefolius* cultivars with desirable agronomic traits.

## 2. Results

### 2.1. Morphological Ginseng Parameters

In the central part of a cultivated bed, 162 plants were gathered from a 4 m^2^ region, resulting in an average plant density of 40.5 per m^2^. Among these plants, the root grades were pencil for 79 plants, fork for 34 plants, spider for 31 plants, and chunk for 18 plants. All the plants possessed one stem per root, except for two plants that had two stems per root. Sampling was carried out during the period of seed ripening, and all the plants, except for 10, had seed heads.

**Figure 1:**
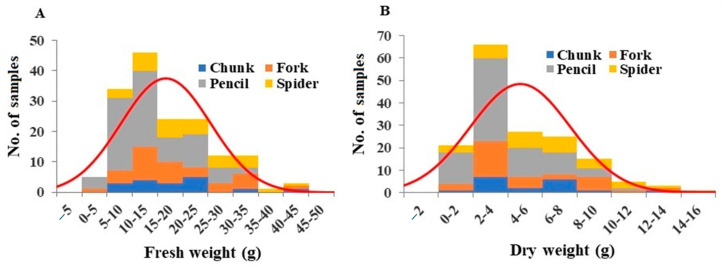
Distribution of root fresh weight (**A**) and dry weight (**B**) among the plant samples. Division of samples by root grade is also shown.

The fresh weight of roots per plant varied between 3.58 and 42.68 g, with an average of 16.66 g (Figure 1A). The fresh weight of pencil roots were significantly lower compared to the other grades (Table S1). Root dry weights ranged from 0.30 g to 14.07 g, with an average of 4.74 g (Figure 1B). Similar to the fresh weights, pencil roots showed significantly lower dry weights compared to the other grades (Table S1). A comparison of fresh and dry root weights per plant showed that the average root water content was 27.76%, with chunk roots having the highest and forked roots having the lowest water content (Table S1). 

**Figure 2:**
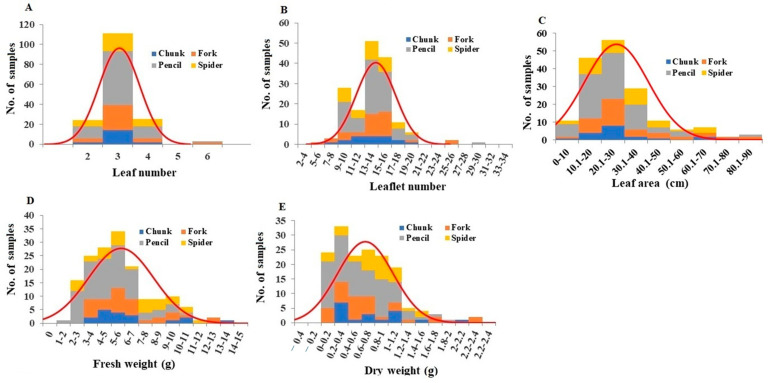
Distribution of leaf number (**A**), leaflet number (**B**), leaf area (**C**), leaves fresh weight (**D**), and leaves dry weight (**E**) among the plant samples. Division of samples by root grade is also shown.

The number of leaves per plant ranged from 2 to 6, with a mean leaf number per plant of 3.00 (Figure 2A). The mean leaf numbers for different root grade plants were not significantly different (Table S2). The number of leaflets per plant ranged from 6 to 29, with a mean leaflet number per plant of 13.65 (Figure 2B). Leaflet number was significantly higher for pencil root grade plants than all of the other grades, which were not significantly different from each other (Table S2). A comparison of leaflet and leaf number per plant showed that the average was 4.49 leaflets per leaf. There were 2 plants with an average of six leaflets per leaf, 101 plants with five leaflets per leaf, 48 plants with four leaflets per leaf, 10 plants with three leaflets per leaf, and 1 plant with two leaflets per leaf. The leaf area per plant ranged from 5.03 cm to 76.03 cm, with an average of 26.45 cm (Figure 2C). The leaf area of pencil root grade plants was significantly lower than the leaf areas of plants in all other root grades (Table S2).

The fresh weights of leaves per plant ranged from 1.39 to 13.50 g, with an average of 5.71 g (Figure 2D). Leaves from pencil root grade plants had significantly lower fresh weights compared to the leaves of other root grades (Table S2). Leaf dry weight ranged from 0.016 g to 2.285 g, with a mean of 0.667 g (Figure 2E). Leaf dry weight from pencil root grade plants was significantly lower compared only to leaves from spider root grade plants (Table S2). Based on fresh and dry leaf weights per plant, the average leaf water content was 11.62%, with the highest for pencil root grade plants and the lowest for chunk root grade plants (Table S2).

**Figure 3:**
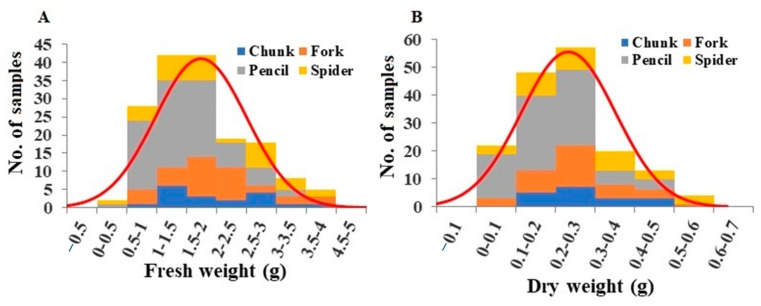
Distribution of leaf petiole fresh weight (**A**) and leaf petiole dry weight (**B**) among the plant samples. Division of samples by root grade is also shown.

For leaf petioles, fresh weights per plant ranged from 0.39 to 3.85 g, with a mean of 1.70 g (Figure 3A). Leaf petioles from pencil root grade plants had significantly lower fresh weights compared to the other grades (Table S3). Leaf petiole dry weights ranged from 0.044 g to 0.581 g, with a mean of 0.22 g (Figure 3B). Leaf petioles from pencil root grade plants had significantly lower dry weights than other grades (Table S3). Based on fresh and dry petiole weights per plant, the average leaf petiole water content was 13.35%, with spider and fork root grade plants having the highest and lowest petiole water contents, respectively (Table S3).

**Figure 4:**
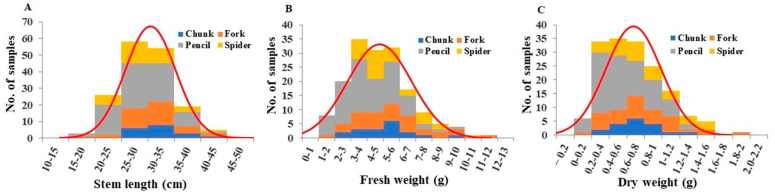
Distribution of stem length (**A**), stem fresh weight (**B**), and stem dry weight (**C**) among the plant samples. Division of samples by root grade is also shown.

Stem length per plant ranged from 16.5 to 42.0 cm, with an average of 30.06 cm (Figure 4A). Pencil root grade plants showed significantly shorter stem lengths compared to chunk root grade plants (Table S3). Stem fresh weights ranged from 1.18 to 11.04 g, with a mean of 4.76 g (Figure 4B). Pencil root grade plants had significantly lower fresh weights compared to fork and spider root grade plants (Table S3). Stem dry weights ranged from 0.03 g to 1.82 g, with a mean of 0.67 g (Figure 4C). Pencil root grade plants had significantly lower dry weights compared to fork and spider root grade plants (Table S3). Based on fresh and dry stem weights per plant, the average stem water content was 14.35%, with spider and pencil root grade plants having the highest and lowest stem water contents, respectively (Table S3).

**Figure 5:**
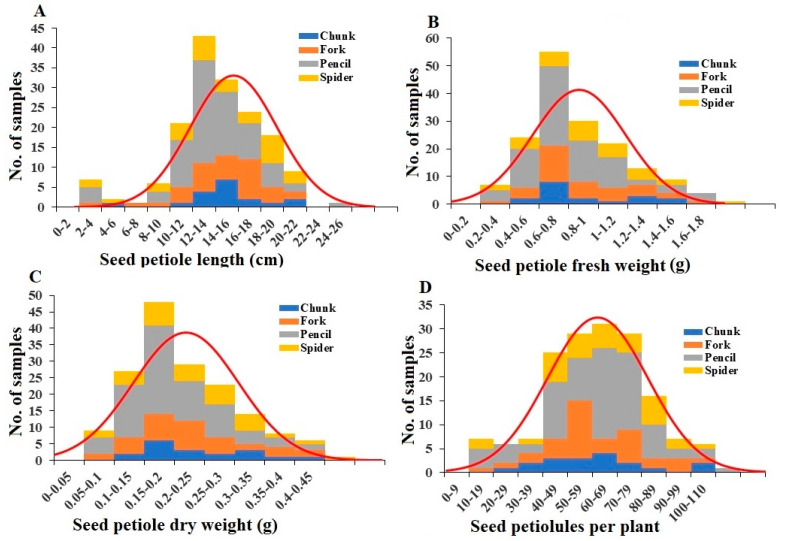
Distribution of seed petiole length (**A**), seed petiole fresh weight (**B**), seed petiole dry weight (**C**), and seed petiolule number per plant (**D**) among the plant samples. Division of samples by root grade is also shown.

Among the 162 plants, 7 did not have a seed petiole or seed cluster. All the other plants had one seed petiole with multiple seed petiolules arising from it with either no or one seed at the end of each petiolule, forming a single seed cluster per plant. The length of seed petioles varied between 0.5 and 22 cm, with an average of 10.45 cm (Figure 5A). Only one plant had an unusually short seed petiole of 0.5 cm. The mean lengths for the different root grade plants were not significantly different (Table S4). Fresh weights of seed petiole per plant ranged from 0.03 to 1.5 g, with an average of 0.47 g (Figure 5B). The mean fresh weights for pencil root grade plants were significantly less than those of spider grade plants (Table S4). Seed petiole dry weights ranged from 0.01 g to 0.39 g, with a mean of 0.12 g (Figure 5C). Similar to seed petiole fresh weights, pencil root grade petioles had a significantly lower dry weight compared to spider, fork, and chunk root grade plants (Table S4). A comparison of fresh and dry seed petiole weights showed that the average water content in the seed petioles was 26.05%, with chunk and pencil having the highest and lowest water contents, respectively (Table S4). For the 156 plants with seed petioles, the number of seed petiolules per plant ranged from 5 to 92, with an average of 45.28 (Figure 5D). The mean numbers of seed petiolules for the different root grade plants were not significantly different (Table S4). 

**Figure 6:**
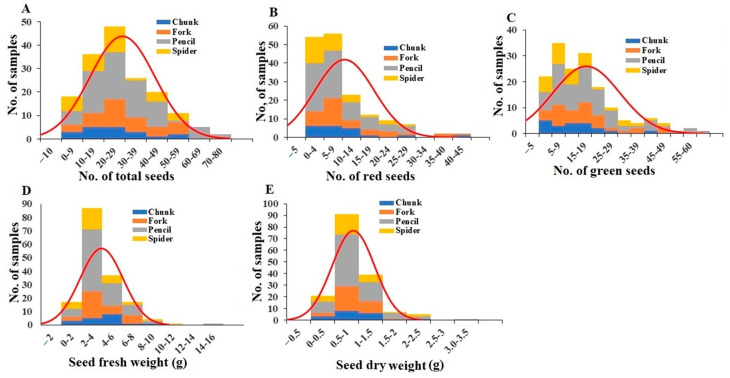
Distribution of total seed number (**A**), red seed number (**B**), green seed number (**C**), seed fresh weight (**D**), and seed dry weight (**E**) among the plant samples. Division of samples by root grade is also shown.

The total number of seeds per plant ranged from 0 to 65, with an average of 18.76 (Figure 6A). The number of red seeds per plant varied from 0 to 39, with an average of 5.69 (Figure 6B), and the number of green seeds per plant ranged from 0 to 58, with a mean of 13.07 (Figure 6C). A comparison of the average total seed number per plant to average seed petiolule number per plant indicates that 42.98% of the seed petiolules per plant had a seed attached. There were no significant differences in total, red, or green seeds per plant between the different root grade plants (Table S4). Total seed fresh weight per plant ranged from 0.01 g to 13.64 g, with a mean of 1.94 g (Figure 6D). There were no significant differences in fresh total seed weight per plant between the different root grade plants (Table S4). For total seed dry weight per plant, values ranged from 0.03 g to 2.77 g, with a mean of 0.41 g (Figure 6E). There were no significant differences in total dry seed weight per plant between the different root grade plants (Table S4). On average, the total seed water content was 21.05%, with the highest and lowest water contents for chunk and forked grade plants, respectively (Table S4).

### 2.2. Phenotypic Correlations

**Figure 7:**
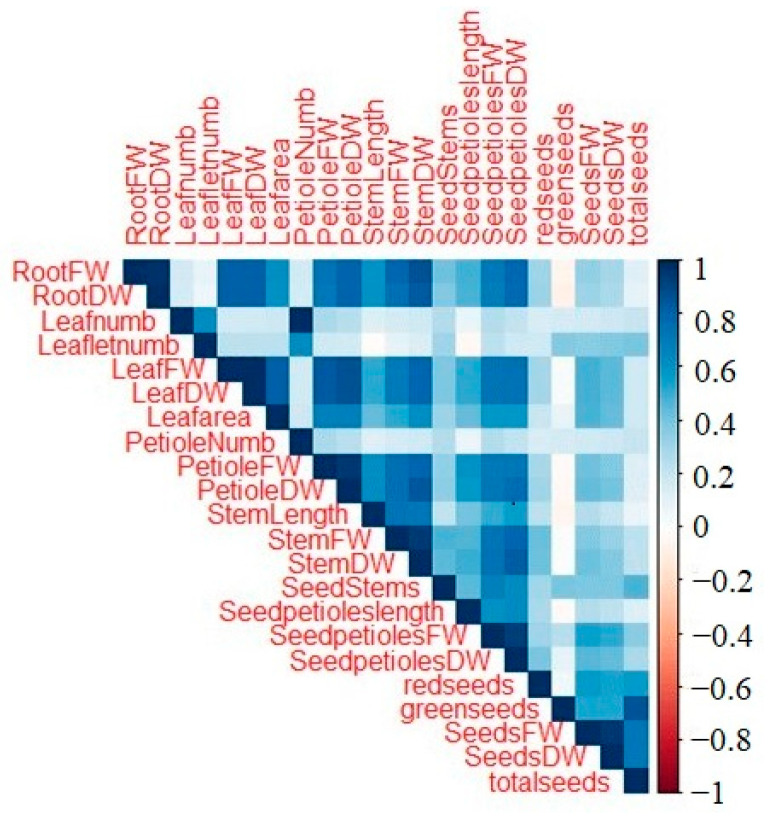
Correlations between different phenotypic parameters of the samples.

A comparison between all the phenotypic parameters measured showed that there were significant correlations between many of the parameters, all of which were positive (Figure 7). Among those, root fresh weight was significantly correlated with leaf and stem fresh weight as well as seed petiole dry weight (Figure S1). Root dry weight was significantly correlated with leaf, petiole, stem, and seed petiole dry weight (Figure S2). Leaf fresh weight was significantly correlated with leaf area and petiole fresh weight (Figure S3). Leaf dry weight was significantly correlated with leaf area, petiole, stem, and seed petiole dry weight (Figure S4). Leaf petiole dry weight was significantly correlated with stem and petiole dry weight (Figure S5). Stem length was significantly correlated with stem fresh and dry weight (Figure S6). Stem dry weight was significantly correlated with seed petiole dry weight (Figure S7).

### 2.3. Genetic Relatedness of the P. quinquefolius Samples

For the 740 bp region amplified with the PW16 primers, SNPs were observed at 52 sites among the 162 samples that were distributed throughout the sequence, whereas 586 sites had no SNPs (Figure S8). No indels were observed. There were 147 sequence variants in the population, with the most common having identical sequences for 15 samples. A tree of the PW16 sequences showed that the samples could be clustered into 22 groups, designated PW16-1 to PW16-22 (Figure S9). Sequences within most PW16 groups were identical or relatively highly similar, indicating that these samples were relatively closely related to each other, perhaps reflecting that the plants near each other may have come from closely related seeds. However, PW16 groups 3, 11, and 22 had relatively high diversity. The samples in those clusters were notable for having a relatively high number of sites with some of the least common SNPs.

### 2.4. Genetic Relatedness Compared to Phenotypic Parameters

The number of samples in each group varied from 2 to 19 (Table 1). All the groups had some samples with pencil and forked grade roots, except for group 12 lacking pencil and group 20 lacking forked root samples. Chunk and spider grade root samples were less common, with six groups having no chunk and eight groups having no spider grade root samples. Pencil roots were found in 50% or more of the samples in groups 7, 9, 10, 15, 16, 19, 20, and 21, whereas chunk roots were found in 50% or more of the samples in groups 6 and 12, forked roots were found in 50% or more of the samples in groups 12 and 15, and spider roots were only found in 50% or more of the samples in group 3. 

Although root fresh and dry weights ranged widely between the PW16 groups, there were no significant differences between the groups (Table S5). Both average leaf fresh and dry weights of group 7 samples were significantly higher than those of group 22 (Table 2). There were no significant differences between groups for average number of leaves per plant (Table S5). Group 11 samples had a significantly average higher number of leaflets per plant than those of group 3, and the average number of leaflets per leaf in samples of groups 1, 6, 7, 10, 12, 16, 18, 19, and 20 were significantly higher than those of group 3 (Table 2). Average leaf petiole fresh weight of group 5 samples was significantly higher than those of group 19 (Table 3). For stem lengths, those of group 7, 11, and 12 samples were significantly longer than those of group 19 (Table 3). Average stem fresh weight of group 7 samples was significantly higher than those of groups 6, 8, 9, 10, 13, 14, 15, 16, 18, 19, 21, and 22, and average stem dry weight of group 7 was significantly higher than those of groups 6, 8, 10, 13, 14, 15, 16, 19, 20, and 22 (Table 3). 

For average seed petiole length, groups 1, 5, 6, 7, 11, 15, 16, and 22 samples were significantly longer than those of group 20 (Table 4). Average seed petiole fresh weight for groups 1, 5, and 7 samples were significantly higher than those of groups 20 and 22, and average seed petiole dry weight of group 7 was significantly higher than those of groups 8, 12, 14, 16, 20, and 22 samples. For seed petiolule number per plant, groups 1, 4, 7, 11, 15, 17, 18, and 19 samples had a significantly average higher number than those of groups 12 and 20 (Table 4). The average number of red seeds per plant of group 4 was significantly greater than those of group 6, 10, and 15 samples, while green seeds per plant of group 15 were significantly greater than those of groups 1, 6, 8, 10, 11, 12, 14, 17, 19, 20, 21, and 22 samples (Table 5). Average number of total seeds per plant for groups 3, 4, 15, and 18 were significantly higher than those of groups 10, 19, and 22 samples. Average total seed fresh weight of group 3 was significantly higher than those of groups 1, 10, 19, 20, and 22, and average total seed dry weight of group 3 was significantly higher than those of groups 10, 19, and 20 (Table 5).

Overall, most parameters showed some significant differences between groups, except for the root biomass, which had considerable ranges, but variation was such that there were no significant differences. Group 7 was notable for having one of the highest values for root fresh and dry weights, leaf fresh and dry weights, stem length, stem fresh weight, stem dry weight, and seed petiole fresh and dry weights. While group 11 also had notably high root fresh and dry weights, it was only also notably high in the number of leaflets per plant. Group 22 was notable for having one of the lowest values for root fresh and dry weight, leaf fresh and dry weight, seed petiole fresh and dry weight, green seeds per plant, and total seeds per plant.

## 3. Discussion 

SNPs are a valuable tool in plant genotyping because they are abundant in the genome and can serve as genetic markers for various purposes, such as identifying specific genes related to traits of interest or tracking genetic diversity within a population [21,22,23]. For ginseng, SNPs have primarily been used to study the genetic uniformity of elite cultivars, founder effects in different populations, the invasion of wild populations by cultivated ones, and comparisons of the diversity of different populations [6]. An important advantage of using SNPs in genotyping is their abundance throughout the genome, making them excellent markers for studying genetic variation within populations [24]. In this study, the PW16 locus was selected based on its previous demonstration to have a relatively high SNP density among *P. quinquefolius* samples [19]. The proposed function of the PW16 locus is prolyl-tRNA synthetase, also known as proline tRNA ligase. This enzyme is part of the aminoacyl-tRNA synthetase family and plays a crucial role in protein synthesis by catalyzing the attachment of proline to its corresponding transfer RNA molecule [25]. In plants, it has specific functions related to translation and protein synthesis [26]. As an essential gene for plants, it would have many indirect effects on metabolism, but it is unlikely to be the primary major factor affecting any of the phenotypic traits measured in this study. Thus, it is being used as a marker for genetic diversity rather examining the function of prolyl-tRNA synthetase in plant growth and development.

Phenotyping of the plants in this study was carried out on a wide range of traits of roots, stems, leaves, and seeds of cultivated *P. quinquefolius* plants. In Ontario, cultivated American ginseng plants have been described as being typically 45–60 cm in height with 3-year-old plants typically having three leaves with three to five leaflets per leaf [13]. The stems of the 3-year-old plants in this study were on average of 30 cm long, which would be less than typical, with the longest stem only reaching 42 cm. However, almost 70% of the plants had 3 leaves with an average of 4.5 leaflets per leaf, typical of that of 3-year-old plants. For three large natural populations of *P. quinquefolius*, leaf area was positively, but not linearly, related to plant age, with most 3-year-old plants being approx. 50 cm^2^ [27]. This was higher than the average in this study of 27.7 cm^2^, corresponding to the shorter plant height, possibly due to less favorable conditions in a cultivated field where plant density is higher. The fresh weight of 3-year-old roots should be 15–20 g, but can be higher under more favorable conditions [13]. In this study, the average root fresh weight was 16.7 g, indicating that conditions for root growth were typical for cultivation, despite the plants being shorter with less leaf area than typical. For 2-year-old plants, flower heads are reported to occur on 20–50% of plants, which increases in older plants [13]. This study showed that flower heads were on 89% of 3-year-old plants, which is likely related to being 3- rather than 2-year-old plants. In general, the 3-year-old plants in this study agree well with the predicted traits, except for shorter stem lengths and lower leaf area. While typical phenotypes of cultivated American ginseng plants of a number of traits measured in this study have been reported, no previous reports quantifying the range of these traits for plants under similar environmental conditions were found. This study indicates that there can be considerable diversity in these traits under cultivation within a population, possibly due to environmental factors, genetic variability, or other influences that can impact the phenotypic characteristics of cultivated *P. quinquefolius* plants. 

The classification of the root grades was developed for 3- and 4-year-old plants based on market preferences and was designed to be applicable to nearly all American ginseng roots [20]. However, it is not clear if there was a genetic basis for a classification based on root shape. For example, Roy et al. [20] found that, except for the fiber root grade, which generally are small secondary and tertiary roots of other root grades, root shape grades did not significantly differ in any root ginsenoside or ginsenoside-like compounds. However, root shape may have a genetic basis as certain cultivars of *P. ginseng* are noted for their characteristic root shapes [28]. On the other hand, seeding depth affected the length and width of *P. quinquefoilus* roots, indicating that environmental factors can affect root shape [29]. In this study, all the taproot shape grades of Roy et al. [20] were observed, but the pencil root grade predominated with 49% of the roots. The cases in this study with significant differences in phenotypic traits based on root grade were lower fresh and dry weights of roots and lower petiole fresh and dry weights, as well as shorter stem lengths of pencil root grade plants than any other root grade plants. However, pencil root grade plants had significantly higher leaflet numbers and total seeds per plant than all the other root grade plants. These significant differences indicate that pencil root grade plants tend to be the most distinct, with shorter plants and less biomass, although some traits were higher. 

In this study, significant positive correlations between root fresh or dry weights versus stem fresh or dry weights and root fresh or dry weights versus leaf fresh or dry weights were found likely because larger roots could store more nutrients for greater stem development in spring, and then greater leaf mass could allow for more nutrients stored in the roots for larger roots. Greater leaf mass for more photosynthesis and thus greater growth could explain the significant positive correlations between leaf dry weight versus leaf area, petiole dry weight, stem dry weight, and seed petiole dry weight. Another positive correlation between traits were leaf fresh weight versus leaf area and petiole fresh weight, all indicating larger leaves with larger petioles. Larger plants with more biomass in multiple plant parts also could explain why leaf petiole dry weight was positively correlated with stem dry weight and seed petiole dry weight, stem length was positively correlated with stem fresh and dry weight, and stem dry weight was positively correlated with seed petiole dry weight. These positive correlations may be at least partially related to the genetics of the plants, considering their similar age and growth environments. In contrast, there were no negative correlations were between traits, suggesting that number, size, or biomass of one plant part did not result in a corresponding decrease in another plant part.

Other studies quantifying the phenotypic variation of *P. quinquefoilus* have been conducted only for wild populations. However, as there appears to have been considerable planting of seed from commercial gardens into forests for harvesting as “wild” ginseng, it may not be simple to distinguish wild from cultivated genotypes from the location [30]. Mooney and McGraw [11] examined 12 sites from Indiana, Maryland, New York, Pennsylvania, Virginia, and West Virginia, with plants ranging from seedlings to over 20 years of age. Plant age was a predictor of leaf area, stem height, and fruit set. Plants in areas with high harvest pressure exhibited smaller leaf areas, shorter stems, and fruit set, which appeared to be heritable traits, as evidenced by their persistence after transplantation to a common environment. Carpenter et al. [12] reported that plants in 20 southern Wisconsin wild ginseng sites had considerable diversity with age based on leaf scar, ranging from 1 to 24 years. All the correlations for these plants of different ages between leaf number, leaflet number, flower bud number, and leaf area of the largest leaflet were significant. However, these results are difficult to relate to this study as different-aged plants were compared, and age appeared to be the major source of the differences, unlike in this study where all the plants had been planted at the same time. Also, there would be environmental differences between and within these wild wooded sites that could affect the phenotype. 

Several other studies of the genetic variation of cultivated *P. quinquefoilus* have been conducted, with all using only RAPD markers. Bai et al. [31] showed a high level of genetic variation in a cultivated population from Ontario, with each of the 48 plants possible to distinguish with the RAPD markers. This diversity was supported by the observation of a high diversity in plant height, leaf number, flowering, berry maturation, and other phenotypic traits, although the data for those traits was not shown. For plants selected based on greater plant height, lower genetic diversity was detected than randomly selected plants, indicating that genetic factors contributed to morphological variation. Also, unpublished RAPD data by Bai et al. [31] with pollen from individual plants confirmed that American ginseng is self-pollinating. Schluter and Punja [32] examined 127 cultivated *P. quinquefolius* samples from eight fields in British Columbia, Ontario, and Nova Scotia and found a genetic distance within those populations comparable to wild populations. For cultivated plants, the greatest diversity was from Ontario, followed by British Columbia, Wisconsin, and then Nova Scotia samples, with no clustering by geographic location for the Canadian samples but some by garden within a location. One round of selection for plants with large roots did not reduce the level of diversity compared to plants without selection. Lim et al. [33] showed that one cultivated population from Wisconsin had similar genetic dissimilarity to some of the wild populations from New York, Kentucky, Tennessee, North Carolina, Pennsylvania, and Virginia. Gel images of the RAPD patterns showed that each of the three samples from the Wisconsin cultivated population samples were distinct. Obae and West [34] found that the genetic variations of cultivated and wild plants in West Virginia were similar, and were also similar to cultivated plants in Wisconsin and Pennsylvania. The similarity between wild and cultivated populations was believed to be due to growers collecting seed from the wild. Variation mostly occurred within populations in the cultivated plants, whereas variation was mostly between populations for wild plants, likely due to sharing of seed between growers. Schlag and McIntosh [35] revealed that populations from cultivated Maryland landraces were more diverse than cultivated and commercial seed sources from Tennessee and Wisconsin. While some results differ, these studies indicate that plants under cultivation generally do not have reduced genetic diversity from those in wild populations, and cultivated populations can have more variation within a garden, which can be related to phenotype. While each plant could often be distinguished by a unique set of RAPD bands, self-pollination can result in plants that are very similar. This study has shown that genotyping using SNPs can also show considerable variation and cluster samples, with some groups ranging from relatively high to no variability at the PW16 locus, with low variability perhaps related to self-pollination. 

While this study provides valuable insights into the genetic and phenotypic variation of cultivated *P. quinquefolius*, there are certain limitations to consider. While a sample size of 162 plants is considerable, expanding the study to include a larger number of plants would provide a more comprehensive understanding of the phenotypic and genetic diversity. In addition, only the PW16 locus was examined, but future work could examine SNPs in other genes, which may reveal additional genetic differences. Also, while the plants were all chosen adjacent to each other under highly similar conditions typical of commercial production, phenotyping plants under different light intensities, soil fertilities, and temperatures could reveal the impact of those factors on the diversity of phenotypes, offering a different perspective on the interactions between genetics and the environment. 

## 4. Materials and Methods 

### 4.1. Plant Material and Measurements

The *P. quinquefolius* plants in this study were harvested within a 4 m^2^ area in the center of a shaded 3-year-old commercial ginseng garden near Simcoe, Ontario, on 20 August 2019. The resulting collection of American ginseng samples were composed of entire plants with roots, leaves, petioles, stems, and seeds with seed petioles. In the laboratory, the roots were washed with tap water to remove all visible soil particles. Roots were characterized by grade into spider, chunk, forked, or pencil [20]. Root color was determined for all roots based on “The Official Visual Aids (USDA)”, changing the names to numbers for quantitative analysis (light = 1, light medium = 2, medium = 3, dark medium = 4, dark = 5) [36]. Root fresh weight was determined after washing the roots and removing excess moisture with paper towels. Root dry weight was determined after drying the roots at 60 °C for 72 h. The number of petioles, leaves, and leaflets per plant were recorded, along with their fresh and dry weights. Leaf area was determined with a LI-COR LI-3100C Area Meter (LI-COR Inc., Lincoln, NE, USA). Stem length per plant and the fresh and dry weights per plant were also measured. The number of green and red seed, seed petioles and seed petiole length per plant were counted, and their fresh and dry weights measured. 

### 4.2. Phylogeny 

Root samples from individual plants were stored at −80 °C. DNA was extracted as per Edwards et al. [37]. The extracted DNA was stored at −20 °C. PCR was conducted in a final volume of 20 μL, comprising 1 μL of DNA extract (1 ng/μL), 2.97 μL of each 25 μM primer, 7.13 μL PCR water, 2.97 μL PCR buffer, 1.78 μL of 15 mM Mg^++^, 0.59 μL of 1.25 mM dNTP, and 0.59 units of Tsg polymerase (Biobasic, Markham, ON, Canada) with 10× Tsg polymerase buffer. The forward primer PW16F (5′-AAAGAAGCTGTTTGTTTCAGAGCTGG-3′) [19] was paired with newly designed reverse primer PW16R (5′-TCTGGAGTGGCATGAGCAGTATGT-3′), which was designed from an alignment of *P. quinquefolius* prolyl-tRNA synthetase sequences at NCBI found using the sequences most closely matching that of the PW16 primers of Li et al. [19]. The PW16F and PW16R primers were predicted to amplify a 740 bp region. The PCR parameters consisted of 94 °C for 3 min, followed by 25 cycles of 30 s at 94 °C, 60 s at 62 °C, and 60 s at 72 °C, and finally 72 °C for 10 min. The length of the PCR products was examined through electrophoresis in a 1.5% agarose gel in TBE buffer. Sequencing was carried out at the Agriculture & Food Laboratory at the University of Guelph. The sequence identity was checked via comparison to the GenBank nr database at NCBI using BLASTn (www.ncbi.nlm.nih.gov). Alignment of the sequences was carried out with MUSCLE (https://www.ebi.ac.uk/Tools/msa/muscle/, accessed 2 February 2022). The alignments were then used to generate a maximum-likelihood phylogram using MEGA-X software v10 [38]. The phylogram was constructed with 1000 bootstrap replications, and the percentage values were displayed on the branches.

### 4.3. Statistical Analysis

Histograms were created in Excel (Microsoft) v2208 to show the distribution of each factor measured per plant, with each measurement divided by root grade of each plant. All data for each phenotype traits were separated according to the PW16 genotype group and were subjected to analysis of variance (ANOVA) as implemented in the GLM procedure of SAS version 9.4 (SAS Institute, Cary, NC, USA). When significant differences (*p* < 0.05) were found, Fisher’s Least Significant Test (*p* = 0.05) was used to compare means. Linear regression analysis was conducted using R (version 4.3.1) with the “corrplot” and “Hmisc” packages to determine the relationship between different traits. 

## 5. Conclusions

In conclusion, an examination of the genetic diversity and phenotypic variation among a collection of cultivated *P. quinquefolius* plants showed that there was considerable genetic diversity, as assessed with SNPs at the PW16 locus, and phenotypic diversity, based on 22 morphological traits. The correlations between a number of morphological traits showed that they are related, even among plants of the same age, and may be related to genetic or physiological mechanisms for the coordination of growth and development. Clustering of the SNPs showed that some groups of samples had significant phenotypic differences from others, and thus markers could potentially be used for selection. Furthermore, the observed associations could have practical implications for selection, such as selecting plants with greater stem or leaf weights in order to select for greater root weights without having to extract roots from the soil. As the demand for American ginseng continues to grow, harnessing the genetic diversity and phenotypic potential of cultivated populations will become increasingly important.

## Figures and Tables

**Table 1:** The number of samples and percentage in each root grade of the samples belonging to the PW16 groups designated in Figure S9.

Group No.	No. of Samples	Pencil	Chunk	Forked	Spider
1	3	0.33%	33.33%	33.33%	0.00%
2	14	0.27%	20.00%	40.00%	13.33%
3	6	0.33%	0.00%	16.67%	50.00%
4	19	0.38%	12.50%	37.50%	12.50%
5	7	0.40%	20.00%	40.00%	0.00%
6	4	0.25%	50.00%	25.00%	0.00%
7	5	0.57%	0.00%	28.57%	14.29%
8	6	0.38%	12.50%	37.50%	12.50%
9	15	0.52%	13.04%	17.39%	17.39%
10	3	0.67%	0.00%	33.33%	0.00%
11	12	0.46%	15.38%	15.38%	23.08%
12	2	0.00%	50.00%	50.00%	0.00%
13	11	0.46%	15.38%	30.77%	7.69%
14	6	0.20%	20.00%	20.00%	40.00%
15	4	0.50%	0.00%	50.00%	0.00%
16	5	0.83%	0.00%	16.67%	0.00%
17	5	0.40%	0.00%	20.00%	40.00%
18	6	0.22%	11.11%	44.44%	22.22%
19	4	0.80%	0.00%	20.00%	0.00%
20	4	0.60%	20.00%	0.00%	20.00%
21	14	0.54%	7.69%	23.08%	15.38%
22	7	0.43%	14.29%	28.57%	14.29%

**Table 2:** Leaf and leaflet parameters of the samples belonging to the PW16 groups designated in Figure S9.

Group No.	Leaf Fresh Weight (g)	Leaf Dry Weight (g)	Leaflets per Plant	Leaflets per Leaf
1	6.07 AB ^a^	1.53 AB	12.00 AB	4.50 A
2	6.36 AB	1.58 AB	13.29 AB	4.55 AB
3	5.36 AB	1.41 AB	11.00 B	4.05 B
4	5.59 AB	1.45 AB	14.00 AB	4.29 AB
5	6.88 AB	1.67 AB	12.57 AB	4.34 AB
6	5.75 AB	1.48 AB	13.50 AB	4.59 A
7	7.25 A	1.74 A	14.00 AB	4.65 A
8	5.17 AB	1.38 AB	14.67 AB	4.44 AB
9	5.19 AB	1.38 AB	13.73 AB	4.36 AB
10	5.11 AB	1.37 AB	12.33 AB	4.60 A
11	6.90 AB	1.68 AB	15.25 A	4.43 AB
12	6.13 AB	1.55 AB	14.00 AB	4.67 A
13	5.80 AB	1.49 AB	13.91 AB	4.41 AB
14	5.04 AB	1.36 AB	13.83 AB	4.39 AB
15	4.97 AB	1.34 AB	14.00 AB	4.37 AB
16	5.39 AB	1.41 AB	13.20 AB	4.61 A
17	5.91 AB	1.51 AB	13.60 AB	4.53 AB
18	5.48 AB	1.42 AB	14.50 AB	4.75 A
19	4.43 AB	1.25 AB	14.00 AB	4.67 A
20	5.12 AB	1.37 AB	14.50 AB	4.85 A
21	5.97 AB	1.52 AB	13.29 AB	4.51 AB
22	4.26 B	1.22 B	12.86 AB	4.39 AB

^a^ Values within a column followed by a letter in common are not significantly different at *p* = 0.05 according to Fisher’s protected Least Significant Difference test (LSD).

**Table 3:** Leaf petiole and stem parameters of the samples belonging to the PW16 groups designated in Figure S9.

Group No.	Leaf Petiole Fresh Weight (g)	Stem Length (cm)	Stem Fresh Weight (g)	Stem Dry Weight (g)
1	2.00 AB ^a^	30.50 ABCD	5.76 AB	0.81 AB
2	1.81 AB	31.31 ABCD	5.00 AB	0.76 AB
3	1.55 AB	30.82 ABCD	4.98 AB	0.69 AB
4	1.77 AB	31.15 ABCD	5.60 AB	0.76 AB
5	2.31 A	31.29 ABCD	5.82 AB	0.86 AB
6	1.82 AB	28.80 BCD	4.10 B	0.57 B
7	2.16 AB	35.66 A	7.02 A	1.04 A
8	1.67 AB	28.52 BCD	3.90 B	0.56 B
9	1.64 AB	28.33 BCD	4.32 B	0.66 AB
10	1.27 AB	27.60 CD	4.22 B	0.56 B
11	2.12 AB	32.78 ABC	5.51 AB	0.84 AB
12	1.97 AB	33.85 AB	4.96 AB	0.67 AB
13	1.57 AB	28.62 BCD	4.46 B	0.57 B
14	1.42 AB	29.80 BCD	3.73 B	0.49 B
15	1.73 AB	29.05 BCD	4.07 B	0.57 B
16	1.42 AB	28.36 BCD	4.42 B	0.59 B
17	1.55 AB	30.26 ABCD	4.77 AB	0.68 AB
18	1.49 AB	28.20 BCD	4.50 B	0.65 AB
19	1.23 B	26.63 D	3.63 B	0.58 B
20	1.61 AB	32.25 ABCD	4.68 AB	0.62 B
21	1.67 AB	29.21 BCD	4.34 B	0.65 AB
22	1.44 AB	29.00 BCD	3.94 B	0.51 B

^a^ Values within a column followed by a letter in common are not significantly different at *p* = 0.05 according to Fisher’s protected Least Significant Difference test (LSD).

**Table 4:** Seed petiole and petiolule parameters of the samples belonging to the PW16 groups designated in Figure S9.

Group No.	Seed Petiole Length (cm)	Seed Petiole Fresh Weight (g)	Seed petiole Dry Weight (g)	Seed Petiolules per Plant
1	13.43 A ^a^	0.67 AB	0.16 AB	49.00 AB
2	9.13 ABC	0.44 ABC	0.12 AB	40.36 ABCD
3	9.80 ABC	0.58 ABC	0.14 AB	40.33 ABCD
4	10.58 ABC	0.51 ABC	0.13 AB	54.42 A
5	12.76 AB	0.67 AB	0.16 AB	36.71 ABCD
6	11.30 AB	0.47 ABC	0.11 AB	44.50 ABC
7	12.70 AB	0.73 A	0.20 A	56.60 A
8	8.15 BC	0.32 BC	0.08 B	34.50 ABCD
9	9.65 ABC	0.39 ABC	0.10 AB	36.40 ABCD
10	10.67 ABC	0.38 ABC	0.10 AB	44.67 ABC
11	12.74 AB	0.60 ABC	0.16 AB	49.58 AB
12	10.70 ABC	0.32 BC	0.08 B	17.00 D
13	9.66 ABC	0.44 ABC	0.12 AB	37.36 ABCD
14	10.08 ABC	0.39 ABC	0.09 B	40.33 ABCD
15	11.88 AB	0.53 ABC	0.12 AB	52.75 A
16	11.26 AB	0.42 ABC	0.10 B	40.60 ABC
17	10.94 ABC	0.61 ABC	0.16 AB	55.40 A
18	10.10 ABC	0.51 ABC	0.12 AB	52.67 A
19	8.825 ABC	0.47 ABC	0.11 AB	57.25 A
20	6.30 C	0.27 C	0.07 B	25.50 CD
21	10.55 ABC	0.45 ABC	0.12 AB	43.86 ABC
22	11.27 AB	0.25 C	0.06 B	27.29 BCD

^a^ Values within a column followed by a letter in common are not significantly different at *p* = 0.05 according to Fisher’s protected Least Significant Difference test (LSD).

**Table 5:** Seed parameters of the samples belonging to the PW16 groups designated in Figure S9.

Group No.	Red Seeds per Plant	Green Seeds per Plant	Total Seeds per Plant	Seed Fresh Weight (g)	Seed Dry Weight (g)
1	6.67 AB ^a^	4.67 C	11.33 BCD	1.24 BC	0.33 ABC
2	3.36 AB	13.21 ABC	16.57 ABCD	2.43 ABC	0.56 ABC
3	8.33 AB	18.17 ABC	26.50 ABC	3.77 A	0.77 A
4	11.37 A	17.37 ABC	28.74 AB	2.55 ABC	0.55 ABC
5	5.86 AB	14.71 ABC	20.57 ABCD	3.28 AB	0.61 AB
6	1.75 B	9.25 C	11.00 BCD	1.46 ABC	0.26 ABC
7	6.80 AB	11.80 BC	18.60 ABCD	2.23 ABC	0.39 ABC
8	2.67 AB	8.17 C	10.83 CD	1.43 ABC	0.31 ABC
9	5.20 AB	15.47 ABC	20.67 ABCD	2.25 ABC	0.45 ABC
10	0.00 B	7.33 C	7.33 D	0.23 C	0.04 C
11	6.58 AB	10.67 C	17.25 ABCD	1.84 ABC	0.40 ABC
12	8.00 AB	5.00 C	13.00 BCD	1.87 ABC	0.31 ABC
13	6.18 AB	12.18 ABC	18.36 ABCD	1.67 ABC	0.33 ABC
14	4.00 AB	11.50 C	15.50 ABCD	1.56 ABC	0.34 ABC
15	1.75 B	26.75 A	28.50 ABC	1.79 ABC	0.39 ABC
16	5.80 AB	12.80 ABC	18.60 ABCD	1.41 ABC	0.25 ABC
17	4.80 AB	11.00 C	15.80 ABCD	1.37 ABC	0.33 ABC
18	5.00 AB	26.50 AB	31.50 A	2.20 ABC	0.47 ABC
19	2.00 AB	6.25 C	8.25 D	0.77 C	0.17 BC
20	4.50 AB	10.25 C	14.75 ABCD	0.82 BC	0.13 BC
21	6.29 AB	9.57 C	15.86 ABCD	1.78 ABC	0.38 ABC
22	3.71 AB	3.43 C	7.14 D	0.45 C	0.24 ABC

^a^ Values within a column followed by a letter in common are not significantly different at *p* = 0.05 according to Fisher’s protected Least Significant Difference test (LSD).

## Data Availability

The data that support the findings of this study are available on request from the corresponding author (P.H.G). (Email address: pgoodwin@uoguelph.ca). The data are not publicly available due to their being used in an ongoing project.

## Supplementary Materials

The following supporting information can be downloaded at: https://www.mdpi.com/article/10.3390/plants13020300/s1, Figure S1: Phenotypic correlation coefficients between root fresh weight with leaf fresh weight, stem fresh weight, or seed petioles dry weight. Figure S2: Phenotypic correlation coefficients between root dry weight with leaf dry weight, leaf petiole dry weight, stem dry weight, or seed petioles dry weight. Figure S3: Phenotypic correlation coefficients between leaf fresh weight with leaf area or leaf petiole fresh weight. Figure S4: Phenotypic correlation coefficients between leaf dry weight with leaf area, leaf petioles dry weight, stem dry weight, or seed petioles dry weight. Figure S5: Phenotypic correlation coefficients between leaf petioles dry weight with stem dry weight or seed petioles dry weight. Figure S6: Phenotypic correlation coefficients between stem length with stem fresh weight or stem dry weight. Figure S7: Phenotypic correlation coefficients between stem dry weight with seed petioles dry weight. Figure S8: Alignment using MUSCLE of PW16 sequences of 162 *P. quinquefolius* from a commercial ginseng garden near Simcoe, Ontario, Canada. Figure S9: Neighbor-joining tree constructed with sequences from 162 *P. quinquefolius* from a commercial ginseng garden near Simcoe, Ontario, Canada, in 2019. The scale bar shows 0.01 changes, and bootstrap support values from 1000 replicates are shown at the nodes. Table S1: Root parameters separated by root grade. Table S2: Leaf parameters separated by root grade. Table S3: Petiole and stem parameters separated by root grade. Table S4: Seed parameters separated by root grade. Table S5: Root, leaf, and leaf petiole parameters of the samples belonging to the PW16 groups designated in Figure S9.
