# Supplementary material for: Phenotypic and Genotypic Variation of Cultivated Panax quinquefolius
Source: Plants (Basel). 2024 Jan 19;13(2):300. doi: 10.3390/plants13020300 (PMC10821518; doi:10.3390/plants13020300)
Supplement: Supplementary file 1 [file plants-13-00300-s001.zip › plants-2779648-supplementary.pdf]

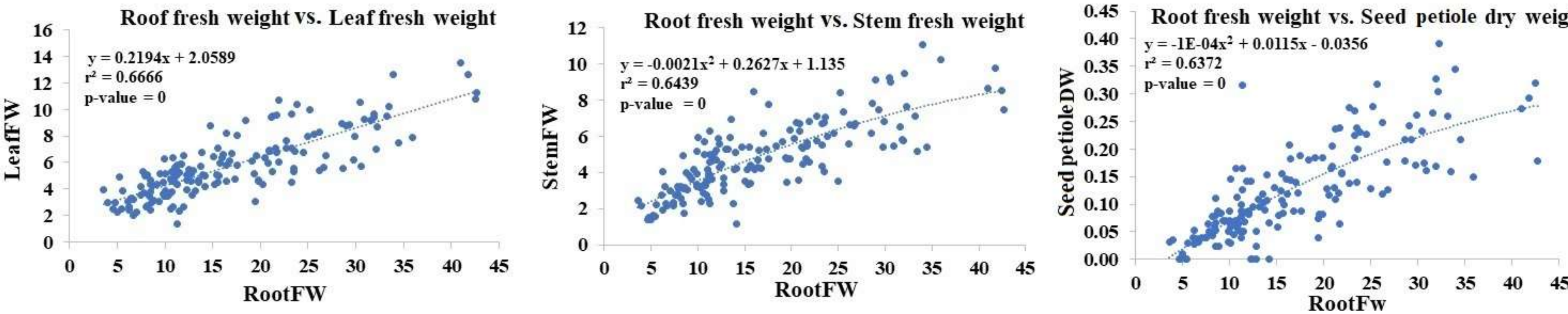

Figure S1. Correlation between root fresh weight and leaf fresh weight, root fresh weight and stem fresh weight, and root fresh weight and seed petiole dry weight for the 162 *P. quinquefolius* samples. Correlation equation, coefficients and p-value is presented in each graph.

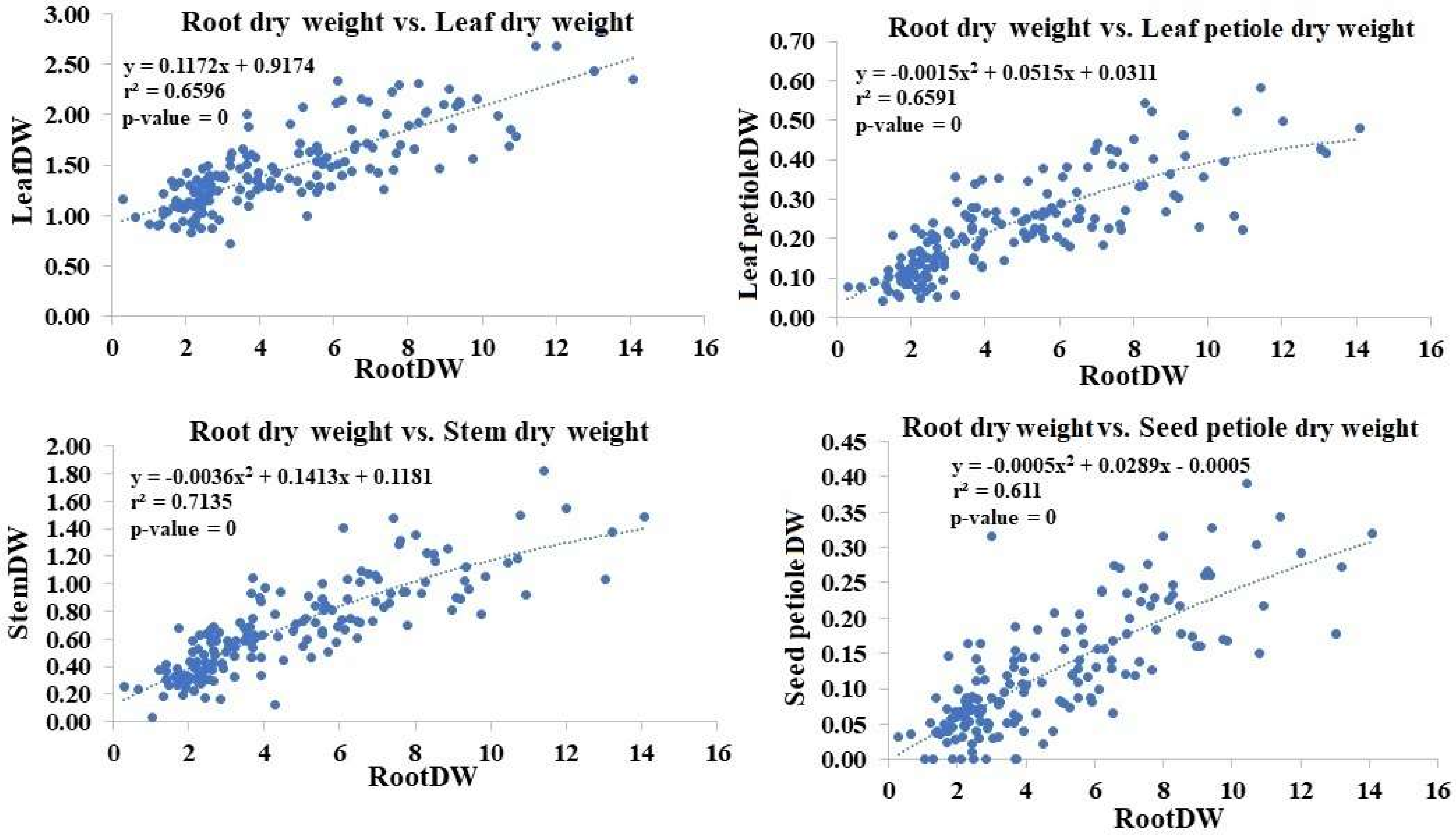

Figure S2. Correlation between root dry weight and leaf dry weight, root dry weight and leaf petiole dry weight, root dry weight and stem dry weight, and root dry weight and seed petiole dry weight for the 162 *P. quinquefolius* samples. Correlation equation, coefficients and p-value is presented in each graph.

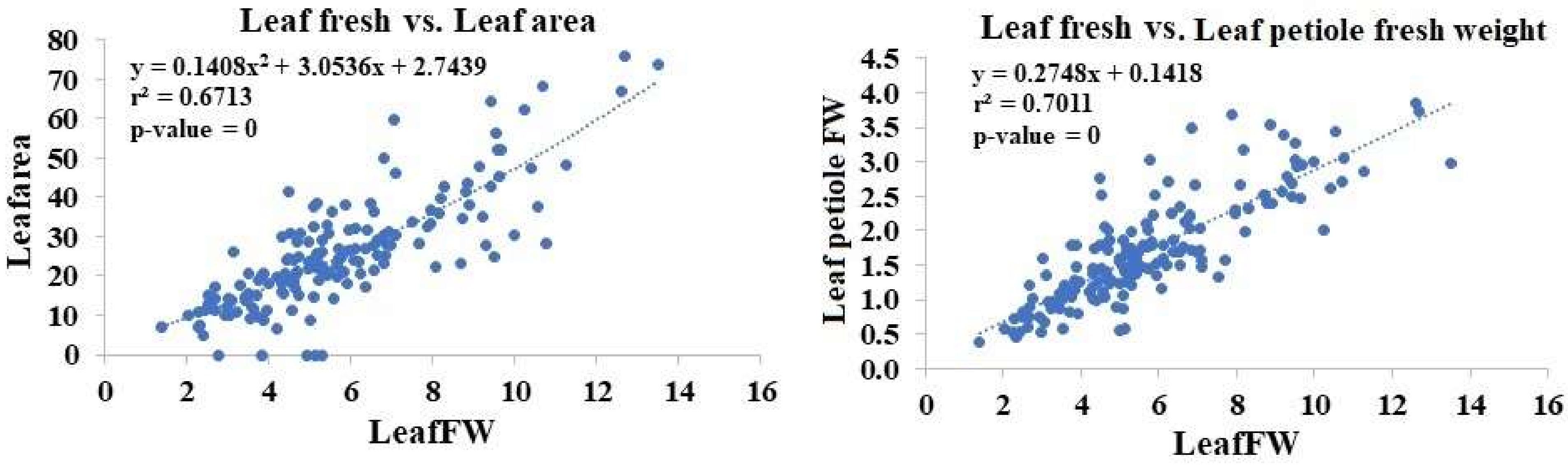

Figure S3. Correlation between leaf fresh weight and leaf area, and leaf fresh weight and leaf petiole fresh weight for the 162 *P. quinquefolius* samples. Correlation equation, coefficients and p-value is presented in each graph.

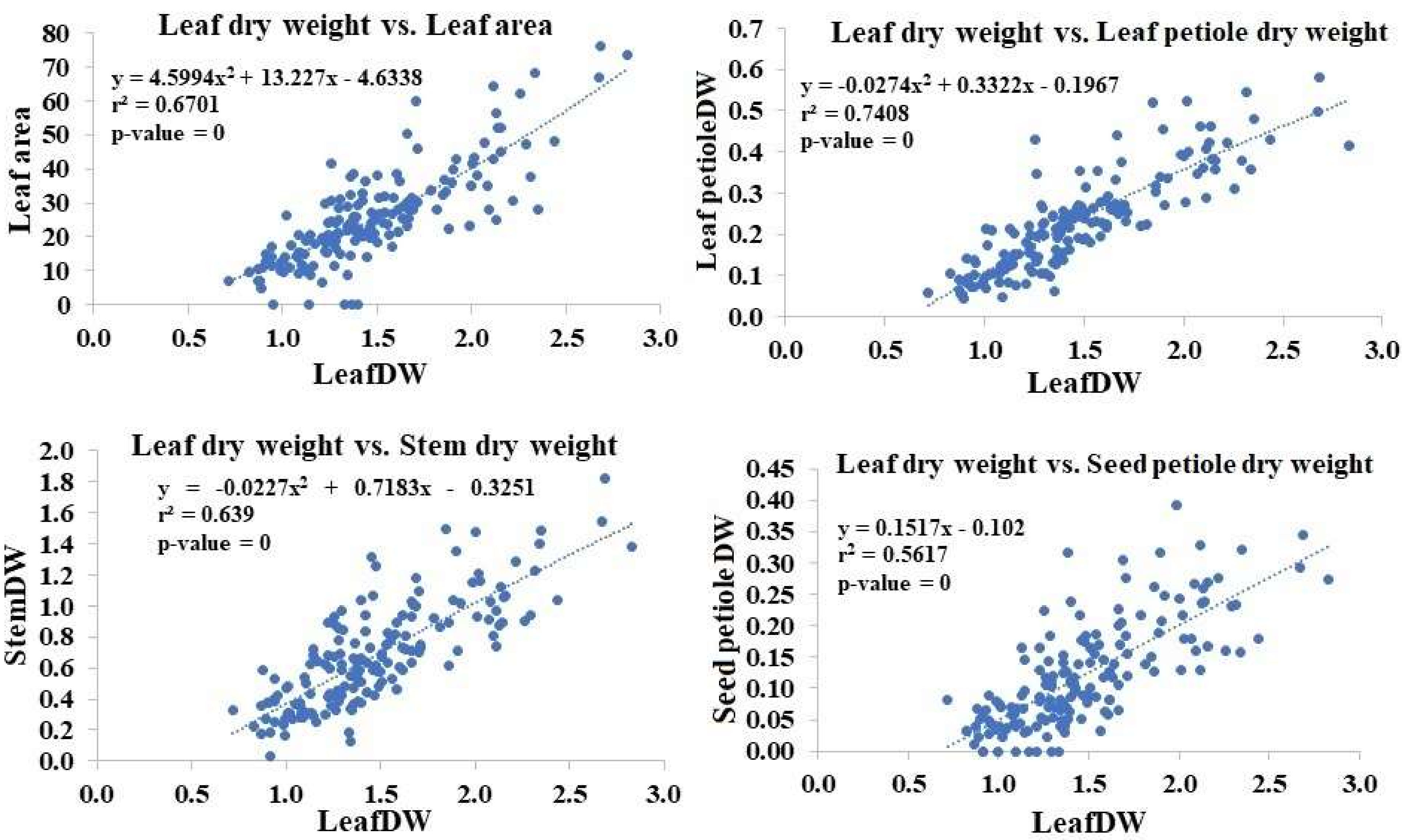

Figure S4. Correlation between leaf dry weight and leaf are, leaf dry weight and leaf petiole dry weight, leaf dry weight and stem dry weight, and leaf dry weight and seed petiole dry weight for the 162 *P. quinquefolius* samples. Correlation equation, coefficients and p-value is presented in each graph.

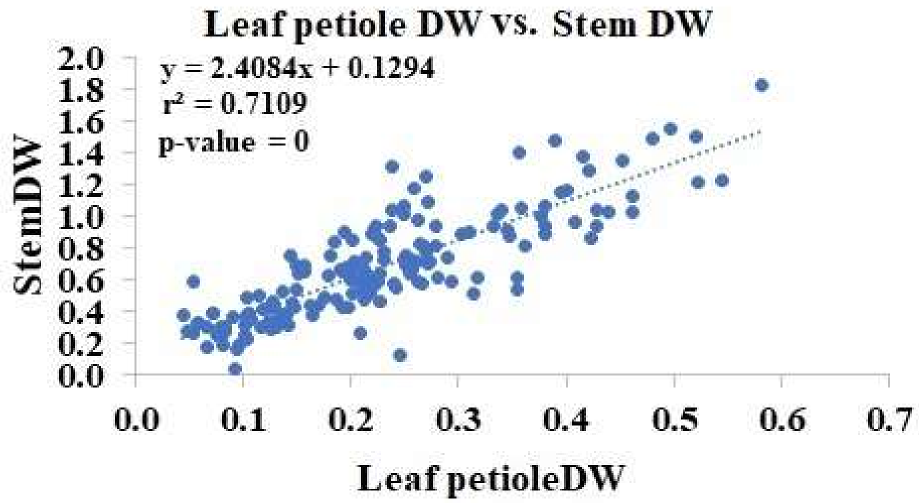

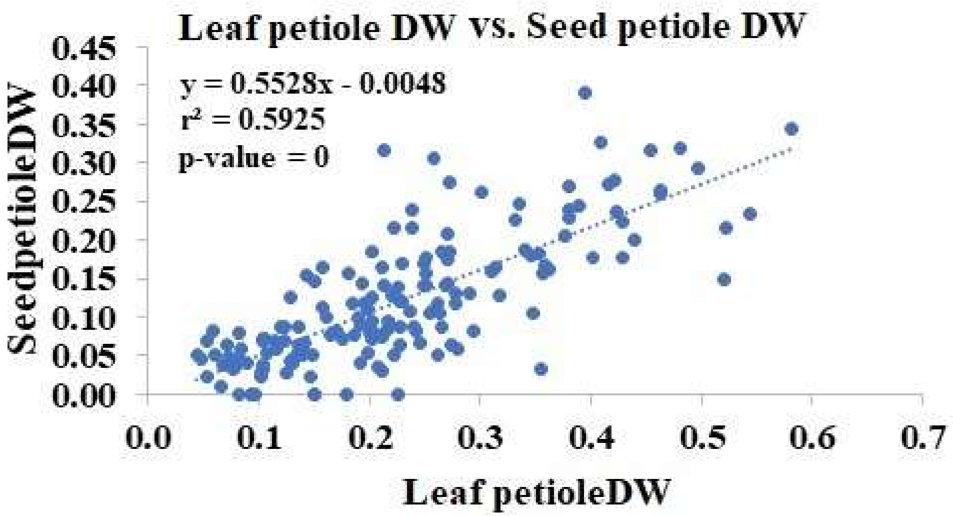

Figure S5. Correlation between leaf petiole dry weight and stem dry weight, and leaf petiole dry weight and seed petiole dry weight for the 162 *P. quinquefolius* samples. Correlation equation, coefficients and p-value is presented in each graph.

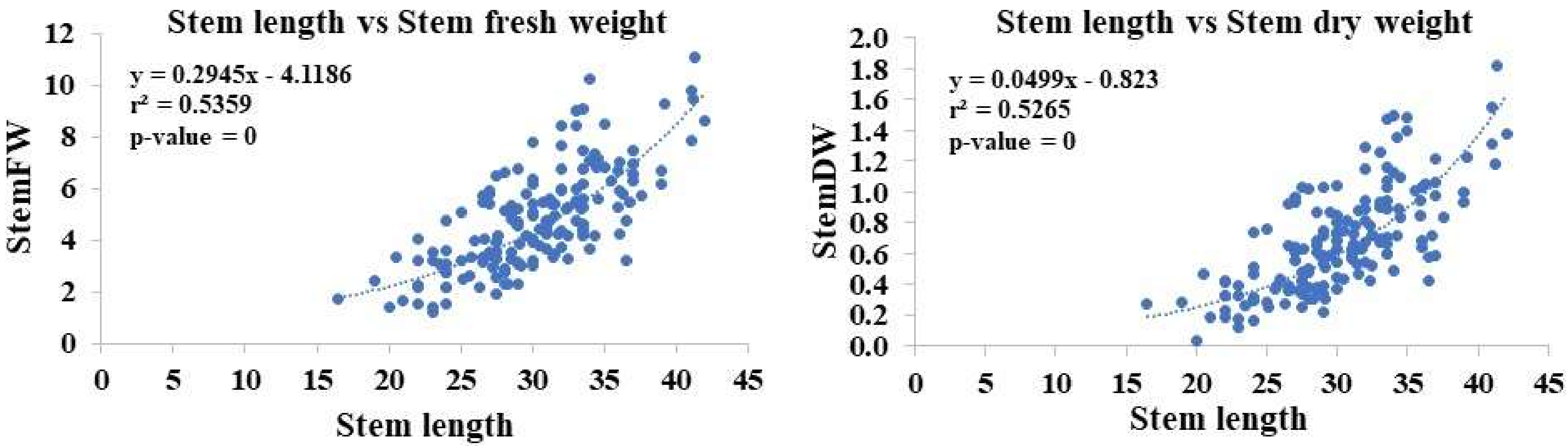

Figure S6. Correlation between stem length and stem fresh weight, and stem length and stem dry weight for the 162 *P. quinquefolius* samples. Correlation equation, coefficients and p-value is presented in each graph.

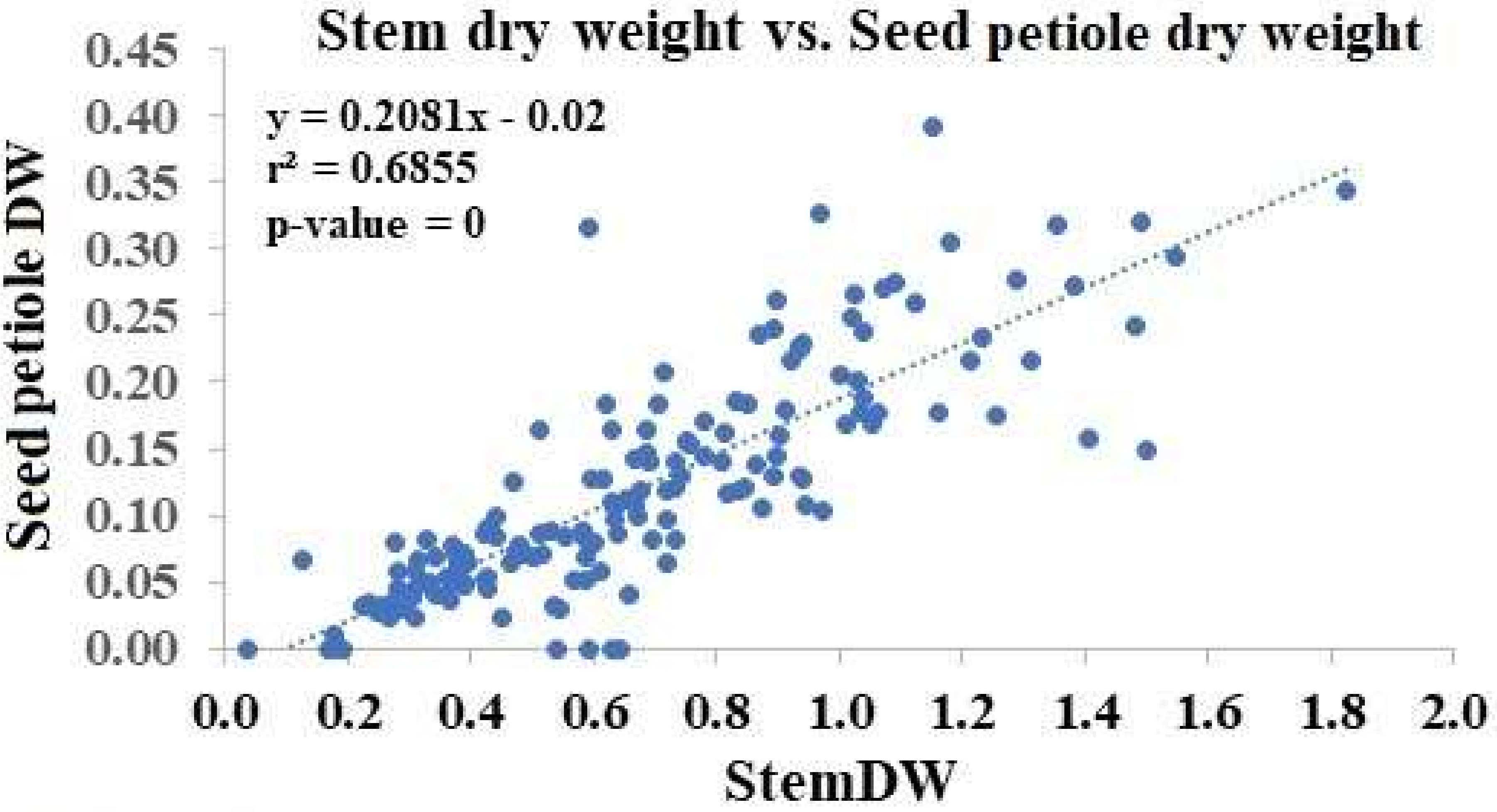

Figure S7. Correlation between stem dry weight and seed petiole dry weight for the 162 *P. quinquefolius* samples. Correlation equation, coefficients and p-value is presented in each graph.

Figure S8. Alignment using MUSCLE of PW16 sequences of 162 *P. quinquefolius* samples from a commercial ginseng garden near Simcoe, Ontario, Canada.

```
172                 CTCCTGCCAAAGAAATTCAAGCGTCCTCACAAAGGGTTTAGTGCGCATCTCCCATCTTTG
229                 CTCCTGCCAAAGAAATTCAAGCGTCCTCACAAAGGGTTTAGTGCGCATCTCCCATCTTTG
208                 CTCCTGCCAAAGAAATTCAAGCGTCCTCACAAAGGGTTTAGTGCGCATCTCCCATCTTTG
83                  CTCCTGCCAAAGAAATTCAAGCGTCCTCACAAAGGGTTTAGTGCGCATCTCCCATCTTTT
3                   CTCCTGCCAAAGAAATTCAAGCGTCCTCACAAAGGGTTTAGTGCGCATCTCCCATCTTTT
120                 CTCCTGCCAAAGAAATTCAAGCGTCCTCACAAAGGGTTTAGTGCGCATCTCCCATTTTGT
99                  CTCCTGCCAAAGAAATTCAAGCGTCCTCACAAAGGGTTTAGTGCGCATCTCCCATCTTGT
126                 CTCCTGCCAAAGAAATTCAAGCGTCCTCACAAAGGGTTTAGTGCGCATCTCCTAATTTTT
173                 CTCCTGCCAAAGAAATTCAAGCGTCCTCACAAAGGGTTTAGTGCGCATCTCCTAATTTTT
204                 CTCCTGCCAAAGAAATTCAAGCGTCCTCACAAAGGGTTTAGTGCGCATCTACCAATTTTT
239                 CTCCTGCCAAAGAAATTCAAGCGTCCTCACAAAGGGTTTAGTGCGCATCTCCTATCTTGT
117                 CTCCTGCCAAAGAAATTCAAGCGTCCTCACAAAGGGTTTAGTGCGCATCTACCAACTCGT
123                 CTCCTGCCAAAGAAATTCAAGCGTCCTCACAAAGGGTTTAGTGCGCATCTACCAACTCGT
210                 CTCCTGCCAAAGAAATTCAAGCGTCCTCACAAAGGGTTTAGTGCGCATCTCCCATCTTGT
86                  CTCCTGCCAAAGAAATTCAAGCGTCCTCACAAAGGGTTTAGTGCGCATCTCCCATCTTGT
100                 CTCCTGCCAAAGAAATTCAAGCGTCCTCACAAAGGGTTTAGTGCGCATCTCCCATCTTGT
114                 CTCCTGCCAAAGAAATTCAAGCGTCCTCACAAAGGGTTTAGTGCGCATCTCCCAACTCGT
170                 CTCCTGCCAAAGAAATTCAAGCGTCCTCACAAAGGGTTTAGTGCGCATCTCCCAACTCGT
108                 CTCCTGCCAAAGAAATTCAAGCGTCCTCACAAAGGGTTTAGTGCGCATCTCCCATCTTGT
174                 CTCCTGCCAAAGAAATTCAAGCGTCCTCACAAAGGGTTTAGTGCGCATCTCCCATCTTGT
158                 CTCCTGCCAAAGAAATTCAAGCGTCCTCACAAAGGGTTTAGTGCGCATCTCCCATCTTGT
154                 CTCCTGCCAAAGAAATTCAAGCGTCCTCACAAAGGGTTTAGTGCGCATCTCCCATCTTGG
30                  CTCCTGCCAAAGAAATTCAAGCGTCCTCACAAAGGGTTTAGTGCGCATCTCCCATCTTGT
39                  CTCCTGCCAAAGAAATTCAAGCGTCCTCACAAAGGGTTTAGTGCGCATCTCCCATCTTGT
237                 CTCCTGCCAAAGAAATTCAAGCGTCCTCACAAAGGGTTTAGTGCGCATCTCCCATCTTGT
65                  CTCCTGCCAAAGAAATTCAAGCGTCCTCACAAAGGGTTTAGTGCGCATCTCCCATCTTGT
44                  CTCCTGCCAAAGAAATTCAAGCGTCCTCACAAAGGGTTTAGTGCGCATCTACTATCTTGT
168                 CTCCTGCCAAAGAAATTCAAGCGTCCTCACAAAGGGTTTAGTGCGCATCTCCCATCTTGT
95                  CTCCTGCCAAAGAAATTCAAGCGTCCTCACAAAGGGTTTAGTGCGCATCTCCCATCTTGT
22                  CTCCTGCCAAAGAAATTCAAGCGTCCTCACAAAGGGTTTAGTGCGCATCTCCCATCTTGT
79                  CTCCTGCCAAAGAAATTCAAGCGTCCTCACAAAGGGTTTAGTGCGCATCTCCCATCTTGT
211                 CTCCTGCCAAAGAAATTCAAGCGTCCTCACAAAGGGTTTAGTGCGCATCTCCCATCTTGT
88                  CTCCTGCCAAAGAAATTCAAGCGTCCTCACAAAGGGTTTAGTGCGCATCTCCCATCTTGT
184                 CTCCTGCCAAAGAAATTCAAGCGTCCTCACAAAGGGTTTAGTGCGCATCTCCCATCTTGT
78                  CTCCTGCCAAAGAAATTCAAGCGTCCTCACAAAGGGTTTAGTGCGCATCTCCCATCTTGT
71                  CTCCTGCCAAAGAAATTCAAGCGTCCTCACAAAGGGTTTAGTGCGCATCTCCCATCTTGT
196                 CTCCTGCCAAAGAAATTCAAGCGTCCTCACAAAGGGTTTAGTGCGCATCTCCCATCTTGT
43                  CTCCTGCCAAAGAAATTCAAGCGTCCTCACAAAGGGTTTAGTGCGCATCTCCCATCTTGT
151                 CTCCTGCCAAAGAAATTCAAGCGTCCTCACAAAGGGTTTAGTGCGCATCTCCCATCTTGT
27                  CTCCTGCCAAAGAAATTCAAGCGTCCTCACAAAGGGTTTAGTGCGCATCTCCCATCTTGT
197                 CTCCTGCCAAAGAAATTCAAGCGTCCTCACAAAGGGTTTAGTGCGCATCTCCCATCTTGT
109                 CTCCTGCCAAAGAAATTCAAGCGTCCTCACAAAGGGTTTAGTGCGCATCTCCCATCTTGT
169                 CTCCTGCCAAAGAAATTCAAGCGTCCTCACAAAGGGTTTAGTGCGCATCTCCCATCTTGT
94                  CTCCTGCCAAAGAAATTCAAGCGTCCTCACAAAGGGTTTAGTGCGCATCTCCCATCTTGT
232                 CTCCTGCCAAAGAAATTCAAGCGTCCTCACAAAGGGTTTAGTGCGCATCTCCCATCTTGT
222                 CTCCTGCCAAAGAAATTCAAGCGTCCTCACAAAGGGTTTAGTGCGCATCTCCCATCTTGT
24                  CTCCTGCCAAAGAAATTCAAGCGTCCTCACAAAGGGTTTAGTGCGCATCTCCCATCTTGT
15                  CTCCTGCCAAAGAAATTCAAGCGTCCTCACAAAGGGTTTAGTGCGCATCTCCCATCTTGT
13                  CTCCTGCCAAAGAAATTCAAGCGTCCTCACAAAGGGTTTAGTGCGCATCTCCCATCTTGT
14                  CTCCTGCCAAAGAAATTCAAGCGTCCTCACAAAGGGTTTAGTGCGCATCTCCCATCTTGT
35                  CTCCTGCCAAAGAAATTCAAGCGTCCTCACAAAGGGTTTAGTGCGCATCTCCCATCTTGT
111                 CTCCTGCCAAAGAAATTCAAGCGTCCTCACAAAGGGTTTAGTGCGCATCTCCCATCTTGT
157                 CTCCTGCCAAAGAAATTCAAGCGTCCTCACAAAGGGTTTAGTGCGCATCTCCCATCTTGT
```

31	CTCCTGCCAAAGAAATTCAAGCGTCCTCACAAAGGGTTTAGTGCGCATCTCCCATCTTGT
47	CTCCTGCCAAAGAAATTCAAGCGTCCTCACAAAGGGTTTAGTGCGCATCTCCCATCTTGT
33	CTCCTGCCAAAGAAATTCAAGCGTCCTCACAAAGGGTTTAGTGCGCATCTCCCATCTTGT
51	CTCCTGCCAAAGAAATTCAAGCGTCCTCACAAAGGGTTTAGTGCGCATCTCCCATCTTGT
85	CTCCTGCCAAAGAAATTCAAGCGTCCTCACAAAGGGTTTAGTGCGCATCTCCCATCTTGT
54	CTCCTGCCAAAGAAATTCAAGCGTCCTCACAAAGGGTTTAGTGCGCATCTCCCATCTTGT
96	CTCCTGCCAAAGAAATTCAAGCGTCCTCACAAAGGGTTTAGTGCGCATCTCCCATCTTGT
61	CTCCTGCCAAAGAAATTCAAGCGTCCTCACAAAGGGTTTAGTGCGCATCTCCCATCTTGT
80	CTCCTGCCAAAGAAATTCAAGCGTCCTCACAAAGGGTTTAGTGCGCATCTCCCATCTTGT
82	CTCCTGCCAAAGAAATTCAAGCGTCCTCACAAAGGGTTTAGTGCGCATCTCCCATCTTGT
129	CTCCTGCCAAAGAAATTCAAGCGTCCTCACAAAGGGTTTAGTGCGCATCTCCCATCTTGT
46	CTCCTGCCAAAGAAATTCAAGCGTCCTCACAAAGGGTTTAGTGCGCATCTCCCATCTTGT
74	CTCCTGCCAAAGAAATTCAAGCGTCCTCACAAAGGGTTTAGTGCGCATCTCCCATCTTGT
101	CTCCTGCCAAAGAAATTCAAGCGTCCTCACAAAGGGTTTAGTGCGCATCTCCCATCTTGT
66	CTCCTGCCAAAGAAATTCAAGCGTCCTCACAAAGGGTTTAGTGCGCATCTCCCATCTTGT
104	CTCCTGCCAAAGAAATTCAAGCGTCCTCACAAAGGGTTTAGTGCGCATCTCCCATCTTGT
118	CTCCTGCCAAAGAAATTCAAGCGTCCTCACAAAGGGTTTAGTGCGCATCTCCCATCTTGT
21	CTCCTGCCAAAGAAATTCAAGCGTCCTCACAAAGGGTTTAGTGCGCATCTCCCATCTTGT
77	CTCCTGCCAAAGAAATTCAAGCGTCCTCACAAAGGGTTTAGTGCGCATCTCCCATCTTGT
124	CTCCTGCCAAAGAAATTCAAGCGTCCTCACAAAGGGTTTAGTGCGCATCTCCCATCTTGT
20	CTCCTGCCAAAGAAATTCAAGCGTCCTCACAAAGGGTTTAGTGCGCATCTCCCATCTTGT
110	CTCCTGCCAAAGAAATTCAAGCGTCCTCACAAAGGGTTTAGTGCGCATCTCCCATCTTGT
70	CTCCTGCCAAAGAAATTCAAGCGTCCTCACAAAGGGTTTAGTGCGCATCTCCCATCTTGT
8	CTCCTGCCAAAGAAATTCAAGCGTCCTCACAAAGGGTTTAGTGCGCATCTCCCATCTTGT
55	CTCCTGCCAAAGAAATTCAAGCGTCCTCACAAAGGGTTTAGTGCGCATCTCCCATCTTGT
64	CTCCTGCCAAAGAAATTCAAGCGTCCTCACAAAGGGTTTAGTGCGCATCTCCCATCTTGT
93	CTCCTGCCAAAGAAATTCAAGCGTCCTCACAAAGGGTTTAGTGCGCATCTCCCATCTTGT
202	CTCCTGCCAAAGAAATTCAAGCGTCCTCACAAAGGGTTTAGTGCGCATCTCCCATCTTGT
10	CTCCTGCCAAAGAAATTCAAGCGTCCTCACAAAGGGTTTAGTGCGCATCTCCCATCTTGT
16	CTCCTGCCAAAGAAATTCAAGCGTCCTCACAAAGGGTTTAGTGCGCATCTCCCATCTTGT
76	CTCCTGCCAAAGAAATTCAAGCGTCCTCACAAAGGGTTTAGTGCGCATCTCCCATCTTGT
116	CTCCTGCCAAAGAAATTCAAGCGTCCTCACAAAGGGTTTAGTGCGCATCTCCCATCTTGT
220	CTCCTGCCAAAGAAATTCAAGCGTCCTCACAAAGGGTTTAGTGCGCATCTCCCATCTTGT
234	CTCCTGCCAAAGAAATTCAAGCGTCCTCACAAAGGGTTTAGTGCGCATCTCCCATCTTGT
2	CTCCTGCCAAAGAAATTCAAGCGTCCTCACAAAGGGTTTAGTGCGCATCTCCCATCTTGT
38	CTCCTGCCAAAGAAATTCAAGCGTCCTCACAAAGGGTTTAGTGCGCATCTCCCATCTTGT
73	CTCCTGCCAAAGAAATTCAAGCGTCCTCACAAAGGGTTTAGTGCGCATCTCCCATCTTGT
87	CTCCTGCCAAAGAAATTCAAGCGTCCTCACAAAGGGTTTAGTGCGCATCTCCCATCTTGT
92	CTCCTGCCAAAGAAATTCAAGCGTCCTCACAAAGGGTTTAGTGCGCATCTCCCATCTTGT
98	CTCCTGCCAAAGAAATTCAAGCGTCCTCACAAAGGGTTTAGTGCGCATCTCCCATCTTGT
137	CTCCTGCCAAAGAAATTCAAGCGTCCTCACAAAGGGTTTAGTGCGCATCTCCCATCTTGT
19	CTCCTGCCAAAGAAATTCAAGCGTCCTCACAAAGGGTTTAGTGCGCATCTCCCATCTTGT
171	CTCCTGCCAAAGAAATTCAAGCGTCCTCACAAAGGGTTTAGTGCGCATCTCCCATCTTGT
205	CTCCTGCCAAAGAAATTCAAGCGTCCTCACAAAGGGTTTAGTGCGCATCTCCCATCTTGT
75	CTCCTGCCAAAGAAATTCAAGCGTCCTCACAAAGGGTTTAGTGCGCATCTCCCATCTTGT
62	CTCCTGCCAAAGAAATTCAAGCGTCCTCACAAAGGGTTTAGTGCGCATCTCCCATCTTGT
4	CTCCTGCCAAAGAAATTCAAGCGTCCTCACAAAGGGTTTAGTGCGCATCTCCCATCTTGT
7	CTCCTGCCAAAGAAATTCAAGCGTCCTCACAAAGGGTTTAGTGCGCATCTCCCATCTTGT
25	CTCCTGCCAAAGAAATTCAAGCGTCCTCACAAAGGGTTTAGTGCGCATCTCCCATCTTGT
28	CTCCTGCCAAAGAAATTCAAGCGTCCTCACAAAGGGTTTAGTGCGCATCTCCCATCTTGT
36	CTCCTGCCAAAGAAATTCAAGCGTCCTCACAAAGGGTTTAGTGCGCATCTCCCATCTTGT
49	CTCCTGCCAAAGAAATTCAAGCGTCCTCACAAAGGGTTTAGTGCGCATCTCCCATCTTGT
145	CTCCTGCCAAAGAAATTCAAGCGTCCTCACAAAGGGTTTAGTGCGCATCTCCCATCTTGT
181	CTCCTGCCAAAGAAATTCAAGCGTCCTCACAAAGGGTTTAGTGCGCATCTCCCATCTTGT
188	CTCCTGCCAAAGAAATTCAAGCGTCCTCACAAAGGGTTTAGTGCGCATCTCCCATCTTGT
59	CTCCTGCCAAAGAAATTCAAGCGTCCTCACAAAGGGTTTAGTGCGCATCTCCCATCTTGT
121	CTCCTGCCAAAGAAATTCAAGCGTCCTCACAAAGGGTTTAGTGCGCATCTCCCATCTTGT

```
127    CTCCTGCCAAAGAAATTCAAGCGTCCTCACAAAGGGTTTAGTGCGCATCTCCCATCTTGT
135    CTCCTGCCAAAGAAATTCAAGCGTCCTCACAAAGGGTTTAGTGCGCATCTCCCATCTTGT
136    CTCCTGCCAAAGAAATTCAAGCGTCCTCACAAAGGGTTTAGTGCGCATCTCCCATCTTGT
29     CTCCTGCCAAAGAAATTCAAGCGTCCTCACAAAGGGTTTAGTGCGCATCTCCCATCTTGT
89     CTCCTGCCAAAGAAATTCAAGCGTCCTCACAAAGGGTTTAGTGCGCATCTCCCATCTTGT
216    CTCCTGCCAAAGAAATTCAAGCGTCCTCACAAAGGGTTTAGTGCGCATCTCCCATCTTGT
164    CTCCTGCCAAAGAAATTCAAGCGTCCTCACAAAGGGTTTAGTGCGCATCTCCCATCTTGT
63     CTCCTGCCAAAGAAATTCAAGCGTCCTCACAAAGGGTTTAGTGCGCATCTCCCATCTTGT
161    CTCCTGCCAAAGAAATTCAAGCGTCCTCACAAAGGGTTTAGTGCGCATCTCCCATCTTGT
183    CTCCTGCCAAAGAAATTCAAGCGTCCTCACAAAGGGTTTAGTGCGCATCTCCCATCTTGT
18     CTCCTGCCAAAGAAATTCAAGCGTCCTCACAAAGGGTTTAGTGCGCATCTCCCATCTTGT
90     CTCCTGCCAAAGAAATTCAAGCGTCCTCACAAAGGGTTTAGTGCGCATCTCCCATCTTGT
162    CTCCTGCCAAAGAAATTCAAGCGTCCTCACAAAGGGTTTAGTGCGCATCTCCCATCTTGT
177    CTCCTGCCAAAGAAATTCAAGCGTCCTCACAAAGGGTTTAGTGCGCATCTCCCATCTTGT
97     CTCCTGCCAAAGAAATTCAAGCGTCCTCACAAAGGGTTTAGTGCGCATCTCCCATCTTGT
231    CTCCTGCCAAAGAAATTCAAGCGTCCTCACAAAGGGTTTAGTGCGCATCTCCCATCTTGT
143    CTCCTGCCAAAGAAATTCAAGCGTCCTCACAAAGGGTTTAGTGCGCATCTCCCATCTTGT
11     CTCCTGCCAAAGAAATTCAAGCGTCCTCACAAAGGGTTTAGTGCGCATCTCCCATCTTGT
153    CTCCTGCCAAAGAAATTCAAGCGTCCTCACAAAGGGTTTAGTGCGCATCTCCCATCTTGT
138    CTCCTGCCAAAGAAATTCAAGCGTCCTCACAAAGGGTTTAGTGCGCATCTCCCATCTTGT
185    CTCCTGCCAAAGAAATTCAAGCGTCCTCACAAAGGGTTTAGTGCGCATCTCCCATCTTGT
194    CTCCTGCCAAAGAAATTCAAGCGTCCTCACAAAGGGTTTAGTGCGCATCTCCCATCTTGT
236    CTCCTGCCAAAGAAATTCAAGCGTCCTCACAAAGGGTTTAGTGCGCATCTCCCATCTTGT
32     CTCCTGCCAAAGAAATTCAAGCGTCCTCACAAAGGGTTTAGTGCGCATCTCCCATCTTGT
52     CTCCTGCCAAAGAAATTCAAGCGTCCTCACAAAGGGTTTAGTGCGCATCTCCCATCTTGT
60     CTCCTGCCAAAGAAATTCAAGCGTCCTCACAAAGGGTTTAGTGCGCATCTCCCATCTTGT
72     CTCCTGCCAAAGAAATTCAAGCGTCCTCACAAAGGGTTTAGTGCGCATCTCCCATCTTGT
112    CTCCTGCCAAAGAAATTCAAGCGTCCTCACAAAGGGTTTAGTGCGCATCTCCCATCTTGT
141    CTCCTGCCAAAGAAATTCAAGCGTCCTCACAAAGGGTTTAGTGCGCATCTCCCATCTTGT
155    CTCCTGCCAAAGAAATTCAAGCGTCCTCACAAAGGGTTTAGTGCGCATCTCCCATCTTGT
1      CTCCTGCCAAAGAAATTCAAGCGTCCTCACAAAGGGTTTAGTGCGCATCTCCCATCTTGT
6      CTCCTGCCAAAGAAATTCAAGCGTCCTCACAAAGGGTTTAGTGCGCATCTCCCATCTTGT
58     CTCCTGCCAAAGAAATTCAAGCGTCCTCACAAAGGGTTTAGTGCGCATCTCCCATCTTGT
68     CTCCTGCCAAAGAAATTCAAGCGTCCTCACAAAGGGTTTAGTGCGCATCTCCCATCTTGT
180    CTCCTGCCAAAGAAATTCAAGCGTCCTCACAAAGGGTTTAGTGCGCATCTCCCATCTTGT
81     CTCCTGCCAAAGAAATTCAAGCGTCCTCACAAAGGGTTTAGTGCGCATCTCCCATCTTGT
103    CTCCTGCCAAAGAAATTCAAGCGTCCTCACAAAGGGTTTAGTGCGCATCTCCCATCTTGT
113    CTCCTGCCAAAGAAATTCAAGCGTCCTCACAAAGGGTTTAGTGCGCATCTCCCATCTTGT
163    CTCCTGCCAAAGAAATTCAAGCGTCCTCACAAAGGGTTTAGTGCGCATCTCCCATCTTGT
40     CTCCTGCCAAAGAAATTCAAGCGTCCTCACAAAGGGTTTAGTGCGCATCTCCCATCTTGT
56     CTCCTGCCAAAGAAATTCAAGCGTCCTCACAAAGGGTTTAGTGCGCATCTCCCATCTTGT
218    CTCCTGCCAAAGAAATTCAAGCGTCCTCACAAAGGGTTTAGTGCGCATCTCCCATCTTGT
41     CTCCTGCCAAAGAAATTCAAGCGTCCTCACAAAGGGTTTAGTGCGCATCTCCCATCTTGT
42     CTCCTGCCAAAGAAATTCAAGCGTCCTCACAAAGGGTTTAGTGCGCATCTCCCATCTTGT
160    CTCCTGCCAAAGAAATTCAAGCGTCCTCACAAAGGGTTTAGTGCGCATCTCCCATCTTGT
5      CTCCTGCCAAAGAAATTCAAGCGTCCTCACAAAGGGTTTAGTGCGCATCTCCCATCTTGT
228    CTCCTGCCAAAGAAATTCAAGCGTCCTCACAAAGGGTTTAGTGCGCATCTCCCATCTTGT
17     CTCCTGCCAAAGAAATTCAAGCGTCCTCACAAAGGGTTTAGTGCGCATCTCCCATCTTGT
37     CTCCTGCCAAAGAAATTCAAGCGTCCTCACAAAGGGTTTAGTGCGCATCTCCCATCTTGT
91     CTCCTGCCAAAGAAATTCAAGCGTCCTCACAAAGGGTTTAGTGCGCATCTCCCATCTTGT
140    CTCCTGCCAAAGAAATTCAAGCGTCCTCACAAAGGGTTTAGTGCGCATCTCCCATCTTGG
167    CTCCTGCCAAAGAAATTCAAGCGTCCTCACAAAGGGTTTAGTGCGCATCTCCCATCTTGG
       ************************************************** * *  *

172    GACATTTGCCCACTAAACATATCATATGACAGGAAGGAGAAAGATAGTAAGCAACAAATC
229    GACATTTGCCCACTAAACATATCATATGACAGGAAGGAGAAAGATAGTAAGCAACAAATC
208    GACATTTGCCCACTAAACATATCATATGACAGGAAGGAGAAAGATAGTAAGCAACAAATC
```

83	GACATTTGCCCACTAAACATATCATATGACAGGAAGGAGAAAGATAGTAAGCAACAAATC
3	GACATTTGCCCACTAAACATATCATATGACAGGAAGGAGAAAGATAGTAAGCAACAAATC
120	GACATTTGCCCACTAAACATATCATCTTCTTTGAAGGAGAAAGATATTAAGCAACAAATC
99	GACATTTGCCCACTAAACATAACATCTTCTTTGAAGGAGAAAGATAGTAAGCAACAAATC
126	GACATTTGCCCACTAAACATATCATATGACTGGTAGGAGAAAGATAGTAATCAACAAATC
173	GACATTTGCCCACTAAACATATCATATGACTGGTAGGAGAAAGATAGTAATCAACAAATC
204	GACATTTGCCCACTAAACATATCATATGACTGGAAGGAGAAAGATAGTAATCAACAAATC
239	CACATTTGCCCACTAAACATATCATATGACAGGTAGGAGAAAGATAGTATTCAACAAATC
117	CACATTTGCCCACTAAACATATCATATGACTGGTAGGAGAAAGATAGTAATCAACAAATC
123	CACATTTGCCCACTAAACATATCATATGACTGGTAGGAGAAAGATAGTATTCAACAAATC
210	GACATTTGCCCACTAAACATATCATATGACAGGAAGGAGAAAGATAGTATGCAACAAATC
86	GACATTTGCCCACTAAACATATCATATGACAGGAAGGAGAAAGATAGTATGCAACAAATC
100	GACATTTGCCCACTAAACATATCATATGACAGGAAGGAGAAAGATAGTATGCAACAAATC
114	GACATTTGCCCACTAAACATATCATATGACAGGAAGGAGAAAGATAGTATTCAACAAATC
170	GACATTTGCCCACTAAACATATCATATGACAGGAAGGAGAAAGATAGTATTCAACAAATC
108	GACATTTGCCCACTAAACATATCATATGACAGGAAGGAGAAAGATAGTATGCAACAAATC
174	GACATTTGCCCACTAAACATATCATATGACAGGAAGGAGAAAGATAGTATGCAACAAATC
158	GACATTTGCCCACTAAACATATCATATGACAGGAAGGAGAAAGATAGTAAGCAACAAATC
154	GACATTTGCCCACTAAACATATCATATGACAGGAAGGAGAAAGATAGTATGCAACAAATC
30	GACATTTGCCCACTAAACATATCATATGACAGGAAGGAGAAAGATAGTAAGCAACAAATC
39	GACATTTGCCCACTAAACATATCATATGACAGGAAGGAGAAAGATAGTAAGCAACAAATC
237	GACATTTGCCCACTAAACATATCATATGACAGGAAGGAGAAAGATAGTAAGCAACAAATC
65	GACATTTGCCCACTAAACATATCATATGACAGGAAGGAGAAAGATAGTAAGCAACAAATC
44	GACATTTGCCCACTAAACATATCATATGACAGGAAGGAGAAAGATAGTAAGCAACAAATC
168	GACATTTGCCCACTAAACATATCATATGACAGGAAGGAGAAAGATAGTAAGCAACAAATC
95	GACATTTGCCCACTAAACATAACATATGACAGGAAGGAGAAAGATATTAATCAACAAATC
22	GACATTTGCCCACTAAACATATCATATGACAGGAAGGAGAAAGATAGTAAGCAACAAATC
79	GACATTTGCCCACTAAACATATCATATGACAGGAAGGAGAAAGATAGTAAGCAACAAATC
211	GACATTTGCCCACTAAACATATCATATGACAGGAAGGAGAAAGATAGTAAGCAACAAATC
88	GACATTTGCCCACTAAACATATCATATGACAGGAAGGAGAAAGATAGTAAGCAACAAATC
184	GACATTTGCCCACTAAACATATCATATGACAGGAAGGAGAAAGATAGTAAGCAACAAATC
78	GACATTTGCCCACTAAACATATCATATGACAGGAAGGAGAAAGATAGTAAGCAACAAATC
71	GACATTTGCCCACTAAACATATCATATGACAGGAAGGAGAAAGATAGTAAGCAACAAATC
196	GACATTTGCCCACTAAACATATCATATGACAGGAAGGAGAAAGATAGTAAGCAACAAATC
43	GACATTTGCCCACTAAACATATCATATGACAGGAAGGAGAAAGATAGTAAGCAACAAATC
151	GACATTTGCCCACTAAACATATCATATGACAGGAAGGAGAAAGATAGTAAGCAACAAATC
27	GACATTTGCCCACTAAACATATCATATGACAGGAAGGAGAAAGATAGTATTCAACAAATC
197	GACATTTGCCCACTAAACATATCATATGACAGGAAGGAGAAAGATAGTAAGCAACAAATC
109	GACATTTGCCCACTAAACATATCATATGACAGGAAGGAGAAAGATAGTAAGCAACAAATC
169	GACATTTGCCCACTAAACATATCATATGACAGGAAGGAGAAAGATAGTAAGCAACAAATC
94	GACATTTGCCCACTAAACATATCATATGACAGGAAGGAGAAAGATAGTAAGCAACAAATC
232	GACATTTGCCCACTAAACATATCATATGACAGGAAGGAGAAAGATAGTAAGCAACAAATC
222	GACATTTGCCCACTAAACATATCATATGACAGGAAGGAGAAAGATAGTAAGCAACAAATC
24	GACATTTGCCCACTAAACATATCATATGACAGGAAGGAGAAAGATAGTAAGCAACAAATC
15	GACATTTGCCCACTAAACATATCATATGACAGGAAGGAGAAAGATAGTAAGCAACAAATC
13	GACATTTGCCCACTAAACATATCATATGACAGGAAGGAGAAAGATAGTAAGCAACAAATC
14	GACATTTGCCCACTAAACATATCATATGACAGGAAGGAGAAAGATAGTAAGCAACAAATC
35	GACATTTGCCCACTAAACATATCATATGACAGGAAGGAGAAAGATAGTAAGCAACAAATC
111	GACATTTGCCCACTAAACATATCATATGACAGGAAGGAGAAAGATAGTAAGCAACAAATC
157	GACATTTGCCCACTAAACATATCATATGACAGGAAGGAGAAAGATAGTAAGCAACAAATC
31	GACATTTGCCCACTAAACATATCATATGACAGGAAGGAGAAAGATAGTAAGCAACAAATC
47	GACATTTGCCCACTAAACATATCATATGACAGGAAGGAGAAAGATAGTAAGCAACAAATC
33	GACATTTGCCCACTAAACATATCATATGACAGGAAGGAGAAAGATAGTAAGCAACAAATC
51	GACATTTGCCCACTAAACATATCATATGACAGGAAGGAGAAAGATAGTAAGCAACAAATC
85	GACATTTGCCCACTAAACATATCATATGACAGGAAGGAGAAAGATAGTAAGCAACAAATC
54	GACATTTGCCCACTAAACATATCATATGACAGGAAGGAGAAAGATAGTAAGCAACAAATC
96	GACATTTGCCCACTAAACATATCATATGACAGGAAGGAGAAAGATAGTAAGCAACAAATC

61	GACATTTGCCCACTAAACATATCATATGACAGGAAGGAGAAAGATAGTAAGCAACAAATC
80	GACATTTGCCCACTAAACATATCATATGACAGGAAGGAGAAAGATAGTAAGCAACAAATC
82	GACATTTGCCCACTAAACATATCATATGACAGGAAGGAGAAAGATAGTAAGCAACAAATC
129	GACATTTGCCCACTAAACATATCATATGACAGGAAGGAGAAAGATAGTAAGCAACAAATC
46	GACATTTGCCCACTAAACATATCATATGACAGGAAGGAGAAAGATAGTAAGCAACAAATC
74	GACATTTGCCCACTAAACATATCATATGACAGGAAGGAGAAAGATAGTAAGCAACAAATC
101	GACATTTGCCCACTAAACATATCATATGACAGGAAGGAGAAAGATAGTAAGCAACAAATC
66	GACATTTGCCCACTAAACATATCATATGACAGGAAGGAGAAAGATAGTAAGCAACAAATC
104	GACATTTGCCCACTAAACATATCATATGACAGGAAGGAGAAAGATAGTAAGCAACAAATC
118	GACATTTGCCCACTAAACATATCATATGACAGGAAGGAGAAAGATAGTAAGCAACAAATC
21	GACATTTGCCCACTAAACATATCATATGACAGGTAGGAGAAAGATAGTAAGCAACAAATC
77	GACATTTGCCCACTAAACATATCATATGACAGGTAGGAGAAAGATAGTAAGCAACAAATC
124	GACATTTGCCCACTAAACATATCATATGACAGGTAGGAGAAAGATAGTAAGCAACAAATC
20	GACATTTGCCCACTAAACATATCATATGACAGGTAGGAGAAAGATAGTAAGCAACAAATC
110	GACATTTGCCCACTAAACATATCATATGACAGGTAGGAGAAAGATAGTAAGCAACAAATC
70	GACATTTGCCCACTAAACATATCATATGACAGGAAGGAGAAAGATAGTAAGCAACAAATC
8	GACATTTGCCCACTAAACATATCATATGACAGGAAGGAGAAAGATAGTAAGCAACAAATC
55	GACATTTGCCCACTAAACATATCATATGACAGGAAGGAGAAAGATAGTAAGCAACAAATC
64	GACATTTGCCCACTAAACATATCATATGACAGGAAGGAGAAAGATAGTAAGCAACAAATC
93	GACATTTGCCCACTAAACATATCATATGACAGGAAGGAGAAAGATAGTAAGCAACAAATC
202	GACATTTGCCCACTAAACATATCATATGACAGGAAGGAGAAAGATAGTAAGCAACAAATC
10	GACATTTGCCCACTAAACATATCATATGACAGGAAGGAGAAAGATAGTAAGCAACAAATC
16	GACATTTGCCCACTAAACATATCATATGACAGGAAGGAGAAAGATAGTAAGCAACAAATC
76	GACATTTGCCCACTAAACATATCATATGACAGGAAGGAGAAAGATAGTAAGCAACAAATC
116	GACATTTGCCCACTAAACATATCATATGACAGGAAGGAGAAAGATAGTAAGCAACAAATC
220	GACATTTGCCCACTAAACATATCATATGACAGGAAGGAGAAAGATAGTAAGCAACAAATC
234	GACATTTGCCCACTAAACATATCATATGACAGGAAGGAGAAAGATAGTAAGCAACAAATC
2	GACATTTGCCCACTAAACATATCATATGACAGGAAGGAGAAAGATAGTAAGCAACAAATC
38	GACATTTGCCCACTAAACATATCATATGACAGGAAGGAGAAAGATAGTAAGCAACAAATC
73	GACATTTGCCCACTAAACATATCATATGACAGGAAGGAGAAAGATAGTAAGCAACAAATC
87	GACATTTGCCCACTAAACATATCATATGACAGGAAGGAGAAAGATAGTAAGCAACAAATC
92	GACATTTGCCCACTAAACATATCATATGACAGGAAGGAGAAAGATAGTAAGCAACAAATC
98	GACATTTGCCCACTAAACATATCATATGACAGGAAGGAGAAAGATAGTAAGCAACAAATC
137	GACATTTGCCCACTAAACATATCATATGACAGGAAGGAGAAAGATAGTAAGCAACAAATC
19	GACATTTGCCCACTAAACATATCATATGACAGGAAGGAGAAAGATAGTAAGCAACAAATC
171	GACATTTGCCCACTAAACATATCATATGACAGGAAGGAGAAAGATAGTAAGCAACAAATC
205	GACATTTGCCCACTAAACATATCATATGACAGGAAGGAGAAAGATAGTAAGCAACAAATC
75	GACATTTGCCCACTAAACATATCATATGACAGGAAGGAGAAAGATAGTAAGCAACAAATC
62	GACATTTGCCCACTAAACATATCATATGACAGGAAGGAGAAAGATAGTAAGCAACAAATC
4	GACATTTGCCCACTAAACATATCATATGACAGGAAGGAGAAAGATAGTAAGCAACAAATC
7	GACATTTGCCCACTAAACATATCATATGACAGGAAGGAGAAAGATAGTAAGCAACAAATC
25	GACATTTGCCCACTAAACATATCATATGACAGGAAGGAGAAAGATAGTAAGCAACAAATC
28	GACATTTGCCCACTAAACATATCATATGACAGGAAGGAGAAAGATAGTAAGCAACAAATC
36	GACATTTGCCCACTAAACATATCATATGACAGGAAGGAGAAAGATAGTAAGCAACAAATC
49	GACATTTGCCCACTAAACATATCATATGACAGGAAGGAGAAAGATAGTAAGCAACAAATC
145	GACATTTGCCCACTAAACATATCATATGACAGGAAGGAGAAAGATAGTAAGCAACAAATC
181	GACATTTGCCCACTAAACATATCATATGACAGGAAGGAGAAAGATAGTAAGCAACAAATC
188	GACATTTGCCCACTAAACATATCATATGACAGGAAGGAGAAAGATAGTAAGCAACAAATC
59	GACATTTGCCCACTAAACATATCATATGACAGGAAGGAGAAAGATAGTAAGCAACAAATC
121	GACATTTGCCCACTAAACATATCATATGACAGGAAGGAGAAAGATAGTAAGCAACAAATC
127	GACATTTGCCCACTAAACATATCATATGACAGGAAGGAGAAAGATAGTAAGCAACAAATC
135	GACATTTGCCCACTAAACATATCATATGACAGGAAGGAGAAAGATAGTAAGCAACAAATC
136	GACATTTGCCCACTAAACATATCATATGACAGGAAGGAGAAAGATAGTAAGCAACAAATC
29	GACATTTGCCCACTAAACATATCATATGACAGGAAGGAGAAAGATAGTAAGCAACAAATC
89	GACATTTGCCCACTAAACATATCATATGACAGGAAGGAGAAAGATAGTAAGCAACAAATC
216	GACATTTGCCCACTAAACATATCATATGACAGGAAGGAGAAAGATAGTAAGCAACAAATC
164	GACATTTGCCCACTAAACATATCATATGACAGGAAGGAGAAAGATAGTAAGCAACAAATC

```
63              GACATTTGCCCACTAAACATATCATATGACAGGAAGGAGAAAGATAGTAAGCAACAAATC
161             GACATTTGCCCACTAAACATATCATATGACAGGAAGGAGAAAGATAGTAAGCAACAAATC
183             GACATTTGCCCACTAAACATATCATATGACAGGAAGGAGAAAGATAGTAAGCAACAAATC
18              GACATTTGCCCACTAAACATATCATATGACAGGAAGGAGAAAGATAGTAAGCAACAAATC
90              GACATTTGCCCACTAAACATATCATATGACAGGAAGGAGAAAGATAGTAAGCAACAAATC
162             GACATTTGCCCACTAAACATATCATATGACAGGAAGGAGAAAGATAGTAAGCAACAAATC
177             GACATTTGCCCACTAAACATATCATATGACAGGAAGGAGAAAGATAGTAAGCAACAAATC
97              GACATTTGCCCACTAAACATATCATATGACAGGAAGGAGAAAGATAGTAAGCAACAAATC
231             GACATTTGCCCACTAAACATATCATATGACAGGAAGGAGAAAGATAGTAAGCAACAAATC
143             GACATTTGCCCACTAAACATATCATATGACAGGAAGGAGAAAGATAGTAAGCAACAAATC
11              GACATTTGCCCACTAAACATATCATATGACAGGAAGGAGAAAGATAGTAAGCAACAAATC
153             GACATTTGCCCACTAAACATATCATATGACAGGAAGGAGAAAGATAGTAAGCAACAAATC
138             GACATTTGCCCACTAAACATATCATATGACAGGAAGGAGAAAGATAGTAAGCAACAAATC
185             GACATTTGCCCACTAAACATATCATATGACAGGAAGGAGAAAGATAGTAAGCAACAAATC
194             GACATTTGCCCACTAAACATATCATATGACAGGAAGGAGAAAGATAGTAAGCAACAAATC
236             GACATTTGCCCACTAAACATATCATATGACAGGAAGGAGAAAGATAGTAAGCAACAAATC
32              GACATTTGCCCACTAAACATATCATATGACAGGAAGGAGAAAGATAGTAAGCAACAAATC
52              GACATTTGCCCACTAAACATATCATATGACAGGAAGGAGAAAGATAGTAAGCAACAAATC
60              GACATTTGCCCACTAAACATATCATATGACAGGAAGGAGAAAGATAGTAAGCAACAAATC
72              GACATTTGCCCACTAAACATATCATATGACAGGAAGGAGAAAGATAGTAAGCAACAAATC
112             GACATTTGCCCACTAAACATATCATATGACAGGAAGGAGAAAGATAGTAAGCAACAAATC
141             GACATTTGCCCACTAAACATATCATATGACAGGAAGGAGAAAGATAGTAAGCAACAAATC
155             GACATTTGCCCACTAAACATATCATATGACAGGAAGGAGAAAGATAGTAAGCAACAAATC
1               GACATTTGCCCACTAAACATATCATATGACAGGAAGGAGAAAGATAGTAAGCAACAAATC
6               GACATTTGCCCACTAAACATATCATATGACAGGAAGGAGAAAGATAGTAAGCAACAAATC
58              GACATTTGCCCACTAAACATATCATATGACAGGAAGGAGAAAGATAGTAAGCAACAAATC
68              GACATTTGCCCACTAAACATATCATATGACAGGAAGGAGAAAGATAGTAAGCAACAAATC
180             GACATTTGCCCACTAAACATATCATATGACAGGAAGGAGAAAGATAGTAAGCAACAAATC
81              GACATTTGCCCACTAAACATATCATATGACAGGAAGGAGAAAGATAGTAAGCAACAAATC
103             GACATTTGCCCACTAAACATATCATATGACAGGAAGGAGAAAGATAGTAAGCAACAAATC
113             GACATTTGCCCACTAAACATATCATATGACAGGAAGGAGAAAGATAGTAAGCAACAAATC
163             GACATTTGCCCACTAAACATATCATATGACAGGAAGGAGAAAGATAGTAAGCAACAAATC
40              GACATTTGCCCACTAAACATATCATATGACAGGAAGGAGAAAGATAGTAAGCAACAAATC
56              GACATTTGCCCACTAAACATATCATATGACAGGAAGGAGAAAGATAGTAAGCAACAAATC
218             GACATTTGCCCACTAAACATATCATATGACAGGAAGGAGAAAGATAGTAAGCAACAAATC
41              GACATTTGCCCACTAAACATATCATATGACAGGAAGGAGAAAGATAGTAAGCAACAAATC
42              GACATTTGCCCACTAAACATATCATATGACAGGAAGGAGAAAGATAGTAAGCAACAAATC
160             GACATTTGCCCACTAAACATATCATATGACAGGAAGGAGAAAGATAGTAAGCAACAAATC
5               GACATTTGCCCACTAAACATATCATATGACAGGAAGGAGAAAGATAGTAAGCAACAAATC
228             GACATTTGCCCACTAAACATATCATATGACAGGAAGGAGAAAGATAGTAAGCAACAAATC
17              GACATTTGCCCACTAAACATATCATATGACAGGAAGGAGAAAGATAGTAAGCAACAAATC
37              GACATTTGCCCACTAAACATATCATATGACAGGAAGGAGAAAGATAGTAAGCAACAAATC
91              GACATTTGCCCACTAAACATATCATATGACAGGAAGGAGAAAGATAGTAAGCAACAAATC
140             GACATTTGCCCACTAAACATATCATATGACAGGAAGGAGAAAGATAGTATGCAACAAATC
167             GACATTTGCCCACTAAACATATCATATGACTGGAAGGAGAAAGATAGTATGCAACAAATC
                 ******************** *** *     * ************ **  *********

172             AGAACAAAGCAGCAGCCAACAAAAAAGGATATCAGTATATCACTAGATGCACCTGGTTGA
229             AGAACAAAGCAGCAGCCAACAAAAAAGGATATCAGTATATCACTAGATGCACCTGGTTGA
208             AGAACAAAGCAGCAGCCAACAAAAAAGGATATCAGTATATCACTAGATGCACCTGGTTGA
83              AGAACAAAGCAGCAGCCAACAAAAAAGGATATCAGTATATCACTAGATGCACCTGGTTGA
3               AGAACAAAGCAGCAGCCAACAAAAAAGGATATCAGTATATCACTAGATGCACCTGGTTGA
120             AGAACAAAGCAGCAGCCAACAAAAAAGGATATCAGTATATCACTAGTTGCACCTGGTTGA
99              AGAACAAAGCAGCAGCCAACAAAAAAGGATATCAGTATATCACTAGTTGCACCTGGTTGA
126             AGAACAAAGCAGCAGCCAACAAAAAAGGATATCAGTATATCACTAGTTGCACCTGGTTGA
173             AGAACAAAGCAGCAGCCAACAAAAAAGGATATCAGTATATCACTAGTTGCACCTGGTTGA
204             AGAACAAAGCAGCAGCCAACAAAAAAGGATATCAGTATATCACTAGTTGCACCTGGTTGA
```

239	AGAACAAAGCAGCAGCCAACAAAAAAGGATATCAGTATATCACTAGTTGCACCTGGTTGA
117	AGAACAAAGCAGCAGCCAACAAAAAAGGATATCAGTATATCACTAGTTGCACCTGGTTGA
123	AGAACAAAGCAGCAGCCAACAAAAAAGGATATCAGTATATCACTAGTTGCACCTGGTTGA
210	AGAACAAAGCAGCAGCCAACAAAAAAGGATATCAGTATATCACTAGTTGCACCTGGTTGA
86	AGAACAAAGCAGCAGCCAACAAAAAAGGATATCAGTATATCACTAGTTGCACCTGGTTGA
100	AGAACAAAGCAGCAGCCAACAAAAAAGGATATCAGTATATCACTAGTTGCACCTGGTTGA
114	AGAACAAAGCAGCAGCCAACAAAAAAGGATATCAGTATATCACTAGTTGCACCTGGTTGA
170	AGAACAAAGCAGCAGCCAACAAAAAAGGATATCAGTATATCACTAGTTGCACCTGGTTGA
108	AGAACAAAGCAGCAGCCAACAAAAAAGGATATCAGTATATCACTAGTTGCACCTGGTTGA
174	AGAACAAAGCAGCAGCCAACAAAAAAGGATATCAGTATATCACTAGTTGCACCTGGTTGA
158	AGAACAAAGCAGCAGCCAACAAAAAAGGATATCAGTATATCACTAGTTGCACCTGGTTGA
154	AGAACAAAGCAGCAGCCAACAAAAAAGGATATCAGTATATCACTAGTTGCACCTGGTTGA
30	AGAACAAAGCAGCAGCCAACAAAAAAGGATATCAGTATATCACTAGTTGCACCTGGTTGA
39	AGAACAAAGCAGCAGCCAACAAAAAAGGATATCAGTATATCACTAGTTGCACCTGGTTGA
237	AGAACAAAGCAGCAGCCAACAAAAAAGGATATCAGTATATCACTAGTTGCACCTGGTTGA
65	AGAACAAAGCAGCAGCCAACAAAAAAGGATATCAGTATATCACTAGTTGCACCTGGTTGA
44	AGAACAAAGCAGCAGCCAACAAAAAAGGATATCAGTATATCACTAGTTGCACCTGGTTGA
168	AGAACAAAGCAGCAGCCAACAAAAAAGGATATCAGTATATAACTAGTTGCACCTGGTTGA
95	AGAACAAAGCAGCAGCCAACAAAAAAGGATATCAGTATATCACTAGTTGCACCTGGTTGA
22	AGAACAAAGCAGCAGCCAACAAAAAAGGATATCAGTATATCACTAGTTGCACCTGGTTGA
79	AGAACAAAGCAGCAGCCAACAAAAAAGGATATCAGTATATCACTAGTTGCACCTGGTTGA
211	AGAACAAAGCAGCAGCCAACAAAAAAGGATATCAGTATATCACTAGTTGCACCTGGTTGA
88	AGAACAAAGCAGCAGCCAACAAAAAAGGATATCAGTATATCACTAGTTGCACCTGGTTGA
184	AGAACAAAGCAGCAGCCAACAAAAAAGGATATCAGTATATCACTAGTTGCACCTGGTTGA
78	AGAACAAAGCAGCAGCCAACAAAAAAGGATATCAGTATATCACTAGTTGCACCTGGTTGA
71	AGAACAAAGCAGCAGCCAACAAAAAAGGATATCAGTATATCACTAGTTGCACCTGGTTGA
196	AGAACAAAGCAGCAGCCAACAAAAAAGGATATCAGTATATCACTAGTTGCACCTGGTTGA
43	AGAACAAAGCAGCAGCCAACAAAAAAGGATATCAGTATATCACTAGTTGCACCTGGTTGA
151	AGAACAAAGCAGCAGCCAACAAAAAAGGATATCAGTATATCACTAGTTGCACCTGGTTGA
27	AGAACAAAGCAGCAGCCAACAAAAAAGGATATCAGTATATCACTAGTTGCACCTGGTTGA
197	AGAACAAAGCAGCAGCCAACAAAAAAGGATATCAGTATATCACTAGTTGCACCTGGTTGA
109	AGAACAAAGCAGCAGCCAACAAAAAAGGATATCAGTATATCACTAGTTGCACCTGGTTGA
169	AGAACAAAGCAGCAGCCAACAAAAAAGGATATCAGTATATCACTAGTTGCACCTGGTTGA
94	AGAACAAAGCAGCAGCCAACAAAAAAGGATATCAGTATATCACTAGTTGCACCTGGTTGA
232	AGAACAAAGCAGCAGCCAACAAAAAAGGATATCAGTATATCACTAGTTGCACCTGGTTGA
222	AGAACAAAGCAGCAGCCAACAAAAAAGGATATCAGTATATCACTAGTTGCACCTGGTTGA
24	AGAACAAAGCAGCAGCCAACAAAAAAGGATATCAGTATATCACTAGTTGCACCTGGTTGA
15	AGAACAAAGCAGCAGCCAACAAAAAAGGATATCAGTATATCACTAGTTGCACCTGGTTGA
13	AGAACAAAGCAGCAGCCAACAAAAAAGGATATCAGTATATCACTAGTTGCACCTGGTTGA
14	AGAACAAAGCAGCAGCCAACAAAAAAGGATATCAGTATATCACTAGTTGCACCTGGTTGA
35	AGAACAAAGCAGCAGCCAACAAAAAAGGATATCAGTATATCACTAGTTGCACCTGGTTGA
111	AGAACAAAGCAGCAGCCAACAAAAAAGGATATCAGTATATCACTAGTTGCACCTGGTTGA
157	AGAACAAAGCAGCAGCCAACAAAAAAGGATATCAGTATATCACTAGTTGCACCTGGTTGA
31	AGAACAAAGCAGCAGCCAACAAAAAAGGATATCAGTATATCACTAGTTGCACCTGGTTGA
47	AGAACAAAGCAGCAGCCAACAAAAAAGGATATCAGTATATCACTAGTTGCACCTGGTTGA
33	AGAACAAAGCAGCAGCCAACAAAAAAGGATATCAGTATATCACTAGTTGCACCTGGTTGA
51	AGAACAAAGCAGCAGCCAACAAAAAAGGATATCAGTATATCACTAGTTGCACCTGGTTGA
85	AGAACAAAGCAGCAGCCAACAAAAAAGGATATCAGTATATCACTAGTTGCACCTGGTTGA
54	AGAACAAAGCAGCAGCCAACAAAAAAGGATATCAGTATATCACTAGTTGCACCTGGTTGA
96	AGAACAAAGCAGCAGCCAACAAAAAAGGATATCAGTATATCACTAGTTGCACCTGGTTGA
61	AGAACAAAGCAGCAGCCAACAAAAAAGGATATCAGTATATCACTAGTTGCACCTGGTTGA
80	AGAACAAAGCAGCAGCCAACAAAAAAGGATATCAGTATATCACTAGTTGCACCTGGTTGA
82	AGAACAAAGCAGCAGCCAACAAAAAAGGATATCAGTATATCACTAGTTGCACCTGGTTGA
129	AGAACAAAGCAGCAGCCAACAAAAAAGGATATCAGTATATCACTAGTTGCACCTGGTTGA
46	AGAACAAAGCAGCAGCCAACAAAAAAGGATATCAGTATATCACTAGTTGCACCTGGTTGA
74	AGAACAAAGCAGCAGCCAACAAAAAAGGATATCAGTATATCACTAGTTGCACCTGGTTGA
101	AGAACAAAGCAGCAGCCAACAAAAAAGGATATCAGTATATCACTAGTTGCACCTGGTTGA

66	AGAACAAAGCAGCAGCCAACAAAAAAGGATATCAGTATATCACTAGTTGCACCTGGTTGA
104	AGAACAAAGCAGCAGCCAACAAAAAAGGATATCAGTATATCACTAGTTGCACCTGGTTGA
118	AGAACAAAGCAGCAGCCAACAAAAAAGGATATCAGTATATCACTAGTTGCACCTGGTTGA
21	AGAACAAAGCAGCAGCCAACAAAAAAGGATATCAGTATATCACTAGTTGCACCTGGTTGA
77	AGAACAAAGCAGCAGCCAACAAAAAAGGATATCAGTATATCACTAGTTGCACCTGGTTGA
124	AGAACAAAGCAGCAGCCAACAAAAAAGGATATCAGTATATCACTAGTTGCACCTGGTTGA
20	AGAACAAAGCAGCAGCCAACAAAAAAGGATATCAGTATATCACTAGTTGCACCTGGTTGA
110	AGAACAAAGCAGCAGCCAACAAAAAAGGATATCAGTATATCACTAGTTGCACCTGGTTGA
70	AGAACAAAGCAGCAGCCAACAAAAAAGGATATCAGTATATCACTAGTTGCACCTGGTTGA
8	AGAACAAAGCAGCAGCCAACAAAAAAGGATATCAGTATATCACTAGTTGCACCTGGTTGA
55	AGAACAAAGCAGCAGCCAACAAAAAAGGATATCAGTATATCACTAGTTGCACCTGGTTGA
64	AGAACAAAGCAGCAGCCAACAAAAAAGGATATCAGTATATCACTAGTTGCACCTGGTTGA
93	AGAACAAAGCAGCAGCCAACAAAAAAGGATATCAGTATATCACTAGTTGCACCTGGTTGA
202	AGAACAAAGCAGCAGCCAACAAAAAAGGATATCAGTATATCACTAGTTGCACCTGGTTGA
10	AGAACAAAGCAGCAGCCAACAAAAAAGGATATCAGTATATCACTAGTTGCACCTGGTTGA
16	AGAACAAAGCAGCAGCCAACAAAAAAGGATATCAGTATATCACTAGTTGCACCTGGTTGA
76	AGAACAAAGCAGCAGCCAACAAAAAAGGATATCAGTATATCACTAGTTGCACCTGGTTGA
116	AGAACAAAGCAGCAGCCAACAAAAAAGGATATCAGTATATCACTAGTTGCACCTGGTTGA
220	AGAACAAAGCAGCAGCCAACAAAAAAGGATATCAGTATATCACTAGTTGCACCTGGTTGA
234	AGAACAAAGCAGCAGCCAACAAAAAAGGATATCAGTATATCACTAGTTGCACCTGGTTGA
2	AGAACAAAGCAGCAGCCAACAAAAAAGGATATCAGTATATCACTAGTTGCACCTGGTTGA
38	AGAACAAAGCAGCAGCCAACAAAAAAGGATATCAGTATATCACTAGTTGCACCTGGTTGA
73	AGAACAAAGCAGCAGCCAACAAAAAAGGATATCAGTATATCACTAGTTGCACCTGGTTGA
87	AGAACAAAGCAGCAGCCAACAAAAAAGGATATCAGTATATCACTAGTTGCACCTGGTTGA
92	AGAACAAAGCAGCAGCCAACAAAAAAGGATATCAGTATATCACTAGTTGCACCTGGTTGA
98	AGAACAAAGCAGCAGCCAACAAAAAAGGATATCAGTATATCACTAGTTGCACCTGGTTGA
137	AGAACAAAGCAGCAGCCAACAAAAAAGGATATCAGTATATCACTAGTTGCACCTGGTTGA
19	AGAACAAAGCAGCAGCCAACAAAAAAGGATATCAGTATATCACTAGTTGCACCTGGTTGA
171	AGAACAAAGCAGCAGCCAACAAAAAAGGATATCAGTATATCACTAGTTGCACCTGGTTGA
205	AGAACAAAGCAGCAGCCAACAAAAAAGGATATCAGTATATCACTAGTTGCACCTGGTTGA
75	AGAACAAAGCAGCAGCCAACAAAAAAGGATATCAGTATATCACTAGTTGCACCTGGTTGA
62	AGAACAAAGCAGCAGCCAACAAAAAAGGATATCAGTATATCACTAGTTGCACCTGGTTGA
4	AGAACAAAGCAGCAGCCAACAAAAAAGGATATCAGTATATCACTAGTTGCACCTGGTTGA
7	AGAACAAAGCAGCAGCCAACAAAAAAGGATATCAGTATATCACTAGTTGCACCTGGTTGA
25	AGAACAAAGCAGCAGCCAACAAAAAAGGATATCAGTATATCACTAGTTGCACCTGGTTGA
28	AGAACAAAGCAGCAGCCAACAAAAAAGGATATCAGTATATCACTAGTTGCACCTGGTTGA
36	AGAACAAAGCAGCAGCCAACAAAAAAGGATATCAGTATATCACTAGTTGCACCTGGTTGA
49	AGAACAAAGCAGCAGCCAACAAAAAAGGATATCAGTATATCACTAGTTGCACCTGGTTGA
145	AGAACAAAGCAGCAGCCAACAAAAAAGGATATCAGTATATCACTAGTTGCACCTGGTTGA
181	AGAACAAAGCAGCAGCCAACAAAAAAGGATATCAGTATATCACTAGTTGCACCTGGTTGA
188	AGAACAAAGCAGCAGCCAACAAAAAAGGATATCAGTATATCACTAGTTGCACCTGGTTGA
59	AGAACAAAGCAGCAGCCAACAAAAAAGGATATCAGTATATCACTAGTTGCACCTGGTTGA
121	AGAACAAAGCAGCAGCCAACAAAAAAGGATATCAGTATATCACTAGTTGCACCTGGTTGA
127	AGAACAAAGCAGCAGCCAACAAAAAAGGATATCAGTATATCACTAGTTGCACCTGGTTGA
135	AGAACAAAGCAGCAGCCAACAAAAAAGGATATCAGTATATCACTAGTTGCACCTGGTTGA
136	AGAACAAAGCAGCAGCCAACAAAAAAGGATATCAGTATATCACTAGTTGCACCTGGTTGA
29	AGAACAAAGCAGCAGCCAACAAAAAAGGATATCAGTATATCACTAGTTGCACCTGGTTGA
89	AGAACAAAGCAGCAGCCAACAAAAAAGGATATCAGTATATCACTAGTTGCACCTGGTTGA
216	AGAACAAAGCAGCAGCCAACAAAAAAGGATATCAGTATATCACTAGTTGCACCTGGTTGA
164	AGAACAAAGCAGCAGCCAACAAAAAAGGATATCAGTATATCACTAGTTGCACCTGGTTGA
63	AGAACAAAGCAGCAGCCAACAAAAAAGGATATCAGTATATCACTAGTTGCACCTGGTTGA
161	AGAACAAAGCAGCAGCCAACAAAAAAGGATATCAGTATATCACTAGTTGCACCTGGTTGA
183	AGAACAAAGCAGCAGCCAACAAAAAAGGATATCAGTATATCACTAGTTGCACCTGGTTGA
18	AGAACAAAGCAGCAGCCAACAAAAAAGGATATCAGTATATCACTAGTTGCACCTGGTTGA
90	AGAACAAAGCAGCAGCCAACAAAAAAGGATATCAGTATATCACTAGTTGCACCTGGTTGA
162	AGAACAAAGCAGCAGCCAACAAAAAAGGATATCAGTATATCACTAGTTGCACCTGGTTGA
177	AGAACAAAGCAGCAGCCAACAAAAAAGGATATCAGTATATCACTAGTTGCACCTGGTTGA

```
97              AGAACAAAGCAGCAGCCAACAAAAAAGGATATCAGTATATCACTAGTTGCACCTGGTTGA
231             AGAACAAAGCAGCAGCCAACAAAAAAGGATATCAGTATATCACTAGTTGCACCTGGTTGA
143             AGAACAAAGCAGCAGCCAACAAAAAAGGATATCAGTATATAACTAGTTGCACCTGGTTGA
11              AGAACAAAGCAGCAGCCAACAAAAAAGGATATCAGTATATAACTAGTTGCACCTGGTTGA
153             AGAACAAAGCAGCAGCCAACAAAAAAGGATATCAGTATATAACTAGTTGCACCTGGTTGA
138             AGAACAAAGCAGCAGCCAACAAAAAAGGATATCAGTATATAACTAGTTGCACCTGGTTGA
185             AGAACAAAGCAGCAGCCAACAAAAAAGGATATCAGTATATAACTAGTTGCACCTGGTTGA
194             AGAACAAAGCAGCAGCCAACAAAAAAGGATATCAGTATATAACTAGTTGCACCTGGTTGA
236             AGAACAAAGCAGCAGCCAACAAAAAAGGATATCAGTATATAACTAGTTGCACCTGGTTGA
32              AGAACAAAGCAGCAGCCAACAAAAAAGGATATCAGTATATAACTAGTTGCACCTGGTTGA
52              AGAACAAAGCAGCAGCCAACAAAAAAGGATATCAGTATATAACTAGTTGCACCTGGTTGA
60              AGAACAAAGCAGCAGCCAACAAAAAAGGATATCAGTATATAACTAGTTGCACCTGGTTGA
72              AGAACAAAGCAGCAGCCAACAAAAAAGGATATCAGTATATAACTAGTTGCACCTGGTTGA
112             AGAACAAAGCAGCAGCCAACAAAAAAGGATATCAGTATATAACTAGTTGCACCTGGTTGA
141             AGAACAAAGCAGCAGCCAACAAAAAAGGATATCAGTATATAACTAGTTGCACCTGGTTGA
155             AGAACAAAGCAGCAGCCAACAAAAAAGGATATCAGTATATAACTAGTTGCACCTGGTTGA
1               AGAACAAAGCAGCAGCCAACAAAAAAGGATATCAGTATATAACTAGTTGCACCTGGTTGA
6               AGAACAAAGCAGCAGCCAACAAAAAAGGATATCAGTATATAACTAGTTGCACCTGGTTGA
58              AGAACAAAGCAGCAGCCAACAAAAAAGGATATCAGTATATAACTAGTTGCACCTGGTTGA
68              AGAACAAAGCAGCAGCCAACAAAAAAGGATATCAGTATATAACTAGTTGCACCTGGTTGA
180             AGAACAAAGCAGCAGCCAACAAAAAAGGATATCAGTATATAACTAGTTGCACCTGGTTGA
81              AGAACAAAGCAGCAGCCAACAAAAAAGGATATCAGTATATAACTAGTTGCACCTGGTTGA
103             AGAACAAAGCAGCAGCCAACAAAAAAGGATATCAGTATATAACTAGTTGCACCTGGTTGA
113             AGAACAAAGCAGCAGCCAACAAAAAAGGATATCAGTATATAACTAGTTGCACCTGGTTGA
163             AGAACAAAGCAGCAGCCAACAAAAAAGGATATCAGTATATAACTAGTTGCACCTGGTTGA
40              AGAACAAAGCAGCAGCCAACAAAAAAGGATATCAGTATATAACTAGTTGCACCTGGTTGA
56              AGAACAAAGCAGCAGCCAACAAAAAAGGATATCAGTATATCACTAGTTGCACCTGGTTGA
218             AGAACAAAGCAGCAGCCAACAAAAAAGGATATCAGTATATCACTAGTTGCACCTGGTTGA
41              AGAACAAAGCAGCAGCCAACAAAAAAGGATATCAGTATATCACTAGTTGCACCTGGTTGA
42              AGAACAAAGCAGCAGCCAACAAAAAAGGATATCAGTATATCACTAGTTGCACCTGGTTGA
160             AGAACAAAGCAGCAGCCAACAAAAAAGGATATCAGTATATCACTAGTTGCACCTGGTTGA
5               AGAACAAAGCAGCAGCCAACAAAAAAGGATATCAGTATATCACTAGTTGCACCTGGTTGA
228             AGAACAAAGCAGCAGCCAACAAAAAAGGATATCAGTATATCACTAGTTGCACCTGGTTGA
17              AGAACAAAGCAGCAGCCAACAAAAAAGGATATCAGTATATCACTAGTTGCACCTGGTTGA
37              AGAACAAAGCAGCAGCCAACAAAAAAGGATATCAGTATATCACTAGTTGCACCTGGTTGA
91              AGAACAAAGCAGCAGCCAACAAAAAAGGATATCAGTATATCACTAGTTGCACCTGGTTGA
140             AGAACAAAGCAGCAGCCAACAAAAAAGGATATCAGTATATCACTAGTTGCACCTGGTTGA
167             AGAACAAAGCAGCAGCCAACAAAAAAGGATATCAGTATATCACTAGTTGCACCTGGTTGA
                **************************************** ***** *************

172             TCATTAGAGGAAGATCACGATAACTATGAATCCACTGAGGAAACATGTGATTTACAATGG
229             TCATTAGAGGAAGATCACGATAACTATGAATCCACTGAGGAAACATGTGATTTACAATGG
208             TCATTAGAGGAAGATCACGATAACTATGAATCCACTGAGGAAACATGTGATTTACAATGG
83              TCATTAGAGGAAGATCACGATAACTATGAATCCACTGAGTAAACATGTGATTTACAATGG
3               TCATTAGAGGAAGATCACGATAACTATGAATCCACTGAGTAAACATGTGATTTACAATGG
120             TCATTAGAGGAAGATCACGATAACTATGAATCCACTGAGTAAACATGTGATTTACAATGG
99              TCATTAGAGGAAGATCACGATAACTATGAATCCACTGAGTAAACATGTGATTTACAATGG
126             TCATTAGAGGAAGATCACGATAACTATGAATCCGCTGAGGAAACATGTGATTTACAATGG
173             TCATTAGAGGAAGATCACGATAACTATGAATCCGCTGAGGAAACATGTGATTTACAATGG
204             TCATTAGAGGAAGATCACGATAACTATGAATCCACTGAGTAAACATGTGATTTACAATGG
239             TCATTAGAGGAAGATCACGATAACTATGAATCCACTGAGTAAACATGTGATTTACAATGG
117             TCATTAGAGGAAGATCACGATAACTATGAATCCACTGAGTAAACATGTGATTTACAATGG
123             TCATTAGAGGAAGATCACGATAACTATGAATCCACTGAGTAAACATGTGATTTACAATGG
210             TCATTAGAGGAAGATCACGATAACTATGAATCCGCTGAGGAAACATGTGATTTACAATGG
86              TCATTAGAGGAAGATCACGATAACTATGAATCCGCTGAGGAAACATGTGATTTACAATGG
100             TCATTAGAGGAAGATCACGATAACTATGAATCCGCTGAGGAAACATGTGATTTACAATGG
114             TCATTAGAGGAAGATCACGATAACTATGAATCCACTGAGTAAACATGTGATTTACAATGG
```

170	TCATTAGAGGAAGATCACGATAACTATGAATCCACTGAGTAAACATGTGATTTACAATGG
108	TCATTAGAGGAAGATCACGATAACTATGAATCCGCTGAGGAAACATGTGATTTACAATGG
174	TCATTAGAGGAAGATCACGATAACTATGAATCCGCTGAGGAAACATGTGATTTACAATGG
158	TCATTAGAGGAAGATCACGATAACTATGAATCCACTGAGTAAACATGTGATTTACAATGG
154	TCATTAGAGGAAGATCACGATAACTATGAATCCACTGAGTAAACATGTGATTTACAATGG
30	TCATTAGAGGAAGATCACGATAACTATGAATCCGCTGAGTAAACATGTGATTTACAATGG
39	TCATTAGAGGAAGATCACGATAACTATGAATCCGCTGAGTAAACATGTGATTTACAATGG
237	TCATTAGAGGAAGATCACGATAACTATGAATCCACTGAGTAAACATGTGATTTACAATGG
65	TCATTAGAGGAAGATCACGATAACTATGAATCCACTGAGTAAACATGTGATTTACAATGG
44	TCATTAGAGGAAGATCACGATAACTATGAATCCACTGAGTAAACATGTGATTTACAATGG
168	TCATTAGAGGAAGATCACGATAACTATGAATCCACTGAGTAAACATGTGATTTACAATGG
95	TCATTAGAGGAAGATCACGATAACTATGAATCCACTGAGTAAACATGTGATTTACAATGG
22	TCATTAGAGGAAGATCACGATAACTATGAATCCACTGAGTAAACATGTGATTTACAATGG
79	TCATTAGAGGAAGATCACGATAACTATGAATCCACTGAGTAAACATGTGATTTACAATGG
211	TCATTAGAGGAAGATCACGATAACTATGAATCCACTGAGTAAACATGTGATTTACAATGG
88	TCATTAGAGGAAGATCACGATAACTATGAATCCACTGAGTAAACATGTGATTTACAATGG
184	TCATTAGAGGAAGATCACGATAACTATGAATCCACTGAGTAAACATGTGATTTACAATGG
78	TCATTAGAGGAAGATCACGATAACTATGAATCCACTGAGTAAACATGTGATTTACAATGG
71	TCATTAGAGGAAGATCACGATAACTATGAATCCACTGAGGAAACATGTGATTTACAATGG
196	TCATTAGAGGAAGATCACGATAACTATGAATCCACTGAGGAAACATGTGATTTACAATGG
43	TCATTAGAGGAAGATCACGATAACTATGAATCCACTGAGTAAACATGTGATTTACAATGG
151	TCATTAGAGGAAGATCACGATAACTATGAATCCACTGAGTAAACATGTGATTTACAATGG
27	TCATTAGAGGAAGATCACGATAACTATGAATCCACTGAGTAAACATGTGATTTACAATGG
197	TCATTAGAGGAAGATCACGATAACTATGAATCCACTGAGTAAACATGTGATTTACAATGG
109	TCATTAGAGGAAGATCACGATAACTATGAATCCACTGAGTAAACATGTGATTTACAATGG
169	TCATTAGAGGAAGATCACGATAACTATGAATCCACTGAGTAAACATGTGATTTACAATGG
94	TCATTAGAGGAAGATCACGATAACTATGAATCCACTGAGTAAACATGTGATTTACAATGG
232	TCATTAGAGGAAGATCACGATAACTATGAATCCACTGAGTAAACATGTGATTTACAATGG
222	TCATTAGAGGAAGATCACGATAACTATGAATCCACTGAGTAAACATGTGATTTACAATGG
24	TCATTAGAGGAAGATCACGATAACTATGAATCCACTGAGTAAACATGTGATTTACAATGG
15	TCATTAGAGGAAGATCACGATAACTATGAATCCACTGAGTAAACATGTGATTTACAATGG
13	TCATTAGAGGAAGATCACGATAACTATGAATCCACTGAGTAAACATGTGATTTACAATGG
14	TCATTAGAGGAAGATCACGATAACTATGAATCCACTGAGTAAACATGTGATTTACAATGG
35	TCATTAGAGGAAGATCACGATAACTATGAATCCACTGAGTAAACATGTGATTTACAATGG
111	TCATTAGAGGAAGATCACGATAACTATGAATCCACTGAGTAAACATGTGATTTACAATGG
157	TCATTAGAGGAAGATCACGATAACTATGAATCCACTGAGTAAACATGTGATTTACAATGG
31	TCATTAGAGGAAGATCACGATAACTATGAATCCACTGAGTAAACATGTGATTTACAATGG
47	TCATTAGAGGAAGATCACGATAACTATGAATCCACTGAGTAAACATGTGATTTACAATGG
33	TCATTAGAGGAAGATCACGATAACTATGAATCCACTGAGTAAACATGTGATTTACAATGG
51	TCATTAGAGGAAGATCACGATAACTATGAATCCACTGAGTAAACATGTGATTTACAATGG
85	TCATTAGAGGAAGATCACGATAACTATGAATCCACTGAGTAAACATGTGATTTACAATGG
54	TCATTAGAGGAAGATCACGATAACTATGAATCCACTGAGTAAACATGTGATTTACAATGG
96	TCATTAGAGGAAGATCACGATAACTATGAATCCACTGAGTAAACATGTGATTTACAATGG
61	TCATTAGAGGAAGATCACGATAACTATGAATCCACTGAGTAAACATGTGATTTACAATGG
80	TCATTAGAGGAAGATCACGATAACTATGAATCCACTGAGTAAACATGTGATTTACAATGG
82	TCATTAGAGGAAGATCACGATAACTATGAATCCACTGAGTAAACATGTGATTTACAATGG
129	TCATTAGAGGAAGATCACGATAACTATGAATCCACTGAGTAAACATGTGATTTACAATGG
46	TCATTAGAGGAAGATCACGATAACTATGAATCCACTGAGTAAACATGTGATTTACAATGG
74	TCATTAGAGGAAGATCACGATAACTATGAATCCACTGAGTAAACATGTGATTTACAATGG
101	TCATTAGAGGAAGATCACGATAACTATGAATCCACTGAGTAAACATGTGATTTACAATGG
66	TCATTAGAGGAAGATCACGATAACTATGAATCCACTGAGTAAACATGTGATTTACAATGG
104	TCATTAGAGGAAGATCACGATAACTATGAATCCACTGAGTAAACATGTGATTTACAATGG
118	TCATTAGAGGAAGATCACGATAACTATGAATCCACTGAGTAAACATGTGATTTACAATGG
21	TCATTAGAGGAAGATCACGATAACTATGAATCCACTGAGTAAACATGTGATTTACAATGG
77	TCATTAGAGGAAGATCACGATAACTATGAATCCACTGAGTAAACATGTGATTTACAATGG
124	TCATTAGAGGAAGATCACGATAACTATGAATCCACTGAGTAAACATGTGATTTACAATGG
20	TCATTAGAGGAAGATCACGATAACTATGAATCCACTGAGTAAACATGTGATTTACAATGG

110	TCATTAGAGGAAGATCACGATAACTATGAATCCACTGAGTAAACATGTGATTTACAATGG
70	TCATTAGAGGAAGATCACGATAACTATGAATCCACTGAGTAAACATGTGATTTACAATGG
8	TCATTAGAGGAAGATCACGATAACTATGAATCCACTGAGTAAACATGTGATTTACAATGG
55	TCATTAGAGGAAGATCACGATAACTATGAATCCACTGAGTAAACATGTGATTTACAATGG
64	TCATTAGAGGAAGATCACGATAACTATGAATCCACTGAGTAAACATGTGATTTACAATGG
93	TCATTAGAGGAAGATCACGATAACTATGAATCCACTGAGTAAACATGTGATTTACAATGG
202	TCATTAGAGGAAGATCACGATAACTATGAATCCACTGAGTAAACATGTGATTTACAATGG
10	TCATTAGAGGAAGATCACGATAACTATGAATCCACTGAGTAAACATGTGATTTACAATGG
16	TCATTAGAGGAAGATCACGATAACTATGAATCCACTGAGTAAACATGTGATTTACAATGG
76	TCATTAGAGGAAGATCACGATAACTATGAATCCACTGAGTAAACATGTGATTTACAATGG
116	TCATTAGAGGAAGATCACGATAACTATGAATCCACTGAGTAAACATGTGATTTACAATGG
220	TCATTAGAGGAAGATCACGATAACTATGAATCCACTGAGTAAACATGTGATTTACAATGG
234	TCATTAGAGGAAGATCACGATAACTATGAATCCACTGAGTAAACATGTGATTTACAATGG
2	TCATTAGAGGAAGATCACGATAACTATGAATCCACTGAGTAAACATGTGATTTACAATGG
38	TCATTAGAGGAAGATCACGATAACTATGAATCCACTGAGTAAACATGTGATTTACAATGG
73	TCATTAGAGGAAGATCACGATAACTATGAATCCACTGAGTAAACATGTGATTTACAATGG
87	TCATTAGAGGAAGATCACGATAACTATGAATCCACTGAGTAAACATGTGATTTACAATGG
92	TCATTAGAGGAAGATCACGATAACTATGAATCCACTGAGTAAACATGTGATTTACAATGG
98	TCATTAGAGGAAGATCACGATAACTATGAATCCACTGAGTAAACATGTGATTTACAATGG
137	TCATTAGAGGAAGATCACGATAACTATGAATCCACTGAGTAAACATGTGATTTACAATGG
19	TCATTAGAGGAAGATCACGATAACTATGAATCCACTGAGTAAACATGTGATTTACAATGG
171	TCATTAGAGGAAGATCACGATAACTATGAATCCACTGAGTAAACATGTGATTTACAATGG
205	TCATTAGAGGAAGATCACGATAACTATGAATCCACTGAGTAAACATGTGATTTACAATGG
75	TCATTAGAGGAAGATCACGATAACTATGAATCCACTGAGTAAACATGTGATTTACAATGG
62	TCATTAGAGGAAGATCACGATAACTATGAATCCACTGAGTAAACATGTGATTTACAATGG
4	TCATTAGAGGAAGATCACGATAACTATGAATCCACTGAGTAAACATGTGATTTACAATGG
7	TCATTAGAGGAAGATCACGATAACTATGAATCCACTGAGTAAACATGTGATTTACAATGG
25	TCATTAGAGGAAGATCACGATAACTATGAATCCACTGAGTAAACATGTGATTTACAATGG
28	TCATTAGAGGAAGATCACGATAACTATGAATCCACTGAGTAAACATGTGATTTACAATGG
36	TCATTAGAGGAAGATCACGATAACTATGAATCCACTGAGTAAACATGTGATTTACAATGG
49	TCATTAGAGGAAGATCACGATAACTATGAATCCACTGAGTAAACATGTGATTTACAATGG
145	TCATTAGAGGAAGATCACGATAACTATGAATCCACTGAGTAAACATGTGATTTACAATGG
181	TCATTAGAGGAAGATCACGATAACTATGAATCCACTGAGTAAACATGTGATTTACAATGG
188	TCATTAGAGGAAGATCACGATAACTATGAATCCACTGAGTAAACATGTGATTTACAATGG
59	TCATTAGAGGAAGATCACGATAACTATGAATCCACTGAGTAAACATGTGATTTACAATGG
121	TCATTAGAGGAAGATCACGATAACTATGAATCCACTGAGTAAACATGTGATTTACAATGG
127	TCATTAGAGGAAGATCACGATAACTATGAATCCACTGAGTAAACATGTGATTTACAATGG
135	TCATTAGAGGAAGATCACGATAACTATGAATCCACTGAGTAAACATGTGATTTACAATGG
136	TCATTAGAGGAAGATCACGATAACTATGAATCCACTGAGTAAACATGTGATTTACAATGG
29	TCATTAGAGGAAGATCACGATAACTATGAATCCACTGAGTAAACATGTGATTTACAATGG
89	TCATTAGAGGAAGATCACGATAACTATGAATCCACTGAGTAAACATGTGATTTACAATGG
216	TCATTAGAGGAAGATCACGATAACTATGAATCCACTGAGTAAACATGTGATTTACAATGG
164	TCATTAGAGGAAGATCACGATAACTATGAATCCACTGAGTAAACATGTGATTTACAATGG
63	TCATTAGAGGAAGATCACGATAACTATGAATCCACTGAGTAAACATGTGATTTACAATGG
161	TCATTAGAGGAAGATCACGATAACTATGAATCCACTGAGTAAACATGTGATTTACAATGG
183	TCATTAGAGGAAGATCACGATAACTATGAATCCACTGAGTAAACATGTGATTTACAATGG
18	TCATTAGAGGAAGATCACGATAACTATGAATCCACTGAGTAAACATGTGATTTACAATGG
90	TCATTAGAGGAAGATCACGATAACTATGAATCCACTGAGTAAACATGTGATTTACAATGG
162	TCATTAGAGGAAGATCACGATAACTATGAATCCACTGAGTAAACATGTGATTTACAATGG
177	TCATTAGAGGAAGATCACGATAACTATGAATCCACTGAGTAAACATGTGATTTACAATGG
97	TCATTAGAGGAAGATCACGATAACTATGAATCCACTGAGTAAACATGTGATTTACAATGG
231	TCATTAGAGGAAGATCACGATAACTATGAATCCACTGAGTAAACATGTGATTTACAATGG
143	TCATTAGAGGAAGATCACGATAACTATGAATCCACTGAGTAAACATGTGATTTACAATGG
11	TCATTAGAGGAAGATCACGATAACTATGAATCCACTGAGTAAACATGTGATTTACAATGG
153	TCATTAGAGGAAGATCACGATAACTATGAATCCACTGAGTAAACATGTGATTTACAATGG
138	TCATTAGAGGAAGATCACGATAACTATGAATCCACTGAGTAAACATGTGATTTACAATGG
185	TCATTAGAGGAAGATCACGATAACTATGAATCCACTGAGTAAACATGTGATTTACAATGG

```
194           TCATTAGAGGAAGATCACGATAACTATGAATCCACTGAGTAAACATGTGATTTACAATGG
236           TCATTAGAGGAAGATCACGATAACTATGAATCCACTGAGTAAACATGTGATTTACAATGG
32            TCATTAGAGGAAGATCACGATAACTATGAATCCACTGAGTAAACATGTGATTTACAATGG
52            TCATTAGAGGAAGATCACGATAACTATGAATCCACTGAGTAAACATGTGATTTACAATGG
60            TCATTAGAGGAAGATCACGATAACTATGAATCCACTGAGTAAACATGTGATTTACAATGG
72            TCATTAGAGGAAGATCACGATAACTATGAATCCACTGAGTAAACATGTGATTTACAATGG
112           TCATTAGAGGAAGATCACGATAACTATGAATCCACTGAGTAAACATGTGATTTACAATGG
141           TCATTAGAGGAAGATCACGATAACTATGAATCCACTGAGTAAACATGTGATTTACAATGG
155           TCATTAGAGGAAGATCACGATAACTATGAATCCACTGAGTAAACATGTGATTTACAATGG
1             TCATTAGAGGAAGATCACGATAACTATGAATCCACTGAGTAAACATGTGATTTACAATGG
6             TCATTAGAGGAAGATCACGATAACTATGAATCCACTGAGTAAACATGTGATTTACAATGG
58            TCATTAGAGGAAGATCACGATAACTATGAATCCACTGAGTAAACATGTGATTTACAATGG
68            TCATTAGAGGAAGATCACGATAACTATGAATCCACTGAGTAAACATGTGATTTACAATGG
180           TCATTAGAGGAAGATCACGATAACTATGAATCCACTGAGTAAACATGTGATTTACAATGG
81            TCATTAGAGGAAGATCACGATAACTATGAATCCACTGAGTAAACATGTGATTTACAATGG
103           TCATTAGAGGAAGATCACGATAACTATGAATCCACTGAGTAAACATGTGATTTACAATGG
113           TCATTAGAGGAAGATCACGATAACTATGAATCCACTGAGTAAACATGTGATTTACAATGG
163           TCATTAGAGGAAGATCACGATAACTATGAATCCACTGAGTAAACATGTGATTTACAATGG
40            TCATTAGAGGAAGATCACGATAACTATGAATCCACTGAGTAAACATGTGATTTACAATGG
56            TCATTAGAGGAAGATCACGATAACTATGAATCCACTGAGTAAACATGTGATTTACAATGG
218           TCATTAGAGGAAGATCACGATAACTATGAATCCACTGAGTAAACATGTGATTTACAATGG
41            TCATTAGAGGAAGATCACGATAACTATGAATCCACTGAGTAAACATGTGATTTACAATGG
42            TCATTAGAGGAAGATCACGATAACTATGAATCCACTGAGTAAACATGTGATTTACAATGG
160           TCATTAGAGGAAGATCACGATAACTATGAATCCACTGAGTAAACATGTGATTTACAATGG
5             TCATTAGAGGAAGATCACGATAACTATGAATCCACTGAGTAAACATGTGATTTACAATGG
228           TCATTAGAGGAAGATCACGATAACTATGAATCCACTGAGTAAACATGTGATTTACAATGG
17            TCATTAGAGGAAGATCACGATAACTATGAATCCACTGAGTAAACATGTGATTTACAATGG
37            TCATTAGAGGAAGATCACGATAACTATGAATCCACTGAGTAAACATGTGATTTACAATGG
91            TCATTAGAGGAAGATCACGATAACTATGAATCCACTGAGTAAACATGTGATTTACAATGG
140           TCATTAGAGGAAGATCACGATAACTATGAATCCGCTGAGGAAACATGTGATTTACAATGG
167           TCATTAGAGGAAGATCACGATAACTATGAATCCACTGAGTAAACATGTGATTTACAATGG
              ********************************* ***** ********************

172           TTTCGCTAGTAGGTCGAACCTGCCAGAACCTCACGAAGTGCGGCAATCAAAGTCAAACCA
229           TTTCGCTAGTAGGTCGAACCTGCCAGAACCTCACGAAGTGCGGCAATCAAAGTCAAACCA
208           TTTCGCTAGTAGGTCGAACCTGCCAGAACCTCACGAAGTGCGGCAATCAAAGTCAAACCA
83            TTTCGCTAGTAGGTCGAACCTGCCAGAACCTCACGAAGTGCGGCAATCAAAGTCAAACCG
3             TTTCGCTAGTAGGTCGAACCTGCCAGAACCTCACGAAGTGCGGCAATCAAAGTCAAACCG
120           TTTCGCTAGTAGGTCGAACCTGCCAGAACCTCACGAAGTGCGGTAATCAAAGTCAAACCA
99            TTTCGCTAGTAGGTCGAACCTGCCAGAACCTCACGAAGTGCGGCAATCAAAGTCAAACCG
126           TTTCGCTAGTAGGTCGAACCTGCCAGAACCTCACGAAGTGCGGCAATCAAAGTCAAACCG
173           TTTCGCTAGTAGGTCGAACCTGCCAGAACCTCACGAAGTGCGGCAATCAAAGTCAAACCG
204           TTTCGCTAGTAGGTCGAACCTGCCAGAACCTCACGAAGTGCGGTAATCAAAGTCAAACCG
239           TTTCGCTAGTAGGTCGAACCTGCCAGAACCTCACGAAGTGCGGTAATCAAAGTCAAACCG
117           TTTCGCTAGTAGGTCGAACCTGCCAGAACCTCACGAAGTGCGGTAATCAAAGTCAAACCG
123           TTTCGCTAGTAGGTCGAACCTGCCAGAACCTCACGAAGTGCGGTAATCAAAGTCAAACCG
210           TTTCGCTAGTAGGTCGAACCTGCCAGAACCTCACGAAGTGCGGCAATCAAAGTCAAACCG
86            TTTCGCTAGTAGGTCGAACCTGCCAGAACCTCACGAAGTGCGGCAATCAAAGTCAAACCG
100           TTTCGCTAGTAGGTCGAACCTGCCAGAACCTCACGAAGTGCGGCAATCAAAGTCAAACCG
114           TTTCGCTAGTAGGTCGAACCTGCCAGAACCTCACGAAGTGCGGCAATCAAAGTCAAACCG
170           TTTCGCTAGTAGGTCGAACCTGCCAGAACCTCACGAAGTGCGGCAATCAAAGTCAAACCG
108           TTTCGCTAGTAGGTCGAACCTGCCAGAACCTCACGAAGTGCGGCATTCAAAGTCAAACCA
174           TTTCGCTAGTAGGTCGAACCTGCCAGAACCTCACGAAGTGCGGCATTCAAAGTCAAACCA
158           TTTCGCTAGTAGGTCGAACCTGCCAGAACCTCACGAAGTGCGGCAATCAAAGTCAAACCG
154           TTTCGCTAGTAGGTCGAACCTGCCAGAACCTCACGAAGTGCGGCAATCAAAGTCAAACCA
30            TTTCGCTAGTAGGTCGAACCTGCCAGAACCTCACGAAGTGCGGCAATCAAAGTCAAACCG
39            TTTCGCTAGTAGGTCGAACCTGCCAGAACCTCACGAAGTGCGGCAATCAAAGTCAAACCG
```

237	TTTCGCTAGTAGGTCGAACCTGCCAGAACCTCACGAAGTGCGGCAATCAAAGTCAAACCG
65	TTTCGCTAGTAGGTCGAACCTGCCAGAACCTCACGAAGTGCGGCAATCAAAGTCAAACCG
44	TTTCGCTAGTAGGTCGAACCTGCCAGAACCTCACGAAGTGCGGCAATCAAAGTCAAACCG
168	TTTCGCTAGTAGGTCGAACCTGCCAGAACCTCACGAAGTGCGGCAATCAAAGTCAAACCG
95	TTTCGCTAGTAGGTCGAACCTGCCAGAACCTCACGAAGTGCGGTAATCAAAGTCAAACCG
22	TTTCGCTAGTAGGTCGAACCTGCCAGAACCTCACGAAGTGCGGCAATCAAAGTCAAACCG
79	TTTCGCTAGTAGGTCGAACCTGCCAGAACCTCACGAAGTGCGGCAATCAAAGTCAAACCG
211	TTTCGCTAGTAGGTCGAACCTGCCAGAACCTCACGAAGTGCGGCAATCAAAGTCAAACCG
88	TTTCGCTAGTAGGTCGAACCTGCCAGAACCTCACGAAGTGCGGCAATCAAAGTCAAACCA
184	TTTCGCTAGTAGGTCGAACCTGCCAGAACCTCACGAAGTGCGGCAATCAAAGTCAAACCA
78	TTTCGCTAGTAGGTCGAACCTGCCAGAACCTCACGAAGTGCGGCAATCAAAGTCAAACCA
71	TTTCGCTAGTAGGTCGAACCTGCCAGAACCTCACGAAGTGCGGCAATCAAAGTCAAACCG
196	TTTCGCTAGTAGGTCGAACCTGCCAGAACCTCACGAAGTGCGGCAATCAAAGTCAAACCG
43	TTTCGCTAGTAGGTCGAACCTGCCAGAACCTCACGAAGTGCGGTAATCAAAGTCAAACCA
151	TTTCGCTAGTAGGTCGAACCTGCCAGAACCTCACGAAGTGCGGTAATCAAAGTCAAACCG
27	TTTCGCTAGTAGGTCGAACCTGCCAGAACCTCACGAAGTGCGGCAATCAAAGTCAAACCG
197	TTTCGCTAGTAGGTCGAACCTGCCAGAACCTCACGAAGTGCGGTAATCAAAGTCAAACCG
109	TTTCGCTAGTAGGTCGAACCTGCCAGAACCTCACGAAGTGCGGTAATCAAAGTCAAACCA
169	TTTCGCTAGTAGGTCGAACCTGCCAGAACCTCACGAAGTGCGGTAATCAAAGTCAAACCA
94	TTTCGCTAGTAGGTCGAACCTGCCAGAACCTCACGAAGTGCGGCAATCAAAGTCAAACCG
232	TTTCGCTAGTAGGTCGAACCTGCCAGAACCTCACGAAGTGCGGCAATCAAAGTCAAACCG
222	TTTCGCTAGTAGGTCGAACCTGCCAGAACCTCACGAAGTGCGGCAATCAAAGTCAAACCA
24	TTTCGCTAGTAGGTCGAACCTGCCAGAACCTCACGAAGTGCGGCAATCAAAGTCAAACCA
15	TTTCGCTAGTAGGTCGAACCTGCCAGAACCTCACGAAGTGCGGCAATCAAAGTCAAACCA
13	TTTCGCTAGTAGGTCGAACCTGCCAGAACCTCACGAAGTGCGGCAATCAAAGTCAAACCA
14	TTTCGCTAGTAGGTCGAACCTGCCAGAACCTCACGAAGTGCGGCATTCAAAGTCAAACCG
35	TTTCGCTAGTAGGTCGAACCTGCCAGAACCTCACGAAGTGCGGCATTCAAAGTCAAACCG
111	TTTCGCTAGTAGGTCGAACCTGCCAGAACCTCACGAAGTGCGGCATTCAAAGTCAAACCG
157	TTTCGCTAGTAGGTCGAACCTGCCAGAACCTCACGAAGTGCGGCAATCAAAGTCAAACCG
31	TTTCGCTAGTAGGTCGAACCTGCCAGAACCTCACGAAGTGCGGCAATCAAAGTCAAACCG
47	TTTCGCTAGTAGGTCGAACCTGCCAGAACCTCACGAAGTGCGGCAATCAAAGTCAAACCG
33	TTTCGCTAGTAGGTCGAACCTGCCAGAACCTCACGAAGTGCGGCAATCAAAGTCAAACCG
51	TTTCGCTAGTAGGTCGAACCTGCCAGAACCTCACGAAGTGCGGCAATCAAAGTCAAACCG
85	TTTCGCTAGTAGGTCGAACCTGCCAGAACCTCACGAAGTGCGGCAATCAAAGTCAAACCG
54	TTTCGCTAGTAGGTCGAACCTGCCAGAACCTCACGAAGTGCGGCAATCAAAGTCAAACCG
96	TTTCGCTAGTAGGTCGAACCTGCCAGAACCTCACGAAGTGCGGCAATCAAAGTCAAACCG
61	TTTCGCTAGTAGGTCGAACCTGCCAGAACCTCACGAAGTGCGGCAATCAAAGTCAAACCA
80	TTTCGCTAGTAGGTCGAACCTGCCAGAACCTCACGAAGTGCGGCAATCAAAGTCAAACCA
82	TTTCGCTAGTAGGTCGAACCTGCCAGAACCTCACGAAGTGCGGCAATCAAAGTCAAACCA
129	TTTCGCTAGTAGGTCGAACCTGCCAGAACCTCACGAAGTGCGGCAATCAAAGTCAAACCA
46	TTTCGCTAGTAGGTCGAACCTGCCAGAACCTCACGAAGTGCGGCAATCAAAGTCAAACCA
74	TTTCGCTAGTAGGTCGAACCTGCCAGAACCTCACGAAGTGCGGCAATCAAAGTCAAACCA
101	TTTCGCTAGTAGGTCGAACCTGCCAGAACCTCACGAAGTGCGGCAATCAAAGTCAAACCG
66	TTTCGCTAGTAGGTCGAACCTGCCAGAACCTCACGAAGTGCGGCAATCAAAGTCAAACCG
104	TTTCGCTAGTAGGTCGAACCTGCCAGAACCTCACGAAGTGCGGCAATCAAAGTCAAACCG
118	TTTCGCTAGTAGGTCGAACCTGCCAGAACCTCACGAAGTGCGGCAATCAAAGTCAAACCG
21	TTTCGCTAGTAGGTCGAACCTGCCAGAACCTCACGAAGTGCGGCAATCAAAGTCAAACCG
77	TTTCGCTAGTAGGTCGAACCTGCCAGAACCTCACGAAGTGCGGCAATCAAAGTCAAACCG
124	TTTCGCTAGTAGGTCGAACCTGCCAGAACCTCACGAAGTGCGGCAATCAAAGTCAAACCG
20	TTTCGCTAGTAGGTCGAACCTGCCAGAACCTCACGAAGTGCGGCAATCAAAGTCAAACCG
110	TTTCGCTAGTAGGTCGAACCTGCCAGAACCTCACGAAGTGCGGCAATCAAAGTCAAACCG
70	TTTCGCTAGTAGGTCGAACCTGCCAGAACCTCACGAAGTGCGGCAATCAAAGTCAAACCG
8	TTTCGCTAGTAGGTCGAACCTGCCAGAACCTCACGAAGTGCGGCAATCAAAGTCAAACCG
55	TTTCGCTAGTAGGTCGAACCTGCCAGAACCTCACGAAGTGCGGCAATCAAAGTCAAACCG
64	TTTCGCTAGTAGGTCGAACCTGCCAGAACCTCACGAAGTGCGGCAATCAAAGTCAAACCG
93	TTTCGCTAGTAGGTCGAACCTGCCAGAACCTCACGAAGTGCGGCAATCAAAGTCAAACCG
202	TTTCGCTAGTAGGTCGAACCTGCCAGAACCTCACGAAGTGCGGCAATCAAAGTCAAACCG

10	TTTCGCTAGTAGGTCGAACCTGCCAGAACCTCACGAAGTGCGGCAATCAAAGTCAAACCG
16	TTTCGCTAGTAGGTCGAACCTGCCAGAACCTCACGAAGTGCGGCAATCAAAGTCAAACCG
76	TTTCGCTAGTAGGTCGAACCTGCCAGAACCTCACGAAGTGCGGCAATCAAAGTCAAACCG
116	TTTCGCTAGTAGGTCGAACCTGCCAGAACCTCACGAAGTGCGGCAATCAAAGTCAAACCG
220	TTTCGCTAGTAGGTCGAACCTGCCAGAACCTCACGAAGTGCGGCAATCAAAGTCAAACCG
234	TTTCGCTAGTAGGTCGAACCTGCCAGAACCTCACGAAGTGCGGCAATCAAAGTCAAACCG
2	TTTCGCTAGTAGGTCGAACCTGCCAGAACCTCACGAAGTGCGGCAATCAAAGTCAAACCG
38	TTTCGCTAGTAGGTCGAACCTGCCAGAACCTCACGAAGTGCGGCAATCAAAGTCAAACCG
73	TTTCGCTAGTAGGTCGAACCTGCCAGAACCTCACGAAGTGCGGCAATCAAAGTCAAACCG
87	TTTCGCTAGTAGGTCGAACCTGCCAGAACCTCACGAAGTGCGGCAATCAAAGTCAAACCG
92	TTTCGCTAGTAGGTCGAACCTGCCAGAACCTCACGAAGTGCGGCAATCAAAGTCAAACCG
98	TTTCGCTAGTAGGTCGAACCTGCCAGAACCTCACGAAGTGCGGCAATCAAAGTCAAACCG
137	TTTCGCTAGTAGGTCGAACCTGCCAGAACCTCACGAAGTGCGGCAATCAAAGTCAAACCG
19	TTTCGCTAGTAGGTCGAACCTGCCAGAACCTCACGAAGTGCGGCAATCAAAGTCAAACCG
171	TTTCGCTAGTAGGTCGAACCTGCCAGAACCTCACGAAGTGCGGCAATCAAAGTCAAACCG
205	TTTCGCTAGTAGGTCGAACCTGCCAGAACCTCACGAAGTGCGGCAATCAAAGTCAAACCG
75	TTTCGCTAGTAGGTCGAACCTGCCAGAACCTCACGAAGTGCGGCAATCAAAGTCAAACCG
62	TTTCGCTAGTAGGTCGAACCTGCCAGAACCTCACGAAGTGCGGCAATCAAAGTCAAACCG
4	TTTCGCTAGTAGGTCGAACCTGCCAGAACCTCACGAAGTGCGGCAATCAAAGTCAAACCG
7	TTTCGCTAGTAGGTCGAACCTGCCAGAACCTCACGAAGTGCGGCAATCAAAGTCAAACCG
25	TTTCGCTAGTAGGTCGAACCTGCCAGAACCTCACGAAGTGCGGCAATCAAAGTCAAACCG
28	TTTCGCTAGTAGGTCGAACCTGCCAGAACCTCACGAAGTGCGGCAATCAAAGTCAAACCG
36	TTTCGCTAGTAGGTCGAACCTGCCAGAACCTCACGAAGTGCGGCAATCAAAGTCAAACCG
49	TTTCGCTAGTAGGTCGAACCTGCCAGAACCTCACGAAGTGCGGCAATCAAAGTCAAACCG
145	TTTCGCTAGTAGGTCGAACCTGCCAGAACCTCACGAAGTGCGGCAATCAAAGTCAAACCG
181	TTTCGCTAGTAGGTCGAACCTGCCAGAACCTCACGAAGTGCGGCAATCAAAGTCAAACCG
188	TTTCGCTAGTAGGTCGAACCTGCCAGAACCTCACGAAGTGCGGCAATCAAAGTCAAACCG
59	TTTCGCTAGTAGGTCGAACCTGCCAGAACCTCACGAAGTGCGGCAATCAAAGTCAAACCG
121	TTTCGCTAGTAGGTCGAACCTGCCAGAACCTCACGAAGTGCGGCAATCAAAGTCAAACCG
127	TTTCGCTAGTAGGTCGAACCTGCCAGAACCTCACGAAGTGCGGCAATCAAAGTCAAACCG
135	TTTCGCTAGTAGGTCGAACCTGCCAGAACCTCACGAAGTGCGGCAATCAAAGTCAAACCG
136	TTTCGCTAGTAGGTCGAACCTGCCAGAACCTCACGAAGTGCGGCAATCAAAGTCAAACCG
29	TTTCGCTAGTAGGTCGAACCTGCCAGAACCTCACGAAGTGCGGCAATCAAAGTCAAACCG
89	TTTCGCTAGTAGGTCGAACCTGCCAGAACCTCACGAAGTGCGGCAATCAAAGTCAAACCG
216	TTTCGCTAGTAGGTCGAACCTGCCAGAACCTCACGAAGTGCGGCAATCAAAGTCAAACCG
164	TTTCGCTAGTAGGTCGAACCTGCCAGAACCTCACGAAGTGCGGCAATCAAAGTCAAACCG
63	TTTCGCTAGTAGGTCGAACCTGCCAGAACCTCACGAAGTGCGGCAATCAAAGTCAAACCG
161	TTTCGCTAGTAGGTCGAACCTGCCAGAACCTCACGAAGTGCGGCAATCAAAGTCAAACCG
183	TTTCGCTAGTAGGTCGAACCTGCCAGAACCTCACGAAGTGCGGCAATCAAAGTCAAACCG
18	TTTCGCTAGTAGGTCGAACCTGCCAGAACCTCACGAAGTGCGGCAATCAAAGTCAAACCG
90	TTTCGCTAGTAGGTCGAACCTGCCAGAACCTCACGAAGTGCGGCAATCAAAGTCAAACCG
162	TTTCGCTAGTAGGTCGAACCTGCCAGAACCTCACGAAGTGCGGCAATCAAAGTCAAACCG
177	TTTCGCTAGTAGGTCGAACCTGCCAGAACCTCACGAAGTGCGGCAATCAAAGTCAAACCG
97	TTTCGCTAGTAGGTCGAACCTGCCAGAACCTCACGAAGTGCGGTAATCAAAGTCAAACCG
231	TTTCGCTAGTAGGTCGAACCTGCCAGAACCTCACGAAGTGCGGCAATCAAAGTCAAACCG
143	TTTCGCTAGTAGGTCGAACCTGCCAGAACCTCACGAAGTGCGGCAATCAAAGTCAAACCG
11	TTTCGCTAGTAGGTCGAACCTGCCAGAACCTCACGAAGTGCGGCAATCAAAGTCAAACCG
153	TTTCGCTAGTAGGTCGAACCTGCCAGAACCTCACGAAGTGCGGCAATCAAAGTCAAACCG
138	TTTCGCTAGTAGGTCGAACCTGCCAGAACCTCACGAAGTGCGGCAATCAAAGTCAAACCG
185	TTTCGCTAGTAGGTCGAACCTGCCAGAACCTCACGAAGTGCGGCAATCAAAGTCAAACCG
194	TTTCGCTAGTAGGTCGAACCTGCCAGAACCTCACGAAGTGCGGCAATCAAAGTCAAACCG
236	TTTCGCTAGTAGGTCGAACCTGCCAGAACCTCACGAAGTGCGGCAATCAAAGTCAAACCG
32	TTTCGCTAGTAGGTCGAACCTGCCAGAACCTCACGAAGTGCGGCAATCAAAGTCAAACCG
52	TTTCGCTAGTAGGTCGAACCTGCCAGAACCTCACGAAGTGCGGCAATCAAAGTCAAACCG
60	TTTCGCTAGTAGGTCGAACCTGCCAGAACCTCACGAAGTGCGGCAATCAAAGTCAAACCG
72	TTTCGCTAGTAGGTCGAACCTGCCAGAACCTCACGAAGTGCGGCAATCAAAGTCAAACCG
112	TTTCGCTAGTAGGTCGAACCTGCCAGAACCTCACGAAGTGCGGCAATCAAAGTCAAACCG

141	TTTCGCTAGTAGGTCGAACCTGCCAGAACCTCACGAAGTGCGGCAATCAAAGTCAAACCG
155	TTTCGCTAGTAGGTCGAACCTGCCAGAACCTCACGAAGTGCGGCAATCAAAGTCAAACCG
1	TTTCGCTAGTAGGTCGAACCTGCCAGAACCTCACGAAGTGCGGCAATCAAAGTCAAACCG
6	TTTCGCTAGTAGGTCGAACCTGCCAGAACCTCACGAAGTGCGGCAATCAAAGTCAAACCG
58	TTTCGCTAGTAGGTCGAACCTGCCAGAACCTCACGAAGTGCGGCAATCAAAGTCAAACCG
68	TTTCGCTAGTAGGTCGAACCTGCCAGAACCTCACGAAGTGCGGCAATCAAAGTCAAACCG
180	TTTCGCTAGTAGGTCGAACCTGCCAGAACCTCACGAAGTGCGGCAATCAAAGTCAAACCG
81	TTTCGCTAGTAGGTCGAACCTGCCAGAACCTCACGAAGTGCGGCAATCAAAGTCAAACCG
103	TTTCGCTAGTAGGTCGAACCTGCCAGAACCTCACGAAGTGCGGCAATCAAAGTCAAACCG
113	TTTCGCTAGTAGGTCGAACCTGCCAGAACCTCACGAAGTGCGGCAATCAAAGTCAAACCG
163	TTTCGCTAGTAGGTCGAACCTGCCAGAACCTCACGAAGTGCGGCAATCAAAGTCAAACCG
40	TTTCGCTAGTAGGTCGAACCTGCCAGAACCTCACGAAGTGCGGTAATCAAAGTCAAACCG
56	TTTCGCTAGTAGGTCGAACCTGCCAGAACCTCACGAAGTGCGGTAATCAAAGTCAAACCG
218	TTTCGCTAGTAGGTCGAACCTGCCAGAACCTCACGAAGTGCGGTAATCAAAGTCAAACCG
41	TTTCGCTAGTAGGTCGAACCTGCCAGAACCTCACGAAGTGCGGTAATCAAAGTCAAACCG
42	TTTCGCTAGTAGGTCGAACCTGCCAGAACCTCACGAAGTGCGGTAATCAAAGTCAAACCG
160	TTTCGCTAGTAGGTCGAACCTGCCAGAACCTCACGAAGTGCGGCAATCAAAGTCAAACCG
5	TTTCGCTAGTAGGTCGAACCTGCCAGAACCTCACGAAGTGCGGTAATCAAAGTCAAACCG
228	TTTCGCTAGTAGGTCGAACCTGCCAGAACCTCACGAAGTGCGGTAATCAAAGTCAAACCG
17	TTTCGCTAGTAGGTCGAACCTGCCAGAACCTCACGAAGTGCGGTAATCAAAGTCAAACCG
37	TTTCGCTAGTAGGTCGAACCTGCCAGAACCTCACGAAGTGCGGTAATCAAAGTCAAACCG
91	TTTCGCTAGTAGGTCGAACCTGCCAGAACCTCACGAAGTGCGGTAATCAAAGTCAAACCG
140	TTTCGCTAGTAGGTCGAACCTGCCAGAACCTCACGAAGTGCGGCAATCAAAGTCAAACCG
167	TTTCGCTAGTAGGTCGAACCTGCCAGAACCTCACGAAGTGCGGCAATCAAAGTCAAACCA
	*** * *************

172	CAGGTCAAAACATTTTAAACTTGAACCAGCAACATCAATTAAACCTAGCAGCTTAGAACT
229	CAGGTCAAAACATTTTAAACTTGAACCAGCAACATCAATTAAACCTAGCAGCTTAGAACT
208	CAGGTCAAAACATTTTAAACTTGAACCAGCAACATCAATTAAACCTAGCAGCTTAGAACT
83	CAGGTCAAAACATTTTAAACTTGAACCAGCAATATCAATTAAACCTAGCAGCTTAGAACT
3	CAGGTCAAAACATTTTAAACTTGAACCAGCAATATCAATTAAACCTAGCAGCTTAGAACT
120	CAGGTCAAAACATTTTAAACTTGAACCAGCAACATCAATTAAACCTAGCAGCTTAAAACT
99	CAGGTCAAAACATTTTAAACTTGAACCAGCAACATCAATTAAACCTAGCAGCTTAAAACT
126	CAGGTCAAAACATTTTAAACTTGAACCAGCAACATCAATTAAACCTAGCAGCTTAAAACT
173	CAGGTCAAAACATTTTAAACTTGAACCAGCAACATCAATTAAACCTAGCAGCTTAAAACT
204	CAGGTCAAAACATTTTAAACTTGAACCAGCAACATCAATTTAACCTAGCAGCTTAAAACT
239	CAGGTCAAAACATTTTAAACTTGAACCAGCAACATCAATTAAACCTAGCAGCTTAAAACT
117	CAGGTCAAAACATTTTAAACTTGAACCAGCAACATCAATTAAACCTAGCAGCTTAAAACT
123	CAGGTCAAAACATTTTAAACTTGAACCAGCAACATCAATTAAACCTAGCAGCTTAAAACT
210	CAGGTCAAAACATTTTAAACTTGAACCAGCAATATCAATTAAACCTAGCAGCTTAGAACT
86	CAGGTCAAAACATTTTAAACTTGAACCAGCAATATCAATTAAACCTAGCAGCTTAGAACT
100	CAGGTCAAAACATTTTAAACTTGAACCAGCAATATCAATTAAACCTAGCAGCTTAGAACT
114	CAGGTCAAAACATTTTAAACTTGAACCAGCAATATCAATTTAACCTAGCAGCTTAAAACT
170	CAGGTCAAAACATTTTAAACTTGAACCAGCAATATCAATTTAACCTAGCAGCTTAAAACT
108	CAGGTCAAAACATTTTAAACTTGAACCAGCAATATCAATTTAACCTAGCAGCTTAAAACT
174	CAGGTCAAAACATTTTAAACTTGAACCAGCAATATCAATTTAACCTAGCAGCTTAAAACT
158	CAGGTCAAAACATTTTAAACTTGAACCAGCAACATCAATTTAACCTAGCAGCTTAAAACT
154	CAGGTCAAAACATTTTAAACTTGAACCAGCAACATCAATTTAACCTAGCAGCTTAAAACT
30	CAGGTCAAAACATTTTAAACTTGAACCAGCAACATCAATTTAACCTAGCAGCTTAAAACT
39	CAGGTCAAAACATTTTAAACTTGAACCAGCAACATCAATTTAACCTAGCAGCTTAAAACT
237	CAGGTCAAAACATTTTAAACTTGAACCAGCAATATCAATTTAACCTAGCAGCTTAAAACT
65	CAGGTCAAAACATTTTAAACTTGAACCAGCAATATCAATTTAACCTAGCAGCTTAAAACT
44	CAGGTCAAAACATTTTAAACTTGAACCAGCAACATCAATTAAACCTAGCAGCTTAAAACT
168	CAGGTCAAAACATTTTAAACTTGAACCAGCAACATCAATTAAACCTAGCAGCTTAGAACT
95	CAGGTCAAAACATTTTAAACTTGAACCAGCAATATCAATTAAACCTAGCAGCTTAAAACT
22	CAGGTCAAAACATTTTAAACTTGAACCAGCAATATCAATTTAACCTAGCAGCTTAAAACT
79	CAGGTCAAAACATTTTAAACTTGAACCAGCAACATCAATTTAACCTAGCAGCTTAAAACT

211	CAGGTCAAAACATTTTAAACTTGAACCAGCAACATCAATTTAACCTAGCAGCTTAAAACT
88	CAGGTCAAAACATTTTAAACTTGAACCAGCAACATCAATTAAACCTAGCAGCTTAAAACT
184	CAGGTCAAAACATTTTAAACTTGAACCAGCAACATCAATTAAACCTAGCAGCTTAAAACT
78	CAGGTCAAAACATTTTAAACTTGAACCAGCAACATCAATTTAACCTAGCAGCTTAAAACT
71	CAGGTCAAAACATTTTAAACTTGAACCAGCAATATCAATTAAACCTAGCAGCTTAAAACT
196	CAGGTCAAAACATTTTAAACTTGAACCAGCAATATCAATTAAACCTAGCAGCTTAAAACT
43	CAGGTCAAAACATTTTAAACTTGAACCAGCAATATCAATTAAACCTAGCAGCTTAAAACT
151	CAGGTCAAAACATTTTAAACTTGAACCAGCAATATCAATTAAACCTAGCAGCTTAAAACT
27	CAGGTCAAAACATTTTAAACTTGAACCAGCAACATCAATTTAACCTAGCAGCTTAAAACT
197	CAGGTCAAAACATTTTAAACTTGAACCAGCAATATCAATTTAACCTAGCAGCTTAAAACT
109	CAGGTCAAAACATTTTAAACTTGAACCAGCAACATCAATTAAACCTAGCAGCTTAAAACT
169	CAGGTCAAAACATTTTAAACTTGAACCAGCAACATCAATTTAACCTAGCAGCTTAAAACT
94	CAGGTCAAAACATTTTAAACTTGAACCAGCAACATCAATTAAACCTAGCAGCTTAGAACT
232	CAGGTCAAAACATTTTAAACTTGAACCAGCAACATCAATTAAACCTAGCAGCTTAGAACT
222	CAGGTCAAAACATTTTAAACTTGAACCAGCAACATCAATTAAACCTAGCAGCTTAAAACT
24	CAGGTCAAAACATTTTAAACTTGAACCAGCAACATCAATTAAACCTAGCAGCTTAAAACT
15	CAGGTCAAAACATTTTAAACTTGAACCAGCAACATCAATTAAACCTAGCAGCTTAAAACT
13	CAGGTCAAAACATTTTAAACTTGAACCAGCAACATCAATTAAACCTAGCAGCTTAAAACT
14	CAGGTCAAAACATTTTAAACTTGAACCAGCAACATCAATTAAACCTAGCAGCTTAAAACT
35	CAGGTCAAAACATTTTAAACTTGAACCAGCAACATCAATTAAACCTAGCAGCTTAAAACT
111	CAGGTCAAAACATTTTAAACTTGAACCAGCAACATCAATTAAACCTAGCAGCTTAAAACT
157	CAGGTCAAAACATTTTAAACTTGAACCAGCAATATCAATTAAACCTAGCAGCTTAAAACT
31	CAGGTCAAAACATTTTAAACTTGAACCAGCAATATCAATTAAACCTAGCAGCTTAAAACT
47	CAGGTCAAAACATTTTAAACTTGAACCAGCAATATCAATTAAACCTAGCAGCTTAAAACT
33	CAGGTCAAAACATTTTAAACTTGAACCAGCAATATCAATTAAACCTAGCAGCTTAAAACT
51	CAGGTCAAAACATTTTAAACTTGAACCAGCAATATCAATTAAACCTAGCAGCTTAAAACT
85	CAGGTCAAAACATTTTAAACTTGAACCAGCAATATCAATTAAACCTAGCAGCTTAAAACT
54	CAGGTCAAAACATTTTAAACTTGAACCAGCAATATCAATTAAACCTAGCAGCTTAAAACT
96	CAGGTCAAAACATTTTAAACTTGAACCAGCAATATCAATTAAACCTAGCAGCTTAAAACT
61	CAGGTCAAAACATTTTAAACTTGAACCAGCAACATCAATTAAACCTAGCAGCTTAAAACT
80	CAGGTCAAAACATTTTAAACTTGAACCAGCAACATCAATTAAACCTAGCAGCTTAAAACT
82	CAGGTCAAAACATTTTAAACTTGAACCAGCAACATCAATTAAACCTAGCAGCTTAAAACT
129	CAGGTCAAAACATTTTAAACTTGAACCAGCAACATCAATTAAACCTAGCAGCTTAAAACT
46	CAGGTCAAAACATTTTAAACTTGAACCAGCAACATCAATTAAACCTAGCAGCTTAAAACT
74	CAGGTCAAAACATTTTAAACTTGAACCAGCAATATCAATTAAACCTAGCAGCTTAAAACT
101	CAGGTCAAAACATTTTAAACTTGAACCAGCAACATCAATTAAACCTAGCAGCTTAAAACT
66	CAGGTCAAAACATTTTAAACTTGAACCAGCAACATCAATTAAACCTAGCAGCTTAAAACT
104	CAGGTCAAAACATTTTAAACTTGAACCAGCAACATCAATTAAACCTAGCAGCTTAAAACT
118	CAGGTCAAAACATTTTAAACTTGAACCAGCAACATCAATTAAACCTAGCAGCTTAAAACT
21	CAGGTCAAAACATTTTAAACTTGAACCAGCAACATCAATTAAACCTAGCAGCTTAAAACT
77	CAGGTCAAAACATTTTAAACTTGAACCAGCAACATCAATTAAACCTAGCAGCTTAAAACT
124	CAGGTCAAAACATTTTAAACTTGAACCAGCAACATCAATTAAACCTAGCAGCTTAAAACT
20	CAGGTCAAAACATTTTAAACTTGAACCAGCAACATCAATTAAACCTAGCAGCTTAAAACT
110	CAGGTCAAAACATTTTAAACTTGAACCAGCAACATCAATTAAACCTAGCAGCTTAAAACT
70	CAGGTCAAAACATTTTAAACTTGAACCAGCAACATCAATTAAACCTAGCAGCTTAAAACT
8	CAGGTCAAAACATTTTAAACTTGAACCAGCAACATCAATTAAACCTAGCAGCTTAAAACT
55	CAGGTCAAAACATTTTAAACTTGAACCAGCAACATCAATTAAACCTAGCAGCTTAAAACT
64	CAGGTCAAAACATTTTAAACTTGAACCAGCAACATCAATTAAACCTAGCAGCTTAAAACT
93	CAGGTCAAAACATTTTAAACTTGAACCAGCAACATCAATTAAACCTAGCAGCTTAAAACT
202	CAGGTCAAAACATTTTAAACTTGAACCAGCAACATCAATTAAACCTAGCAGCTTAAAACT
10	CAGGTCAAAACATTTTAAACTTGAACCAGCAACATCAATTAAACCTAGCAGCTTAAAACT
16	CAGGTCAAAACATTTTAAACTTGAACCAGCAACATCAATTAAACCTAGCAGCTTAAAACT
76	CAGGTCAAAACATTTTAAACTTGAACCAGCAACATCAATTAAACCTAGCAGCTTAAAACT
116	CAGGTCAAAACATTTTAAACTTGAACCAGCAACATCAATTAAACCTAGCAGCTTAAAACT
220	CAGGTCAAAACATTTTAAACTTGAACCAGCAACATCAATTAAACCTAGCAGCTTAAAACT
234	CAGGTCAAAACATTTTAAACTTGAACCAGCAACATCAATTAAACCTAGCAGCTTAAAACT
2	CAGGTCAAAACATTTTAAACTTGAACCAGCAACATCAATTAAACCTAGCAGCTTAAAACT

38	CAGGTCAAAACATTTTAAACTTGAACCAGCAACATCAATTAAACCTAGCAGCTTAAAACT
73	CAGGTCAAAACATTTTAAACTTGAACCAGCAACATCAATTAAACCTAGCAGCTTAAAACT
87	CAGGTCAAAACATTTTAAACTTGAACCAGCAACATCAATTAAACCTAGCAGCTTAAAACT
92	CAGGTCAAAACATTTTAAACTTGAACCAGCAACATCAATTAAACCTAGCAGCTTAAAACT
98	CAGGTCAAAACATTTTAAACTTGAACCAGCAACATCAATTAAACCTAGCAGCTTAAAACT
137	CAGGTCAAAACATTTTAAACTTGAACCAGCAACATCAATTAAACCTAGCAGCTTAAAACT
19	CAGGTCAAAACATTTTAAACTTGAACCAGCAACATCAATTAAACCTAGCAGCTTAAAACT
171	CAGGTCAAAACATTTTAAACTTGAACCAGCAACATCAATTAAACCTAGCAGCTTAAAACT
205	CAGGTCAAAACATTTTAAACTTGAACCAGCAACATCAATTTAACCTAGCAGCTTAAAACT
75	CAGGTCAAAACATTTTAAACTTGAACCAGCAACATCAATTTAACCTAGCAGCTTAAAACT
62	CAGGTCAAAACATTTTAAACTTGAACCAGCAACATCAATTTAACCTAGCAGCTTAAAACT
4	CAGGTCAAAACATTTTAAACTTGAACCAGCAACATCAATTTAACCTAGCAGCTTAAAACT
7	CAGGTCAAAACATTTTAAACTTGAACCAGCAACATCAATTTAACCTAGCAGCTTAAAACT
25	CAGGTCAAAACATTTTAAACTTGAACCAGCAACATCAATTTAACCTAGCAGCTTAAAACT
28	CAGGTCAAAACATTTTAAACTTGAACCAGCAACATCAATTTAACCTAGCAGCTTAAAACT
36	CAGGTCAAAACATTTTAAACTTGAACCAGCAACATCAATTTAACCTAGCAGCTTAAAACT
49	CAGGTCAAAACATTTTAAACTTGAACCAGCAACATCAATTTAACCTAGCAGCTTAAAACT
145	CAGGTCAAAACATTTTAAACTTGAACCAGCAACATCAATTTAACCTAGCAGCTTAAAACT
181	CAGGTCAAAACATTTTAAACTTGAACCAGCAACATCAATTTAACCTAGCAGCTTAAAACT
188	CAGGTCAAAACATTTTAAACTTGAACCAGCAACATCAATTTAACCTAGCAGCTTAAAACT
59	CAGGTCAAAACATTTTAAACTTGAACCAGCAACATCAATTTAACCTAGCAGCTTAAAACT
121	CAGGTCAAAACATTTTAAACTTGAACCAGCAACATCAATTTAACCTAGCAGCTTAAAACT
127	CAGGTCAAAACATTTTAAACTTGAACCAGCAACATCAATTTAACCTAGCAGCTTAAAACT
135	CAGGTCAAAACATTTTAAACTTGAACCAGCAACATCAATTTAACCTAGCAGCTTAAAACT
136	CAGGTCAAAACATTTTAAACTTGAACCAGCAACATCAATTTAACCTAGCAGCTTAAAACT
29	CAGGTCAAAACATTTTAAACTTGAACCAGCAACATCAATTTAACCTAGCAGCTTAAAACT
89	CAGGTCAAAACATTTTAAACTTGAACCAGCAACATCAATTTAACCTAGCAGCTTAAAACT
216	CAGGTCAAAACATTTTAAACTTGAACCAGCAACATCAATTTAACCTAGCAGCTTAAAACT
164	CAGGTCAAAACATTTTAAACTTGAACCAGCAATATCAATTTAACCTAGCAGCTTAAAACT
63	CAGGTCAAAACATTTTAAACTTGAACCAGCAATATCAATTTAACCTAGCAGCTTAAAACT
161	CAGGTCAAAACATTTTAAACTTGAACCAGCAATATCAATTTAACCTAGCAGCTTAAAACT
183	CAGGTCAAAACATTTTAAACTTGAACCAGCAATATCAATTTAACCTAGCAGCTTAAAACT
18	CAGGTCAAAACATTTTAAACTTGAACCAGCAATATCAATTTAACCTAGCAGCTTAAAACT
90	CAGGTCAAAACATTTTAAACTTGAACCAGCAACATCAATTTAACCTAGCAGCTTAAAACT
162	CAGGTCAAAACATTTTAAACTTGAACCAGCAACATCAATTTAACCTAGCAGCTTAAAACT
177	CAGGTCAAAACATTTTAAACTTGAACCAGCAACATCAATTAAACCTAGCAGCTTAAAACT
97	CAGGTCAAAACATTTTAAACTTGAACCAGCAACATCAATTAAACCTAGCAGCTTAAAACT
231	CAGGTCAAAACATTTTAAACTTGAACCAGCAACATCAATTAAACCTAGCAGCTTAAAACT
143	CAGGTCAAAACATTTTAAACTTGAACCAGCAATATCAATTAAACCTAGCAGCTTAAAACT
11	CAGGTCAAAACATTTTAAACTTGAACCAGCAACATCAATTAAACCTAGCAGCTTAAAACT
153	CAGGTCAAAACATTTTAAACTTGAACCAGCAACATCAATTAAACCTAGCAGCTTAAAACT
138	CAGGTCAAAACATTTTAAACTTGAACCAGCAACATCAATTAAACCTAGCAGCTTAAAACT
185	CAGGTCAAAACATTTTAAACTTGAACCAGCAACATCAATTAAACCTAGCAGCTTAAAACT
194	CAGGTCAAAACATTTTAAACTTGAACCAGCAACATCAATTAAACCTAGCAGCTTAAAACT
236	CAGGTCAAAACATTTTAAACTTGAACCAGCAACATCAATTAAACCTAGCAGCTTAAAACT
32	CAGGTCAAAACATTTTAAACTTGAACCAGCAACATCAATTAAACCTAGCAGCTTAAAACT
52	CAGGTCAAAACATTTTAAACTTGAACCAGCAACATCAATTAAACCTAGCAGCTTAAAACT
60	CAGGTCAAAACATTTTAAACTTGAACCAGCAACATCAATTAAACCTAGCAGCTTAAAACT
72	CAGGTCAAAACATTTTAAACTTGAACCAGCAACATCAATTAAACCTAGCAGCTTAAAACT
112	CAGGTCAAAACATTTTAAACTTGAACCAGCAACATCAATTAAACCTAGCAGCTTAAAACT
141	CAGGTCAAAACATTTTAAACTTGAACCAGCAACATCAATTAAACCTAGCAGCTTAAAACT
155	CAGGTCAAAACATTTTAAACTTGAACCAGCAACATCAATTAAACCTAGCAGCTTAAAACT
1	CAGGTCAAAACATTTTAAACTTGAACCAGCAACATCAATTAAACCTAGCAGCTTAAAACT
6	CAGGTCAAAACATTTTAAACTTGAACCAGCAACATCAATTAAACCTAGCAGCTTAAAACT
58	CAGGTCAAAACATTTTAAACTTGAACCAGCAACATCAATTAAACCTAGCAGCTTAAAACT
68	CAGGTCAAAACATTTTAAACTTGAACCAGCAACATCAATTAAACCTAGCAGCTTAAAACT
180	CAGGTCAAAACATTTTAAACTTGAACCAGCAACATCAATTAAACCTAGCAGCTTAAAACT

81 CAGGTCAAAACATTTTAAACTTGAACCAGCAACATCAATTAAACCTAGCAGCTTAAAACT
103 CAGGTCAAAACATTTTAAACTTGAACCAGCAACATCAATTAAACCTAGCAGCTTAAAACT
113 CAGGTCAAAACATTTTAAACTTGAACCAGCAACATCAATTAAACCTAGCAGCTTAAAACT
163 CAGGTCAAAACATTTTAAACTTGAACCAGCAACATCAATTAAACCTAGCAGCTTAAAACT
40 CAGGTCAAAACATTTTAAACTTGAACCAGCAATATCAATTAAACCTAGCAGCTTAAAACT
56 CAGGTCAAAACATTTTAAACTTGAACCAGCAATATCAATTAAACCTAGCAGCTTAAAACT
218 CAGGTCAAAACATTTTAAACTTGAACCAGCAATATCAATTAAACCTAGCAGCTTAAAACT
41 CAGGTCAAAACATTTTAAACTTGAACCAGCAATATCAATTAAACCTAGCAGCTTAAAACT
42 CAGGTCAAAACATTTTAAACTTGAACCAGCAACATCAATTTAACCTAGCAGCTTAAAACT
160 CAGGTCAAAACATTTTAAACTTGAACCAGCAACATCAATTAAACCTAGCAGCTTAAAACT
5 CAGGTCAAAACATTTTAAACTTGAACCAGCAACATCAATTAAACCTAGCAGCTTAAAACT
228 CAGGTCAAAACATTTTAAACTTGAACCAGCAACATCAATTAAACCTAGCAGCTTAAAACT
17 CAGGTCAAAACATTTTAAACTTGAACCAGCAACATCAATTAAACCTAGCAGCTTAAAACT
37 CAGGTCAAAACATTTTAAACTTGAACCAGCAACATCAATTAAACCTAGCAGCTTAAAACT
91 CAGGTCAAAACATTTTAAACTTGAACCAGCAACATCAATTAAACCTAGCAGCTTAAAACT
140 CAGGTCAAAACATTTTAAACTTGAACCAGCAACATCAATTTAACCTAGCAGCTTAGAACT
167 CAGGTCAAAACATTTTAAACTTGAACCAGCAACATCAATTTAACCTAGCAGCTTAGAACT
******************************* ******* *************** ****

172 TTAAATTAAAATAGGAAAAACTGTGATGGCAACTAAGGAGTGAAATAGAAAGGGGGAAAA
229 TTAAATTAAAATAGGAAAAACTGTGATGGCAACTAAGGAGTGAAATAGAAAGGGGGAAAA
208 TTAAATTAAAATAGGAAAAACTGTGATGGCAACTAAGGAGTGAAATAGAAAGGGGGAAAA
83 TTAAATTAAAATAGGAAAAACTGTGATGGCAACTAAGGAGTGAAATAGAAAGGGGGGGAA
3 TTAAATTAAAATAGGAAAAACTGTGATGGCAACTAAGGAGTGAAATAGAAAGGGGGGGAA
120 TTAAATTAAAATAGGAAAAACTGTGATGGCAACTAAGGAGTGAAATAGAAAGGGGGAGAA
99 TTAAATTAAAATAGGAAAAACTGTGATGGCAACTAAGGAGTGAAATAGAAAGGGGGGGAA
126 TTAAATTAAAATAGGAAAAACTGTGATGGCGACTAAGGAGTGAAATAGAAAGGGGGAGAA
173 TTAAATTAAAATAGGAAAAACTGTGATGGCGACTAAGGAGTGAAATAGAAAGGGGGAGAA
204 TTAAATTAAAATAGGAAAAACTGTGATGGCAACTAAGGAGTGAAATAGAAAGGGGGAGAA
239 TTAAATTAAAATAGGAAAAACTGTGATGGCAACTAAGGAGTGAAATAGAAAGGGGGAGAA
117 TTAAATTAAAATAGGAAAAACTGTGATGGCAACTAAGGAGTGAAATAGAAAGGGGGAGAA
123 TTAAATTAAAATAGGAAAAACTGTGATGGCAACTAAGGAGTGAAATAGAAAGGGGGAGAA
210 TTAAATTAAAATAGGAAAAACTGTGATGGCGACTAAGGAGTGAAATAGAAAGGGGGAGAA
86 TTAAATTAAAATAGGAAAAACTGTGATGGCGACTAAGGAGTGAAATAGAAAGGGGGAGAA
100 TTAAATTAAAATAGGAAAAACTGTGATGGCGACTAAGGAGTGAAATAGAAAGGGGGAGAA
114 TTAAATTAAAATAGGAAAAACTGTGATGGCGACTAAGGAGTGAAATAGAAAGGGGGAGAA
170 TTAAATTAAAATAGGAAAAACTGTGATGGCGACTAAGGAGTGAAATAGAAAGGGGGAGAA
108 TTAAATTAAAATAGGAAAAACTGTGATGGCAACTAAGGAGTGAAATAGAAAGGGGGGGAA
174 TTAAATTAAAATAGGAAAAACTGTGATGGCAACTAAGGAGTGAAATAGAAAGGGGGGGAA
158 TTAAATTAAAATAGGAAAAACTGTGATGGCAACTAAGGAGTGAAATAGAAAGGGGGGGAA
154 TTAAATTAAAATAGGAAAAACTGTGATGGCAACTAAGGAGTGAAATAGAAAGGGGGGGAA
30 TTAAATTAAAATAGGAAAAACTGTGATGGCAACTAAGGAGTGAAATAGAAAGGGGGAGAA
39 TTAAATTAAAATAGGAAAAACTGTGATGGCAACTAAGGAGTGAAATAGAAAGGGGGAGAA
237 TTAAATTAAAATAGGAAAAACTGTGATGGCAACTAAGGAGTGAAATAGAAAGGGGGGGAA
65 TTAAATTAAAATAGGAAAAACTGTGATGGCAACTAAGGAGTGAAATAGAAAGGGGGGGAA
44 TTAAATTAAAATAGGAAAAACTGTGATGGCAACTAAGGAGTGAAATAGAAAGGGGGAGAA
168 TTAAATTAAAATAGGAAAAACTGTGATGGCGACTAAGGAGTGAAATAGAAAAGGGGGGAA
95 TTAAATTAAAATAGGAAAAACTGTGATGGCAACTAAGGAGTGAAATAGAAAGGGGGAAAA
22 TTAAATTAAAATAGGAAAAACTGTGATGGCAACTAAGGAGTGAAATAGAAAAGGGGGGAA
79 TTAAATTAAAATAGGAAAAACTGTGATGGCAACTAAGGAGTGAAATAGAAAAGGGGGGAA
211 TTAAATTAAAATAGGAAAAACTGTGATGGCAACTAAGGAGTGAAATAGAAAAGGGGGGAA
88 TTAAATTAAAATAGGAAAAACTGTGATGGCAACTAAGGAGTGAAATAGAAAAGGGGAGAA
184 TTAAATTAAAATAGGAAAAACTGTGATGGCAACTAAGGAGTGAAATAGAAAAGGGGAGAA
78 TTAAATTAAAATAGGAAAAACTGTGATGGCAACTAAGGAGTGAAATAGAAAAGGGGAGAA
71 TTAAATTAAAATAGGAAAAACTGTGATGGCAACTAAGGAGTGAAATAGAAAGGGGGGGAA
196 TTAAATTAAAATAGGAAAAACTGTGATGGCAACTAAGGAGTGAAATAGAAAGGGGGGGAA
43 TTAAATTAAAATAGGAAAAACTGTGATGGCAACTAAGGAGTGAAATAGAAAGGGGGGGAA

151	TTAAATTAAAATAGGAAAAACTGTGATGGCAACTAAGGAGTGAAATAGAAAGGGGGGGAA
27	TTAAATTAAAATAGGAAAAACTGTGATGGCAACTAAGGAGTGAAATAGAAAGGGGGAGAA
197	TTAAATTAAAATAGGAAAAACTGTGATGGCAACTAAGGAGTGAAATAGAAAAGGGGAGAA
109	TTAAATTAAAATAGGAAAAACTGTGATGGCAACTAAGGAGTGAAATAGAAAGGGGGAGAA
169	TTAAATTAAAATAGGAAAAACTGTGATGGCAACTAAGGAGTGAAATAGAAAGGGGGAGAA
94	TTAAATTAAAATAGGAAAAACTGTGATGGCAACTAAGGAGTGAAATAGAAAGGGGGAGAA
232	TTAAATTAAAATAGGAAAAACTGTGATGGCAACTAAGGAGTGAAATAGAAAGGGGGAGAA
222	TTAAATTAAAATAGGAAAAACTGTGATGGCAACTAAGGAGTGAAATAGAAAGGGGGAAAA
24	TTAAATTAAAATAGGAAAAACTGTGATGGCAACTAAGGAGTGAAATAGAAAGGGGGAAAA
15	TTAAATTAAAATAGGAAAAACTGTGATGGCAACTAAGGAGTGAAATAGAAAGGGGGAAAA
13	TTAAATTAAAATAGGAAAAACTGTGATGGCAACTAAGGAGTGAAATAGAAAGGGGGAAAA
14	TTAAATTAAAATAGGAAAAACTGTGATGGCAACTAAGGAGTGAAATAGAAAGGGGGAGAA
35	TTAAATTAAAATAGGAAAAACTGTGATGGCAACTAAGGAGTGAAATAGAAAGGGGGAGAA
111	TTAAATTAAAATAGGAAAAACTGTGATGGCAACTAAGGAGTGAAATAGAAAGGGGGAGAA
157	TTAAATTAAAATAGGAAAAACTGTGATGGCAACTAAGGAGTGAAATAGAAAGGGGGAGAA
31	TTAAATTAAAATAGGAAAAACTGTGATGGCAACTAAGGAGTGAAATAGAAAGGGGGAGAA
47	TTAAATTAAAATAGGAAAAACTGTGATGGCAACTAAGGAGTGAAATAGAAAGGGGGAGAA
33	TTAAATTAAAATAGGAAAAACTGTGATGGCAACTAAGGAGTGAAATAGAAAGGGGGAGAA
51	TTAAATTAAAATAGGAAAAACTGTGATGGCAACTAAGGAGTGAAATAGAAAGGGGGAGAA
85	TTAAATTAAAATAGGAAAAACTGTGATGGCAACTAAGGAGTGAAATAGAAAGGGGGAGAA
54	TTAAATTAAAATAGGAAAAACTGTGATGGCAACTAAGGAGTGAAATAGAAAGGGGGAGAA
96	TTAAATTAAAATAGGAAAAACTGTGATGGCAACTAAGGAGTGAAATAGAAAGGGGGAGAA
61	TTAAATTAAAATAGGAAAAACTGTGATGGCAACTAAGGAGTGAAATAGAAAGGGGGAGAA
80	TTAAATTAAAATAGGAAAAACTGTGATGGCAACTAAGGAGTGAAATAGAAAGGGGGAGAA
82	TTAAATTAAAATAGGAAAAACTGTGATGGCAACTAAGGAGTGAAATAGAAAGGGGGAGAA
129	TTAAATTAAAATAGGAAAAACTGTGATGGCAACTAAGGAGTGAAATAGAAAGGGGGAGAA
46	TTAAATTAAAATAGGAAAAACTGTGATGGCAACTAAGGAGTGAAATAGAAAGGGGGAGAA
74	TTAAATTAAAATAGGAAAAACTGTGATGGCAACTAAGGAGTGAAATAGAAAGGGGGAGAA
101	TTAAATTAAAATAGGAAAAACTGTGATGGCAACTAAGGAGTGAAATAGAAAGGGGGAGAA
66	TTAAATTAAAATAGGAAAAACTGTGATGGCAACTAAGGAGTGAAATAGAAAGGGGGAGAA
104	TTAAATTAAAATAGGAAAAACTGTGATGGCAACTAAGGAGTGAAATAGAAAGGGGGAGAA
118	TTAAATTAAAATAGGAAAAACTGTGATGGCAACTAAGGAGTGAAATAGAAAGGGGGAGAA
21	TTAAATTAAAATAGGAAAAACTGTGATGGCAACTAAGGAGTGAAATAGAAAGGGGGAGAA
77	TTAAATTAAAATAGGAAAAACTGTGATGGCAACTAAGGAGTGAAATAGAAAGGGGGAGAA
124	TTAAATTAAAATAGGAAAAACTGTGATGGCAACTAAGGAGTGAAATAGAAAGGGGGAGAA
20	TTAAATTAAAATAGGAAAAACTGTGATGGCAACTAAGGAGTGAAATAGAAAGGGGGAGAA
110	TTAAATTAAAATAGGAAAAACTGTGATGGCAACTAAGGAGTGAAATAGAAAGGGGGAGAA
70	TTAAATTAAAATAGGAAAAACTGTGATGGCAACTAAGGAGTGAAATAGAAAGGGGGAGAA
8	TTAAATTAAAATAGGAAAAACTGTGATGGCAACTAAGGAGTGAAATAGAAAGGGGGAGAA
55	TTAAATTAAAATAGGAAAAACTGTGATGGCAACTAAGGAGTGAAATAGAAAGGGGGAGAA
64	TTAAATTAAAATAGGAAAAACTGTGATGGCAACTAAGGAGTGAAATAGAAAGGGGGAGAA
93	TTAAATTAAAATAGGAAAAACTGTGATGGCAACTAAGGAGTGAAATAGAAAGGGGGAGAA
202	TTAAATTAAAATAGGAAAAACTGTGATGGCAACTAAGGAGTGAAATAGAAAGGGGGAGAA
10	TTAAATTAAAATAGGAAAAACTGTGATGGCAACTAAGGAGTGAAATAGAAAGGGGGAGAA
16	TTAAATTAAAATAGGAAAAACTGTGATGGCAACTAAGGAGTGAAATAGAAAGGGGGAGAA
76	TTAAATTAAAATAGGAAAAACTGTGATGGCAACTAAGGAGTGAAATAGAAAGGGGGAGAA
116	TTAAATTAAAATAGGAAAAACTGTGATGGCAACTAAGGAGTGAAATAGAAAGGGGGAGAA
220	TTAAATTAAAATAGGAAAAACTGTGATGGCAACTAAGGAGTGAAATAGAAAGGGGGAGAA
234	TTAAATTAAAATAGGAAAAACTGTGATGGCAACTAAGGAGTGAAATAGAAAGGGGGAGAA
2	TTAAATTAAAATAGGAAAAACTGTGATGGCAACTAAGGAGTGAAATAGAAAGGGGGAGAA
38	TTAAATTAAAATAGGAAAAACTGTGATGGCAACTAAGGAGTGAAATAGAAAGGGGGAGAA
73	TTAAATTAAAATAGGAAAAACTGTGATGGCAACTAAGGAGTGAAATAGAAAGGGGGAGAA
87	TTAAATTAAAATAGGAAAAACTGTGATGGCAACTAAGGAGTGAAATAGAAAGGGGGAGAA
92	TTAAATTAAAATAGGAAAAACTGTGATGGCAACTAAGGAGTGAAATAGAAAGGGGGAGAA
98	TTAAATTAAAATAGGAAAAACTGTGATGGCAACTAAGGAGTGAAATAGAAAGGGGGAGAA
137	TTAAATTAAAATAGGAAAAACTGTGATGGCAACTAAGGAGTGAAATAGAAAGGGGGAGAA
19	TTAAATTAAAATAGGAAAAACTGTGATGGCAACTAAGGAGTGAAATAGAAAGGGGGAGAA

171	TTAAATTAAAATAGGAAAAACTGTGATGGCAACTAAGGAGTGAAATAGAAAGGGGGAGAA
205	TTAAATTAAAATAGGAAAAACTGTGATGGCAACTAAGGAGTGAAATAGAAAGGGGGAGAA
75	TTAAATTAAAATAGGAAAAACTGTGATGGCAACTAAGGAGTGAAATAGAAAGGGGGAGAA
62	TTAAATTAAAATAGGAAAAACTGTGATGGCAACTAAGGAGTGAAATAGAAAGGGGGAGAA
4	TTAAATTAAAATAGGAAAAACTGTGATGGCAACTAAGGAGTGAAATAGAAAGGGGGAGAA
7	TTAAATTAAAATAGGAAAAACTGTGATGGCAACTAAGGAGTGAAATAGAAAGGGGGAGAA
25	TTAAATTAAAATAGGAAAAACTGTGATGGCAACTAAGGAGTGAAATAGAAAGGGGGAGAA
28	TTAAATTAAAATAGGAAAAACTGTGATGGCAACTAAGGAGTGAAATAGAAAGGGGGAGAA
36	TTAAATTAAAATAGGAAAAACTGTGATGGCAACTAAGGAGTGAAATAGAAAGGGGGAGAA
49	TTAAATTAAAATAGGAAAAACTGTGATGGCAACTAAGGAGTGAAATAGAAAGGGGGAGAA
145	TTAAATTAAAATAGGAAAAACTGTGATGGCAACTAAGGAGTGAAATAGAAAGGGGGAGAA
181	TTAAATTAAAATAGGAAAAACTGTGATGGCAACTAAGGAGTGAAATAGAAAGGGGGAGAA
188	TTAAATTAAAATAGGAAAAACTGTGATGGCAACTAAGGAGTGAAATAGAAAGGGGGAGAA
59	TTAAATTAAAATAGGAAAAACTGTGATGGCAACTAAGGAGTGAAATAGAAAGGGGGAGAA
121	TTAAATTAAAATAGGAAAAACTGTGATGGCAACTAAGGAGTGAAATAGAAAGGGGGAGAA
127	TTAAATTAAAATAGGAAAAACTGTGATGGCAACTAAGGAGTGAAATAGAAAGGGGGAGAA
135	TTAAATTAAAATAGGAAAAACTGTGATGGCAACTAAGGAGTGAAATAGAAAGGGGGAGAA
136	TTAAATTAAAATAGGAAAAACTGTGATGGCAACTAAGGAGTGAAATAGAAAGGGGGAGAA
29	TTAAATTAAAATAGGAAAAACTGTGATGGCAACTAAGGAGTGAAATAGAAAGGGGGAGAA
89	TTAAATTAAAATAGGAAAAACTGTGATGGCAACTAAGGAGTGAAATAGAAAGGGGGAGAA
216	TTAAATTAAAATAGGAAAAACTGTGATGGCAACTAAGGAGTGAAATAGAAAGGGGGAGAA
164	TTAAATTAAAATAGGAAAAACTGTGATGGCAACTAAGGAGTGAAATAGAAAGGGGGAGAA
63	TTAAATTAAAATAGGAAAAACTGTGATGGCAACTAAGGAGTGAAATAGAAAGGGGGAGAA
161	TTAAATTAAAATAGGAAAAACTGTGATGGCAACTAAGGAGTGAAATAGAAAGGGGGAGAA
183	TTAAATTAAAATAGGAAAAACTGTGATGGCAACTAAGGAGTGAAATAGAAAGGGGGAGAA
18	TTAAATTAAAATAGGAAAAACTGTGATGGCAACTAAGGAGTGAAATAGAAAGGGGGAAAA
90	TTAAATTAAAATAGGAAAAACTGTGATGGCAACTAAGGAGTGAAATAGAAAAGGGGAGAA
162	TTAAATTAAAATAGGAAAAACTGTGATGGCAACTAAGGAGTGAAATAGAAAGGGGGAGAA
177	TTAAATTAAAATAGGAAAAACTGTGATGGCAACTAAGGAGTGAAATAGAAAGGGGGGGAA
97	TTAAATTAAAATAGGAAAAACTGTGATGGCAACTAAGGAGTGAAATAGAAAGGGGGAGAA
231	TTAAATTAAAATAGGAAAAACTGTGATGGCAACTAAGGAGTGAAATAGAAAGGGGGAGAA
143	TTAAATTAAAATAGGAAAAACTGTGATGGCAACTAAGGAGTGAAATAGAAAGGGGGAGAA
11	TTAAATTAAAATAGGAAAAACTGTGATGGCAACTAAGGAGTGAAATAGAAAGGGGGGGAA
153	TTAAATTAAAATAGGAAAAACTGTGATGGCAACTAAGGAGTGAAATAGAAAGGGGGGGAA
138	TTAAATTAAAATAGGAAAAACTGTGATGGCAACTAAGGAGTGAAATAGAAAGGGGGAGAA
185	TTAAATTAAAATAGGAAAAACTGTGATGGCAACTAAGGAGTGAAATAGAAAGGGGGAGAA
194	TTAAATTAAAATAGGAAAAACTGTGATGGCAACTAAGGAGTGAAATAGAAAGGGGGAGAA
236	TTAAATTAAAATAGGAAAAACTGTGATGGCAACTAAGGAGTGAAATAGAAAGGGGGAGAA
32	TTAAATTAAAATAGGAAAAACTGTGATGGCAACTAAGGAGTGAAATAGAAAGGGGGAGAA
52	TTAAATTAAAATAGGAAAAACTGTGATGGCAACTAAGGAGTGAAATAGAAAGGGGGAGAA
60	TTAAATTAAAATAGGAAAAACTGTGATGGCAACTAAGGAGTGAAATAGAAAGGGGGAGAA
72	TTAAATTAAAATAGGAAAAACTGTGATGGCAACTAAGGAGTGAAATAGAAAGGGGGAGAA
112	TTAAATTAAAATAGGAAAAACTGTGATGGCAACTAAGGAGTGAAATAGAAAGGGGGAGAA
141	TTAAATTAAAATAGGAAAAACTGTGATGGCAACTAAGGAGTGAAATAGAAAGGGGGAGAA
155	TTAAATTAAAATAGGAAAAACTGTGATGGCAACTAAGGAGTGAAATAGAAAGGGGGAGAA
1	TTAAATTAAAATAGGAAAAACTGTGATGGCAACTAAGGAGTGAAATAGAAAGGGGGAGAA
6	TTAAATTAAAATAGGAAAAACTGTGATGGCAACTAAGGAGTGAAATAGAAAGGGGGAGAA
58	TTAAATTAAAATAGGAAAAACTGTGATGGCAACTAAGGAGTGAAATAGAAAGGGGGAGAA
68	TTAAATTAAAATAGGAAAAACTGTGATGGCAACTAAGGAGTGAAATAGAAAGGGGGAGAA
180	TTAAATTAAAATAGGAAAAACTGTGATGGCAACTAAGGAGTGAAATAGAAAGGGGGAGAA
81	TTAAATTAAAATAGGAAAAACTGTGATGGCAACTAAGGAGTGAAATAGAAAGGGGGAGAA
103	TTAAATTAAAATAGGAAAAACTGTGATGGCAACTAAGGAGTGAAATAGAAAGGGGGAGAA
113	TTAAATTAAAATAGGAAAAACTGTGATGGCAACTAAGGAGTGAAATAGAAAGGGGGAGAA
163	TTAAATTAAAATAGGAAAAACTGTGATGGCAACTAAGGAGTGAAATAGAAAGGGGGAGAA
40	TTAAATTAAAATAGGAAAAACTGTGATGGCAACTAAGGAGTGAAATAGAAAGGGGGAAAA
56	TTAAATTAAAATAGGAAAAACTGTGATGGCAACTAAGGAGTGAAATAGAAAGGGGGAAAA
218	TTAAATTAAAATAGGAAAAACTGTGATGGCAACTAAGGAGTGAAATAGAAAGGGGGAAAA

```
41              TTAAATTAAAATAGGAAAAACTGTGATGGCAACTAAGGAGTGAAATAGAAAGGGGGAAAA
42              TTAAATTAAAATAGGAAAAACTGTGATGGCAACTAAGGAGTGAAATAGAAAGGGGGAGAA
160             TTAAATTAAAATAGGAAAAACTGTGATGGCAACTAAGGAGTGAAATAGAAAGGGGGAAAA
5               TTAAATTAAAATAGGAAAAACTGTGATGGCAACTAAGGAGTGAAATAGAAAGGGGGAGAA
228             TTAAATTAAAATAGGAAAAACTGTGATGGCAACTAAGGAGTGAAATAGAAAGGGGGAGAA
17              TTAAATTAAAATAGGAAAAACTGTGATGGCAACTAAGGAGTGAAATAGAAAGGGGGAGAA
37              TTAAATTAAAATAGGAAAAACTGTGATGGCAACTAAGGAGTGAAATAGAAAGGGGGAGAA
91              TTAAATTAAAATAGGAAAAACTGTGATGGCAACTAAGGAGTGAAATAGAAAGGGGGAAAA
140             TTAAATTAAAATAGGAAAAACTGTGATGGCAACTAAGGAGTGAAATAGAAAAGGGGGGAA
167             TTAAATTAAAATAGGAAAAACTGTGATGGCAACTAAGGAGTGAAATAGAAAAGGGGAGAA
                ****************************** ******************** ****  **

172             AGAGTTAATTATCGTTGACTAGTAAATGTACCACAAGTTTTTCTTCAAGTTCCTTCCCTC
229             AGAGTTAATTATCGTTGACTAGTAAATGTACCACAAGTTTTTCTTCAAGTTCCTTCCCTC
208             AGAGTTAATTATCGTTGACTAGTAAATGTACCACAAGTTTTTCTTCAAGTTCCTTCCCTC
83              AGAGTTAATTATCGTTGACTAATAAATGTACCCCAAGTTTTTCTTCAAGTTCCTTCCCTC
3               AGAGTTAATTATCGTTGACTAATAAATGTACCCCAAGTTTTTCTTCAAGTTCCTTCCCTC
120             AGAGTTAATTATCGTTGACTAGTAAATGTACCCCAAGTTTTTCTTCAAGTTCCTTCCCTC
99              AGAGTTAATTATCGTTGACTAGTAAATGTACCCCAAGTTTTTCTTCAAGTTCCTTCCCTC
126             AGAGTTAATTATCGTTGACTAGTAAATGTACCCCAAGTTTTTCTTCAAGTTCCTTCCCTC
173             AGAGTTAATTATCGTTGACTAGTAAATGTACCCCAAGTTTTTCTTCAAGTTCCTTCCCTC
204             AGAGTTAATTATCGTTGACTAGTAAATGTACCCCAAGTTTTTCTTCAAGTTCCTTCCCTC
239             AGAGTTAATTATCGTTGACTAGTAAATGTACCCCAAGTTTTTCTTCAAGTTCCTTCCCTC
117             AGAGTTAATTATCGTTGACTAGTAAATGTACCCCAAGTTTTTCTTCAAGTTCCTTCCCTC
123             AGAGTTAATTATCGTTGACTAGTAAATGTACCCCAAGTTTTTCTTCAAGTTCCTTCCCTC
210             AGAGTTAATTATCGTTGACTAGTAAATGTACCCCAAGTTTTTCTTCAAGTTCCTTCCCTC
86              AGAGTTAATTATCGTTGACTAGTAAATGTACCCCAAGTTTTTCTTCAAGTTCCTTCCCTC
100             AGAGTTAATTATCGTTGACTAGTAAATGTACCCCAAGTTTTTCTTCAAGTTCCTTCCCTC
114             AGAGTTAATTATCGTTGACTAGTAAATGTACCCCAAGTTTTTCTTCAAGTTCCTTCCCTC
170             AGAGTTAATTATCGTTGACTAGTAAATGTACCCCAAGTTTTTCTTCAAGTTCCTTCCCTC
108             AGAGTTAATTATCGTTGACTAGTAAATGTACCCCAAGTTTTTCTTCAAGTTCCTTCCCTC
174             AGAGTTAATTATCGTTGACTAGTAAATGTACCCCAAGTTTTTCTTCAAGTTCCTTCCCTC
158             AGAGTTAATTATCGTTGACTAGTAAATGTACCCCAAGTTTTTCTTCAAGTTCCTTCCCTC
154             AGAGTTAATTATCGTTGACTAGTAAATGTACCCCAAGTTTTTCTTCAAGTTCCTTCCCTC
30              AGAGTTAATTATCGTTGACTAGTAAATGTACCCCAAGTTTTTCTTCAAGTTCCTTCCCTC
39              AGAGTTAATTATCGTTGACTAGTAAATGTACCCCAAGTTTTTCTTCAAGTTCCTTCCCTC
237             AGAGTTAATTATCGTTGACTAGTAAATGTACCCCAAGTTTTTCTTCAAGTTCCTTCCCTC
65              AGAGTTAATTATCGTTGACTAGTAAATGTACCCCAAGTTTTTCTTCAAGTTCCTTCCCTC
44              AGAGTTAATTATCGTTGACTAGTAAATGTACCCCAAGTTTTTCTTCAAGTTCCTTCCCTC
168             AGAGTTAATTATCGTTGACTAGTAAATGTACCCCAAGTTTTTCTTCAAGTTCCTTCCCTC
95              AGAGTTAATTATCGTTGACTAGTAAATGTACCCCAAGTTTTTCTTCAAGTTCCTTCCCTC
22              AGAGTTAATTATCGTTGACTAATAAATGTACCCCAAGTTTTTCTTCAAGTTCCTTCCCTC
79              AGAGTTAATTATCGTTGACTAGTAAATGTACCCCAAGTTTTTCTTCAAGTTCCTTCCCTC
211             AGAGTTAATTATCGTTGACTAGTAAATGTACCCCAAGTTTTTCTTCAAGTTCCTTCCCTC
88              AGAGTTAATTATCGTTGACTAGTAAATGTACCCCAAGTTTTTCTTCAAGTTCCTTCCCTC
184             AGAGTTAATTATCGTTGACTAGTAAATGTACCCCAAGTTTTTCTTCAAGTTCCTTCCCTC
78              AGAGTTAATTATCGTTGACTAGTAAATGTACCCCAAGTTTTTCTTCAAGTTCCTTCCCTC
71              AGAGTTAATTATCGTTGACTAGTAAATGTACCCCAAGTTTTTCTTCAAGTTCCTTCCCTC
196             AGAGTTAATTATCGTTGACTAGTAAATGTACCCCAAGTTTTTCTTCAAGTTCCTTCCCTC
43              AGAGTTAATTATCGTTGACTAGTAAATGTACCCCAAGTTTTTCTTCAAGTTCCTTCCCTC
151             AGAGTTAATTATCGTTGACTAGTAAATGTACCCCAAGTTTTTCTTCAAGTTCCTTCCCTC
27              AGAGTTAATTATCGTTGACTAGTAAATGTACCCCAAGTTTTTCTTCAAGTTCCTTCCCTC
197             AGAGTTAATTATCGTTGACTAGTAAATGTACCCCAAGTTTTTCTTCAAGTTCCTTCCCTC
109             AGAGTTAATTATCGTTGACTAGTAAATGTACCCCAAGTTTTTCTTCAAGTTCCTTCCCTC
169             AGAGTTAATTATCGTTGACTAGTAAATGTACCACAAGTTTTTCTTCAAGTTCCTTCCCTC
94              AGAGTTAATTATCGTTGACTAGTAAATGTACCCCAAGTTTTTCTTCAAGTTCCTTCCCTC
232             AGAGTTAATTATCGTTGACTAGTAAATGTACCCCAAGTTTTTCTTCAAGTTCCTTCCCTC
```

222	AGAGTTAATTATCGTTGACTAGTAAATGTACCACAAGTTTTTCTTCAAGTTCCTTCCCTC
24	AGAGTTAATTATCGTTGACTAGTAAATGTACCACAAGTTTTTCTTCAAGTTCCTTCCCTC
15	AGAGTTAATTATCGTTGACTAGTAAATGTACCACAAGTTTTTCTTCAAGTTCCTTCCCTC
13	AGAGTTAATTATCGTTGACTAGTAAATGTACCACAAGTTTTTCTTCAAGTTCCTTCCCTC
14	AGAGTTAATTATCGTTGACTAGTAAATGTACCCCAAGTTTTTCTTCAAGTTCCTTCCCTC
35	AGAGTTAATTATCGTTGACTAGTAAATGTACCCCAAGTTTTTCTTCAAGTTCCTTCCCTC
111	AGAGTTAATTATCGTTGACTAGTAAATGTACCCCAAGTTTTTCTTCAAGTTCCTTCCCTC
157	AGAGTTAATTATCGTTGACTAGTAAATGTACCCCAAGTTTTTCTTCAAGTTCCTTCCCTC
31	AGAGTTAATTATCGTTGACTAGTAAATGTACCCCAAGTTTTTCTTCAAGTTCCTTCCCTC
47	AGAGTTAATTATCGTTGACTAGTAAATGTACCCCAAGTTTTTCTTCAAGTTCCTTCCCTC
33	AGAGTTAATTATCGTTGACTAGTAAATGTACCCCAAGTTTTTCTTCAAGTTCCTTCCCTC
51	AGAGTTAATTATCGTTGACTAGTAAATGTACCCCAAGTTTTTCTTCAAGTTCCTTCCCTC
85	AGAGTTAATTATCGTTGACTAGTAAATGTACCCCAAGTTTTTCTTCAAGTTCCTTCCCTC
54	AGAGTTAATTATCGTTGACTAGTAAATGTACCCCAAGTTTTTCTTCAAGTTCCTTCCCTC
96	AGAGTTAATTATCGTTGACTAGTAAATGTACCCCAAGTTTTTCTTCAAGTTCCTTCCCTC
61	AGAGTTAATTATCGTTGACTAGTAAATGTACCCCAAGTTTTTCTTCAAGTTCCTTCCCTC
80	AGAGTTAATTATCGTTGACTAGTAAATGTACCCCAAGTTTTTCTTCAAGTTCCTTCCCTC
82	AGAGTTAATTATCGTTGACTAGTAAATGTACCCCAAGTTTTTCTTCAAGTTCCTTCCCTC
129	AGAGTTAATTATCGTTGACTAGTAAATGTACCCCAAGTTTTTCTTCAAGTTCCTTCCCTC
46	AGAGTTAATTATCGTTGACTAGTAAATGTACCCCAAGTTTTTCTTCAAGTTCCTTCCCTC
74	AGAGTTAATTATCGTTGACTAGTAAATGTACCCCAAGTTTTTCTTCAAGTTCCTTCCCTC
101	AGAGTTAATTATCGTTGACTAGTAAATGTACCCCAAGTTTTTCTTCAAGTTCCTTCCCTC
66	AGAGTTAATTATCGTTGACTAGTAAATGTACCCCAAGTTTTTCTTCAAGTTCCTTCCCTC
104	AGAGTTAATTATCGTTGACTAGTAAATGTACCCCAAGTTTTTCTTCAAGTTCCTTCCCTC
118	AGAGTTAATTATCGTTGACTAGTAAATGTACCCCAAGTTTTTCTTCAAGTTCCTTCCCTC
21	AGAGTTAATTATCGTTGACTAGTAAATGTACCCCAAGTTTTTCTTCAAGTTCCTTCCCTC
77	AGAGTTAATTATCGTTGACTAGTAAATGTACCCCAAGTTTTTCTTCAAGTTCCTTCCCTC
124	AGAGTTAATTATCGTTGACTAGTAAATGTACCCCAAGTTTTTCTTCAAGTTCCTTCCCTC
20	AGAGTTAATTATCGTTGACTAGTAAATGTACCCCAAGTTTTTCTTCAAGTTCCTTCCCTC
110	AGAGTTAATTATCGTTGACTAGTAAATGTACCCCAAGTTTTTCTTCAAGTTCCTTCCCTC
70	AGAGTTAATTATCGTTGACTAGTAAATGTACCCCAAGTTTTTCTTCAAGTTCCTTCCCTC
8	AGAGTTAATTATCGTTGACTAGTAAATGTACCCCAAGTTTTTCTTCAAGTTCCTTCCCTC
55	AGAGTTAATTATCGTTGACTAGTAAATGTACCCCAAGTTTTTCTTCAAGTTCCTTCCCTC
64	AGAGTTAATTATCGTTGACTAGTAAATGTACCCCAAGTTTTTCTTCAAGTTCCTTCCCTC
93	AGAGTTAATTATCGTTGACTAGTAAATGTACCCCAAGTTTTTCTTCAAGTTCCTTCCCTC
202	AGAGTTAATTATCGTTGACTAGTAAATGTACCCCAAGTTTTTCTTCAAGTTCCTTCCCTC
10	AGAGTTAATTATCGTTGACTAGTAAATGTACCCCAAGTTTTTCTTCAAGTTCCTTCCCTC
16	AGAGTTAATTATCGTTGACTAGTAAATGTACCCCAAGTTTTTCTTCAAGTTCCTTCCCTC
76	AGAGTTAATTATCGTTGACTAGTAAATGTACCCCAAGTTTTTCTTCAAGTTCCTTCCCTC
116	AGAGTTAATTATCGTTGACTAGTAAATGTACCCCAAGTTTTTCTTCAAGTTCCTTCCCTC
220	AGAGTTAATTATCGTTGACTAGTAAATGTACCCCAAGTTTTTCTTCAAGTTCCTTCCCTC
234	AGAGTTAATTATCGTTGACTAGTAAATGTACCCCAAGTTTTTCTTCAAGTTCCTTCCCTC
2	AGAGTTAATTATCGTTGACTAGTAAATGTACCCCAAGTTTTTCTTCAAGTTCCTTCCCTC
38	AGAGTTAATTATCGTTGACTAGTAAATGTACCCCAAGTTTTTCTTCAAGTTCCTTCCCTC
73	AGAGTTAATTATCGTTGACTAGTAAATGTACCCCAAGTTTTTCTTCAAGTTCCTTCCCTC
87	AGAGTTAATTATCGTTGACTAGTAAATGTACCCCAAGTTTTTCTTCAAGTTCCTTCCCTC
92	AGAGTTAATTATCGTTGACTAGTAAATGTACCCCAAGTTTTTCTTCAAGTTCCTTCCCTC
98	AGAGTTAATTATCGTTGACTAGTAAATGTACCCCAAGTTTTTCTTCAAGTTCCTTCCCTC
137	AGAGTTAATTATCGTTGACTAGTAAATGTACCCCAAGTTTTTCTTCAAGTTCCTTCCCTC
19	AGAGTTAATTATCGTTGACTAGTAAATGTACCCCAAGTTTTTCTTCAAGTTCCTTCCCTC
171	AGAGTTAATTATCGTTGACTAGTAAATGTACCCCAAGTTTTTCTTCAAGTTCCTTCCCTC
205	AGAGTTAATTATCGTTGACTAGTAAATGTACCCCAAGTTTTTCTTCAAGTTCCTTCCCTC
75	AGAGTTAATTATCGTTGACTAGTAAATGTACCCCAAGTTTTTCTTCAAGTTCCTTCCCTC
62	AGAGTTAATTATCGTTGACTAGTAAATGTACCCCAAGTTTTTCTTCAAGTTCCTTCCCTC
4	AGAGTTAATTATCGTTGACTAGTAAATGTACCCCAAGTTTTTCTTCAAGTTCCTTCCCTC
7	AGAGTTAATTATCGTTGACTAGTAAATGTACCCCAAGTTTTTCTTCAAGTTCCTTCCCTC
25	AGAGTTAATTATCGTTGACTAGTAAATGTACCCCAAGTTTTTCTTCAAGTTCCTTCCCTC

28	AGAGTTAATTATCGTTGACTAGTAAATGTACCCCAAGTTTTTCTTCAAGTTCCTTCCCTC
36	AGAGTTAATTATCGTTGACTAGTAAATGTACCCCAAGTTTTTCTTCAAGTTCCTTCCCTC
49	AGAGTTAATTATCGTTGACTAGTAAATGTACCCCAAGTTTTTCTTCAAGTTCCTTCCCTC
145	AGAGTTAATTATCGTTGACTAGTAAATGTACCCCAAGTTTTTCTTCAAGTTCCTTCCCTC
181	AGAGTTAATTATCGTTGACTAGTAAATGTACCCCAAGTTTTTCTTCAAGTTCCTTCCCTC
188	AGAGTTAATTATCGTTGACTAGTAAATGTACCCCAAGTTTTTCTTCAAGTTCCTTCCCTC
59	AGAGTTAATTATCGTTGACTAGTAAATGTACCCCAAGTTTTTCTTCAAGTTCCTTCCCTC
121	AGAGTTAATTATCGTTGACTAGTAAATGTACCCCAAGTTTTTCTTCAAGTTCCTTCCCTC
127	AGAGTTAATTATCGTTGACTAGTAAATGTACCCCAAGTTTTTCTTCAAGTTCCTTCCCTC
135	AGAGTTAATTATCGTTGACTAGTAAATGTACCCCAAGTTTTTCTTCAAGTTCCTTCCCTC
136	AGAGTTAATTATCGTTGACTAGTAAATGTACCCCAAGTTTTTCTTCAAGTTCCTTCCCTC
29	AGAGTTAATTATCGTTGACTAGTAAATGTACCCCAAGTTTTTCTTCAAGTTCCTTCCCTC
89	AGAGTTAATTATCGTTGACTAGTAAATGTACCCCAAGTTTTTCTTCAAGTTCCTTCCCTC
216	AGAGTTAATTATCGTTGACTAGTAAATGTACCCCAAGTTTTTCTTCAAGTTCCTTCCCTC
164	AGAGTTAATTATCGTTGACTAGTAAATGTACCCCAAGTTTTTCTTCAAGTTCCTTCCCTC
63	AGAGTTAATTATCGTTGACTAGTAAATGTACCCCAAGTTTTTCTTCAAGTTCCTTCCCTC
161	AGAGTTAATTATCGTTGACTAGTAAATGTACCCCAAGTTTTTCTTCAAGTTCCTTCCCTC
183	AGAGTTAATTATCGTTGACTAGTAAATGTACCCCAAGTTTTTCTTCAAGTTCCTTCCCTC
18	AGAGTTAATTATCGTTGACTAGTAAATGTACCCCAAGTTTTTCTTCAAGTTCCTTCCCTC
90	AGAGTTAATTATCGTTGACTAGTAAATGTACCACAAGTTTTTCTTCAAGTTCCTTCCCTC
162	AGAGTTAATTATCGTTGACTAGTAAATGTACCACAAGTTTTTCTTCAAGTTCCTTCCCTC
177	AGAGTTAATTATCGTTGACTAGTAAATGTACCCCAAGTTTTTCTTCAAGTTCCTTCCCTC
97	AGAGTTAATTATCGTTGACTAGTAAATGTACCACAAGTTTTTCTTCAAGTTCCTTCCCTC
231	AGAGTTAATTATCGTTGACTAGTAAATGTACCACAAGTTTTTCTTCAAGTTCCTTCCCTC
143	AGAGTTAATTATCGTTGACTAGTAAATGTACCACAAGTTTTTCTTCAAGTTCCTTCCCTC
11	AGAGTTAATTATCGTTGACTAGTAAATGTACCCCAAGTTTTTCTTCAAGTTCCTTCCCTC
153	AGAGTTAATTATCGTTGACTAGTAAATGTACCCCAAGTTTTTCTTCAAGTTCCTTCCCTC
138	AGAGTTAATTATCGTTGACTAGTAAATGTACCCCAAGTTTTTCTTCAAGTTCCTTCCCTC
185	AGAGTTAATTATCGTTGACTAGTAAATGTACCCCAAGTTTTTCTTCAAGTTCCTTCCCTC
194	AGAGTTAATTATCGTTGACTAGTAAATGTACCCCAAGTTTTTCTTCAAGTTCCTTCCCTC
236	AGAGTTAATTATCGTTGACTAGTAAATGTACCCCAAGTTTTTCTTCAAGTTCCTTCCCTC
32	AGAGTTAATTATCGTTGACTAGTAAATGTACCCCAAGTTTTTCTTCAAGTTCCTTCCCTC
52	AGAGTTAATTATCGTTGACTAGTAAATGTACCCCAAGTTTTTCTTCAAGTTCCTTCCCTC
60	AGAGTTAATTATCGTTGACTAGTAAATGTACCCCAAGTTTTTCTTCAAGTTCCTTCCCTC
72	AGAGTTAATTATCGTTGACTAGTAAATGTACCCCAAGTTTTTCTTCAAGTTCCTTCCCTC
112	AGAGTTAATTATCGTTGACTAGTAAATGTACCCCAAGTTTTTCTTCAAGTTCCTTCCCTC
141	AGAGTTAATTATCGTTGACTAGTAAATGTACCCCAAGTTTTTCTTCAAGTTCCTTCCCTC
155	AGAGTTAATTATCGTTGACTAGTAAATGTACCCCAAGTTTTTCTTCAAGTTCCTTCCCTC
1	AGAGTTAATTATCGTTGACTAGTAAATGTACCCCAAGTTTTTCTTCAAGTTCCTTCCCTC
6	AGAGTTAATTATCGTTGACTAGTAAATGTACCCCAAGTTTTTCTTCAAGTTCCTTCCCTC
58	AGAGTTAATTATCGTTGACTAGTAAATGTACCCCAAGTTTTTCTTCAAGTTCCTTCCCTC
68	AGAGTTAATTATCGTTGACTAGTAAATGTACCCCAAGTTTTTCTTCAAGTTCCTTCCCTC
180	AGAGTTAATTATCGTTGACTAGTAAATGTACCCCAAGTTTTTCTTCAAGTTCCTTCCCTC
81	AGAGTTAATTATCGTTGACTAGTAAATGTACCCCAAGTTTTTCTTCAAGTTCCTTCCCTC
103	AGAGTTAATTATCGTTGACTAGTAAATGTACCCCAAGTTTTTCTTCAAGTTCCTTCCCTC
113	AGAGTTAATTATCGTTGACTAGTAAATGTACCCCAAGTTTTTCTTCAAGTTCCTTCCCTC
163	AGAGTTAATTATCGTTGACTAGTAAATGTACCACAAGTTTTTCTTCAAGTTCCTTCCCTC
40	AGAGTTAATTATCGTTGACTAGTAAATGTACCCCAAGTTTTTCTTCAAGTTCCTTCCCTC
56	AGAGTTAATTATCGTTGACTAGTAAATGTACCCCAAGTTTTTCTTCAAGTTCCTTCCCTC
218	AGAGTTAATTATCGTTGACTAGTAAATGTACCCCAAGTTTTTCTTCAAGTTCCTTCCCTC
41	AGAGTTAATTATCGTTGACTAGTAAATGTACCACAAGTTTTTCTTCAAGTTCCTTCCCTC
42	AGAGTTAATTATCGTTGACTAGTAAATGTACCCCAAGTTTTTCTTCAAGTTCCTTCCCTC
160	AGAGTTAATTATCGTTGACTAGTAAATGTACCCCAAGTTTTTCTTCAAGTTCCTTCCCTC
5	AGAGTTAATTATCGTTGACTAGTAAATGTACCCCAAGTTTTTCTTCAAGTTCCTTCCCTC
228	AGAGTTAATTATCGTTGACTAGTAAATGTACCCCAAGTTTTTCTTCAAGTTCCTTCCCTC
17	AGAGTTAATTATCGTTGACTAGTAAATGTACCCCAAGTTTTTCTTCAAGTTCCTTCCCTC
37	AGAGTTAATTATCGTTGACTAGTAAATGTACCCCAAGTTTTTCTTCAAGTTCCTTCCCTC

```
91              AGAGTTAATTATCGTTGACTAGTAAATGTACCCCAAGTTTTTCTTCAAGTTCCTTCCCTC
140             AGAGTTAATTATCGTTGACTAGTAAATGTACCCCAAGTTTTTCTTCAAGTTCCTTCCCTC
167             AGAGTTAATTATCGTTGACTAGTAAATGTACCCCAAGTTTTTCTTCAAGTTCCTTCCCTC
                ******************** ********** **************************

172             CACCAATTGTCACGGGGTTATTTCTGGACTAAATCCCTCAACATGGCTAGCCTCTTTCTC
229             CACCAATTGTCACGGGGTTATTTCTGGACTAAATCCCTCAACATGGCTAGCCTCTTTCTC
208             CACCAATTGTCACGGGGTTATTTCTGGACTAAATCCCTCAACATGGCTAGCCTCTTTCTC
83              CACCAATTGTCACAGGGTTATTTTCGGACTAAATCCCTCAACATGGCTAGTTTCTTTCTC
3               CACCAATTGTCACAGGGTTATTTTCGGACTAAATCCCTCAACATGGCTAGTTTCTTTCTC
120             CACCAATTGTCACAGGGCTAATTCTGGACTAAATCCCTCAACATGGCTAGCCTCTTTCTC
99              CACCAATTGTCACAGGGCTAATTCTGGACTAAATCCCTCAACATGGCTAGCCTCTTTCTC
126             CACCAATTGTCACGGGGCTAATTCTGGACTAAATCCCTCAACATGGCTAGCCTCTTTCTC
173             CACCAATTGTCACGGGGCTAATTCTGGACTAAATCCCTCAACATGGCTAGCCTCTTTCTC
204             CACCAATTGTCACAGGGCTAATTCTGGACTAAATCCCTCAACATGGCTAGCCTCTTTCTC
239             CACCAATTGTCACAGGGCTAATTCTGGACTAAATCCCTCAACATGGCTAGCCTCTTTCTC
117             CACCAATTGTCACAGGGCTAATTCTGGACTAAATCCCTCAACATGGCTAGCCTCTTTCTC
123             CACCAATTGTCACAGGGCTAATTCTGGACTAAATCCCTCAACATGGCTAGCCTCTTTCTC
210             CACCAATTGTCACGGGGCTAATTCTGGACTAAATCCCTCAACATGGCTAGCCTCTTTCTC
86              CACCAATTGTCACGGGGCTAATTCTGGACTAAATCCCTCAACATGGCTAGCCTCTTTCTC
100             CACCAATTGTCACGGGGCTAATTCTGGACTAAATCCCTCAACATGGCTAGCCTCTTTCTC
114             CACCAATTGTCACAGGGCTAATTCTGGACTAAATCCCTCAACATGGCTAGCCTCTTTCTC
170             CACCAATTGTCACAGGGCTAATTCTGGACTAAATCCCTCAACATGGCTAGCCTCTTTCTC
108             CACCAATTGTCACAGGGCTAATTCTGGACTAAATCCCTCAACATGGCTAGCCTCTTTCTC
174             CACCAATTGTCACAGGGCTAATTCTGGACTAAATCCCTCAACATGGCTAGCCTCTTTCTC
158             CACCAATTGTCACAGGGCTAATTTCGGACTAAATCCCTCAACATGGCTAGCCTCTTTCTC
154             CACCAATTGTCACAGGGCTAATTCTGGACTAAATCCCTCAACATGGCTAGCCTCTTTCTC
30              CACCAATTGTCACAGGGCTAATTCTGGACTAAATCCCTCAACATGGCTAGCCTCTTTCTC
39              CACCAATTGTCACAGGGCTAATTCTGGACTAAATCCCTCAACATGGCTAGCCTCTTTCTC
237             CACCAATTGTCACAGGGCTAATTCTGGACTAAATCCCTCAACATGGCTAGTTTCTTTCTC
65              CACCAATTGTCACAGGGCTAATTCTGGACTAAATCCCTCAACATGGCTAGTTTCTTTCTC
44              CACCAATTGTCACGGGGCTAATTCTGGACTAAATCCCTCAACATGGCTAGCCTCTTTCTC
168             CACCAATTGTCACAGGGCTAATTCTGGACTAAATCCCTCAACATGGCTAGCCTCTTTCTC
95              CACCAATTGTCACAGGGCTAATTCTGGACTAAATCCCTCAACATGGCTAGCCTCTTTCTC
22              CACCAATTGTCACAGGGCTAATTCTGGACTAAATCCCTCAACATGGCTAGCCTCTTTCTC
79              CACCAATTGTCACAGGGCTAATTCTGGACTAAATCCCTCAACATGGCTAGCCTCTTTCTC
211             CACCAATTGTCACAGGGCTAATTCTGGACTAAATCCCTCAACATGGCTAGCCTCTTTCTC
88              CACCAATTGTCACAGGGCTAATTCTGGACTAAATCCCTCAACATGGCTAGCCTCTTTCTC
184             CACCAATTGTCACAGGGCTAATTCTGGACTAAATCCCTCAACATGGCTAGCCTCTTTCTC
78              CACCAATTGTCACAGGGCTAATTCTGGACTAAATCCCTCAACATGGCTAGCCTCTTTCTC
71              CACCAATTGTCACAGGGCTAATTCTGGACTAAATCCCTCAACATGGCTAGCCTCTTTCTC
196             CACCAATTGTCACAGGGCTAATTCTGGACTAAATCCCTCAACATGGCTAGCCTCTTTCTC
43              CACCAATTGTCACAGGGCTAATTCTGGACTAAATCCCTCAACATGGCTAGCCTCTTTCTC
151             CACCAATTGTCACAGGGCTAATTCTGGACTAAATCCCTCAACATGGCTAGCCTCTTTCTC
27              CACCAATTGTCACAGGGCTAATTCTGGACTAAATCCCTCAACATGGCTAGCCTCTTTCTC
197             CACCAATTGTCACAGGGCTAATTCTGGACTAAATCCCTCAACATGGCTAGCCTCTTTCTC
109             CACCAATTGTCACAGGGCTAATTCTGGACTAAATCCCTCAACATGGCTAGCCTCTTTCTC
169             CACCAATTGTCACAGGGCTAATTCTGGACTAAATCCCTCAACATGGCTAGCCTCTTTCTC
94              CACCAATTGTCACAGGGCTAATTCTGGACTAAATCCCTCAACATGGCTAGCCTCTTTCTC
232             CACCAATTGTCACAGGGCTAATTCTGGACTAAATCCCTCAACATGGCTAGCCTCTTTCTC
222             CACCAATTGTCACAGGGCTAATTCTGGACTAAATCCCTCAACATGGCTAGCCTCTTTCTC
24              CACCAATTGTCACAGGGCTAATTCTGGACTAAATCCCTCAACATGGCTAGCCTCTTTCTC
15              CACCAATTGTCACAGGGCTAATTCTGGACTAAATCCCTCAACATGGCTAGCCTCTTTCTC
13              CACCAATTGTCACAGGGCTAATTCTGGACTAAATCCCTCAACATGGCTAGCCTCTTTCTC
14              CACCAATTGTCACAGGGCTAATTCTGGACTAAATCCCTCAACATGGCTAGCCTCTTTCTC
35              CACCAATTGTCACAGGGCTAATTCTGGACTAAATCCCTCAACATGGCTAGCCTCTTTCTC
111             CACCAATTGTCACAGGGCTAATTCTGGACTAAATCCCTCAACATGGCTAGCCTCTTTCTC
```

157	CACCAATTGTCACAGGGCTAATTCTGGACTAAATCCCTCAACATGGCTAGCCTCTTTCTC
31	CACCAATTGTCACAGGGCTAATTCTGGACTAAATCCCTCAACATGGCTAGCCTCTTTCTC
47	CACCAATTGTCACAGGGCTAATTCTGGACTAAATCCCTCAACATGGCTAGCCTCTTTCTC
33	CACCAATTGTCACAGGGCTAATTCTGGACTAAATCCCTCAACATGGCTAGCCTCTTTCTC
51	CACCAATTGTCACAGGGCTAATTCTGGACTAAATCCCTCAACATGGCTAGCCTCTTTCTC
85	CACCAATTGTCACAGGGCTAATTCTGGACTAAATCCCTCAACATGGCTAGCCTCTTTCTC
54	CACCAATTGTCACAGGGCTAATTCTGGACTAAATCCCTCAACATGGCTAGCCTCTTTCTC
96	CACCAATTGTCACAGGGCTAATTCTGGACTAAATCCCTCAACATGGCTAGCCTCTTTCTC
61	CACCAATTGTCACAGGGCTAATTCTGGACTAAATCCCTCAACATGGCTAGCCTCTTTCTC
80	CACCAATTGTCACAGGGCTAATTCTGGACTAAATCCCTCAACATGGCTAGCCTCTTTCTC
82	CACCAATTGTCACAGGGCTAATTCTGGACTAAATCCCTCAACATGGCTAGCCTCTTTCTC
129	CACCAATTGTCACAGGGCTAATTCTGGACTAAATCCCTCAACATGGCTAGCCTCTTTCTC
46	CACCAATTGTCACAGGGCTAATTCTGGACTAAATCCCTCAACATGGCTAGCCTCTTTCTC
74	CACCAATTGTCACAGGGCTAATTCTGGACTAAATCCCTCAACATGGCTAGCCTCTTTCTC
101	CACCAATTGTCACAGGGCTAATTCTGGACTAAATCCCTCAACATGGCTAGCCTCTTTCTC
66	CACCAATTGTCACAGGGCTAATTCTGGACTAAATCCCTCAACATGGCTAGCCTCTTTCTC
104	CACCAATTGTCACAGGGCTAATTCTGGACTAAATCCCTCAACATGGCTAGCCTCTTTCTC
118	CACCAATTGTCACAGGGCTAATTCTGGACTAAATCCCTCAACATGGCTAGCCTCTTTCTC
21	CACCAATTGTCACAGGGCTAATTCTGGACTAAATCCCTCAACATGGCTAGCCTCTTTCTC
77	CACCAATTGTCACAGGGCTAATTCTGGACTAAATCCCTCAACATGGCTAGCCTCTTTCTC
124	CACCAATTGTCACAGGGCTAATTCTGGACTAAATCCCTCAACATGGCTAGCCTCTTTCTC
20	CACCAATTGTCACAGGGCTAATTCTGGACTAAATCCCTCAACATGGCTAGCCTCTTTCTC
110	CACCAATTGTCACAGGGCTAATTCTGGACTAAATCCCTCAACATGGCTAGCCTCTTTCTC
70	CACCAATTGTCACAGGGCTAATTCTGGACTAAATCCCTCAACATGGCTAGCCTCTTTCTC
8	CACCAATTGTCACAGGGCTAATTCTGGACTAAATCCCTCAACATGGCTAGCCTCTTTCTC
55	CACCAATTGTCACAGGGCTAATTCTGGACTAAATCCCTCAACATGGCTAGCCTCTTTCTC
64	CACCAATTGTCACAGGGCTAATTCTGGACTAAATCCCTCAACATGGCTAGCCTCTTTCTC
93	CACCAATTGTCACAGGGCTAATTCTGGACTAAATCCCTCAACATGGCTAGCCTCTTTCTC
202	CACCAATTGTCACAGGGCTAATTCTGGACTAAATCCCTCAACATGGCTAGCCTCTTTCTC
10	CACCAATTGTCACAGGGCTAATTCTGGACTAAATCCCTCAACATGGCTAGCCTCTTTCTC
16	CACCAATTGTCACAGGGCTAATTCTGGACTAAATCCCTCAACATGGCTAGCCTCTTTCTC
76	CACCAATTGTCACAGGGCTAATTCTGGACTAAATCCCTCAACATGGCTAGCCTCTTTCTC
116	CACCAATTGTCACAGGGCTAATTCTGGACTAAATCCCTCAACATGGCTAGCCTCTTTCTC
220	CACCAATTGTCACAGGGCTAATTCTGGACTAAATCCCTCAACATGGCTAGCCTCTTTCTC
234	CACCAATTGTCACAGGGCTAATTCTGGACTAAATCCCTCAACATGGCTAGCCTCTTTCTC
2	CACCAATTGTCACAGGGCTAATTCTGGACTAAATCCCTCAACATGGCTAGCCTCTTTCTC
38	CACCAATTGTCACAGGGCTAATTCTGGACTAAATCCCTCAACATGGCTAGCCTCTTTCTC
73	CACCAATTGTCACAGGGCTAATTCTGGACTAAATCCCTCAACATGGCTAGCCTCTTTCTC
87	CACCAATTGTCACAGGGCTAATTCTGGACTAAATCCCTCAACATGGCTAGCCTCTTTCTC
92	CACCAATTGTCACAGGGCTAATTCTGGACTAAATCCCTCAACATGGCTAGCCTCTTTCTC
98	CACCAATTGTCACAGGGCTAATTCTGGACTAAATCCCTCAACATGGCTAGCCTCTTTCTC
137	CACCAATTGTCACAGGGCTAATTCTGGACTAAATCCCTCAACATGGCTAGCCTCTTTCTC
19	CACCAATTGTCACAGGGCTAATTCTGGACTAAATCCCTCAACATGGCTAGCCTCTTTCTC
171	CACCAATTGTCACAGGGCTAATTCTGGACTAAATCCCTCAACATGGCTAGCCTCTTTCTC
205	CACCAATTGTCACAGGGCTAATTCTGGACTAAATCCCTCAACATGGCTAGCCTCTTTCTC
75	CACCAATTGTCACAGGGCTAATTCTGGACTAAATCCCTCAACATGGCTAGCCTCTTTCTC
62	CACCAATTGTCACAGGGCTAATTCTGGACTAAATCCCTCAACATGGCTAGCCTCTTTCTC
4	CACCAATTGTCACAGGGCTAATTCTGGACTAAATCCCTCAACATGGCTAGCCTCTTTCTC
7	CACCAATTGTCACAGGGCTAATTCTGGACTAAATCCCTCAACATGGCTAGCCTCTTTCTC
25	CACCAATTGTCACAGGGCTAATTCTGGACTAAATCCCTCAACATGGCTAGCCTCTTTCTC
28	CACCAATTGTCACAGGGCTAATTCTGGACTAAATCCCTCAACATGGCTAGCCTCTTTCTC
36	CACCAATTGTCACAGGGCTAATTCTGGACTAAATCCCTCAACATGGCTAGCCTCTTTCTC
49	CACCAATTGTCACAGGGCTAATTCTGGACTAAATCCCTCAACATGGCTAGCCTCTTTCTC
145	CACCAATTGTCACAGGGCTAATTCTGGACTAAATCCCTCAACATGGCTAGCCTCTTTCTC
181	CACCAATTGTCACAGGGCTAATTCTGGACTAAATCCCTCAACATGGCTAGCCTCTTTCTC
188	CACCAATTGTCACAGGGCTAATTCTGGACTAAATCCCTCAACATGGCTAGCCTCTTTCTC
59	CACCAATTGTCACAGGGCTAATTCTGGACTAAATCCCTCAACATGGCTAGCCTCTTTCTC

```
121              CACCAATTGTCACAGGGCTAATTCTGGACTAAATCCCTCAACATGGCTAGCCTCTTTCTC
127              CACCAATTGTCACAGGGCTAATTCTGGACTAAATCCCTCAACATGGCTAGCCTCTTTCTC
135              CACCAATTGTCACAGGGCTAATTCTGGACTAAATCCCTCAACATGGCTAGCCTCTTTCTC
136              CACCAATTGTCACAGGGCTAATTCTGGACTAAATCCCTCAACATGGCTAGCCTCTTTCTC
29               CACCAATTGTCACAGGGCTAATTCTGGACTAAATCCCTCAACATGGCTAGCCTCTTTCTC
89               CACCAATTGTCACAGGGCTAATTCTGGACTAAATCCCTCAACATGGCTAGCCTCTTTCTC
216              CACCAATTGTCACAGGGCTAATTCTGGACTAAATCCCTCAACATGGCTAGCCTCTTTCTC
164              CACCAATTGTCACAGGGCTAATTCTGGACTAAATCCCTCAACATGGCTAGCCTCTTTCTC
63               CACCAATTGTCACAGGGCTAATTCTGGACTAAATCCCTCAACATGGCTAGCCTCTTTCTC
161              CACCAATTGTCACAGGGCTAATTCTGGACTAAATCCCTCAACATGGCTAGCCTCTTTCTC
183              CACCAATTGTCACAGGGCTAATTCTGGACTAAATCCCTCAACATGGCTAGCCTCTTTCTC
18               CACCAATTGTCACAGGGCTAATTCTGGACTAAATCCCTCAACATGGCTAGCCTCTTTCTC
90               CACCAATTGTCACAGGGCTAATTCTGGACTAAATCCCTCAACATGGCTAGCCTCTTTCTC
162              CACCAATTGTCACAGGGCTAATTCTGGACTAAATCCCTCAACATGGCTAGCCTCTTTCTC
177              CACCAATTGTCACAGGGCTAATTCTGGACTAAATCCCTCAACATGGCTAGCCTCTTTCTC
97               CACCAATTGTCACAGGGCTAATTCTGGACTAAATCCCTCAACATGGCTAGCCTCTTTCTC
231              CACCAATTGTCACAGGGCTAATTCTGGACTAAATCCCTCAACATGGCTAGCCTCTTTCTC
143              CACCAATTGTCACAGGGCTAATTCTGGACTAAATCCCTCAACATGGCTAGCCTCTTTCTC
11               CACCAATTGTCACAGGGCTAATTCTGGACTAAATCCCTCAACATGGCTAGCCTCTTTCTC
153              CACCAATTGTCACAGGGCTAATTCTGGACTAAATCCCTCAACATGGCTAGCCTCTTTCTC
138              CACCAATTGTCACAGGGCTAATTCTGGACTAAATCCCTCAACATGGCTAGCCTCTTTCTC
185              CACCAATTGTCACAGGGCTAATTCTGGACTAAATCCCTCAACATGGCTAGCCTCTTTCTC
194              CACCAATTGTCACAGGGCTAATTCTGGACTAAATCCCTCAACATGGCTAGCCTCTTTCTC
236              CACCAATTGTCACAGGGCTAATTCTGGACTAAATCCCTCAACATGGCTAGCCTCTTTCTC
32               CACCAATTGTCACAGGGCTAATTCTGGACTAAATCCCTCAACATGGCTAGCCTCTTTCTC
52               CACCAATTGTCACAGGGCTAATTCTGGACTAAATCCCTCAACATGGCTAGCCTCTTTCTC
60               CACCAATTGTCACAGGGCTAATTCTGGACTAAATCCCTCAACATGGCTAGCCTCTTTCTC
72               CACCAATTGTCACAGGGCTAATTCTGGACTAAATCCCTCAACATGGCTAGCCTCTTTCTC
112              CACCAATTGTCACAGGGCTAATTCTGGACTAAATCCCTCAACATGGCTAGCCTCTTTCTC
141              CACCAATTGTCACAGGGCTAATTCTGGACTAAATCCCTCAACATGGCTAGCCTCTTTCTC
155              CACCAATTGTCACAGGGCTAATTCTGGACTAAATCCCTCAACATGGCTAGCCTCTTTCTC
1                CACCAATTGTCACAGGGCTAATTCTGGACTAAATCCCTCAACATGGCTAGCCTCTTTCTC
6                CACCAATTGTCACAGGGCTAATTCTGGACTAAATCCCTCAACATGGCTAGCCTCTTTCTC
58               CACCAATTGTCACAGGGCTAATTCTGGACTAAATCCCTCAACATGGCTAGCCTCTTTCTC
68               CACCAATTGTCACAGGGCTAATTCTGGACTAAATCCCTCAACATGGCTAGCCTCTTTCTC
180              CACCAATTGTCACAGGGCTAATTCTGGACTAAATCCCTCAACATGGCTAGCCTCTTTCTC
81               CACCAATTGTCACAGGGCTAATTCTGGACTAAATCCCTCAACATGGCTAGCCTCTTTCTC
103              CACCAATTGTCACAGGGCTAATTCTGGACTAAATCCCTCAACATGGCTAGCCTCTTTCTC
113              CACCAATTGTCACAGGGCTAATTCTGGACTAAATCCCTCAACATGGCTAGCCTCTTTCTC
163              CACCAATTGTCACAGGGCTAATTCTGGACTAAATCCCTCAACATGGCTAGCCTCTTTCTC
40               CACCAATTGTCACAGGGCTAATTCTGGACTAAATCCCTCAACATGGCTAGCCTCTTTCTC
56               CACCAATTGTCACAGGGCTAATTCTGGACTAAATCCCTCAACATGGCTAGCCTCTTTCTC
218              CACCAATTGTCACAGGGCTAATTCTGGACTAAATCCCTCAACATGGCTAGCCTCTTTCTC
41               CACCAATTGTCACAGGGCTAATTCTGGACTAAATCCCTCAACATGGCTAGCCTCTTTCTC
42               CACCAATTGTCACAGGGCTAATTCTGGACTAAATCCCTCAACATGGCTAGCCTCTTTCTC
160              CACCAATTGTCACAGGGCTAATTCTGGACTAAATCCCTCAACATGGCTAGCCTCTTTCTC
5                CACCAATTGTCACAGGGCTAATTCTGGACTAAATCCCTCAACATGGCTAGCCTCTTTCTC
228              CACCAATTGTCACAGGGCTAATTCTGGACTAAATCCCTCAACATGGCTAGCCTCTTTCTC
17               CACCAATTGTCACAGGGCTAATTCTGGACTAAATCCCTCAACATGGCTAGCCTCTTTCTC
37               CACCAATTGTCACAGGGCTAATTCTGGACTAAATCCCTCAACATGGCTAGCCTCTTTCTC
91               CACCAATTGTCACAGGGCTAATTCTGGACTAAATCCCTCAACATGGCTAGCCTCTTTCTC
140              CACCAATTGTCACAGGGCTAATTCTGGACTAAATCCCTCAACATGGCTAGCCTCTTTCTC
167              CACCAATTGTCACAGGGCTAATTCTGGACTAAATCCCTCAACATGGCTAGCCTCTTTCTC
                 ************* *** ** **  ************************   ********

172              TTTTAATGGGGGTGGGATAAACTGCAACCAATATAAGAATGCTCATCAAAGAACTGCATG
229              TTTTAATGGGGGTGGGATAAACTGCAACCAATATAAGAATGCTCATCAAAGAACTGCATG
```

208	TTTGAATGGGGGTGGGATAAACTGCAACCAATATAAGAATGCTCATCAAAGAACTGCATG
83	TTTTAATGAGTGTGGTACAAACTGCAACCAATATAAGAATGCTCATCAAAGAACTGCATG
3	TTTTAATGAGTGTGGTACAAACTGCAACCAATATAAGAATGCTCATCAAAGAACTGCATG
120	TATGAATGAGTATGGGATAAACTGCAACCAATATAAGAATGCTCATCAAAGAACTGCATG
99	TATGAATGAGTATGGTATAAACTGCAACCAATATAAGAATGCTCATCAAAGAACTGCATG
126	TATGAATGAGTATGGTATAAACTGCAACCTATATAAGAATGCTCATCAAAGAACTGCGTG
173	TATGAATGAGTATGGTATAAACTGCAACCTATATAAGAATGCTCATCAAAGAACTGCGTG
204	TATGAATGAGTATGGTATAAACTGCAACCAATATAAGAATGCTCATCAAAGAACTGCATG
239	TATGAATGAGTATGGTATAAACTGCAACCAATATAAGAATGCTCATCAAAGAACTGCATG
117	TATGAATGAGTATGGTATAAACTGCAACCAATATAAGAATGCTCATCAAAGAACTGCATG
123	TATGAATGAGTATGGTATAAACTGCAACCAATATAAGAATGCTCATCAAAGAACTGCATG
210	TATGAATGAGTATGGGATAAACTGCAACCAATATAAGAATGCTCATCAAAGAACTGCATG
86	TATGAATGAGTATGGGATAAACTGCAACCAATATAAGAATGCTCATCAAAGAACTGCATG
100	TATGAATGAGTATGGGATAAACTGCAACCAATATAAGAATGCTCATCAAAGAACTGCATG
114	TATGAATGAGTATGGGATAAACTGCAACCAATATAAGAATGCTCATCAAAGAACTGCATG
170	TATGAATGAGTATGGGATAAACTGCAACCAATATAAGAATGCTCATCAAAGAACTGCATG
108	TATGAATGAGTATGGTATAAACTGCAACCTATATAAGAATGCTCATCAAAGAACTGCATG
174	TATGAATGAGTATGGTATAAACTGCAACCTATATAAGAATGCTCATCAAAGAACTGCATG
158	TATGAATGAGTATGGTATAAACTGCAACCAATATAAGAATGCTCATCAAAGAACTGCATG
154	TATGAATGAGTATGGTATAAACTGCAACCAATATAAGAATGCTCATCAAAGAACTGCATG
30	TATGAATGAGTAGGGGATAAACTGCAACCTATATAAGAATGCTCATCAAAGAACTGCGTG
39	TATGAATGAGTAGGGGATAAACTGCAACCTATATAAGAATGCTCATCAAAGAACTGCGTG
237	TATGAATGAGTATGGTATAAACTGCAACCAATATAAGAATGCTCATCAAAGAACTGCATG
65	TATGAATGAGTATGGTATAAACTGCAACCAATATAAGAATGCTCATCAAAGAACTGCATG
44	TATGAATGAGTATGGGATAAACTGCAACCAATATAAGAATGCTCATCAAAGAACTGCATG
168	TATGAATGAGTATGGTATAAACTGCAACCAATATAAGAATGCTCATCAAAGAACTGCATG
95	TATGAATGAGTATGGTATAAACTGCAACCAATATAAGAATGCTCATCAAAGAACTGCATG
22	TATGAATGAGTATGGGATAAACTGCAACCAATATAAGAATGCTCATCAAAGAACTGCATG
79	TATGAATGAGTATGGTATAAACTGCAACCAATATAAGAATGCTCATCAAAGAACTGCATG
211	TATGAATGAGTATGGGATAAACTGCAACCAATATAAGAATGCTCATCAAAGAACTGCATG
88	TATGAATGAGTATGGTATAAACTGCAACCAATATAAGAATGCTCATCAAAGAACTGCATG
184	TATGAATGAGTATGGTATAAACTGCAACCAATATAAGAATGCTCATCAAAGAACTGCATG
78	TATGAATGAGTATGGTATAAACTGCAACCAATATAAGAATGCTCATCAAAGAACTGCATG
71	TATGAATGAGTATGGTATAAACTGCAACCAATATAAGAATGCTCATCAAAGAACTGCATG
196	TATGAATGAGTATGGTATAAACTGCAACCAATATAAGAATGCTCATCAAAGAACTGCATG
43	TATGAATGAGTATGGTATAAACTGCAACCAATATAAGAATGCTCATCAAAGAACTGCATG
151	TATGAATGAGTATGGTATAAACTGCAACCAATATAAGAATGCTCATCAAAGAACTGCATG
27	TATGAATGAGTATGGTATAAACTGCAACCAATATAAGAATGCTCATCAAAGAACTGCATG
197	TATGAATGAGTATGGTATAAACTGCAACCAATATAAGAATGCTCATCAAAGAACTGCATG
109	TATGAATGAGTATGGTATAAACTGCAACCAATATAAGAATGCTCATCAAAGAACTGCGTG
169	TATGAATGAGTATGGTATAAACTGCAACCAATATAAGAATGCTCATCAAAGAACTGCATG
94	TATGAATGAGTATGGTATAAACTGCAACCAATATAAGAATGCTCATCAAAGAACTGCATG
232	TATGAATGAGTATGGTATAAACTGCAACCAATATAAGAATGCTCATCAAAGAACTGCATG
222	TATGAATGAGTATGGTATAAACTGCAACCAATATAAGAATGCTCATCAAAGAACTGCATG
24	TATGAATGAGTATGGTATAAACTGCAACCAATATAAGAATGCTCATCAAAGAACTGCATG
15	TATGAATGAGTATGGTATAAACTGCAACCAATATAAGAATGCTCATCAAAGAACTGCATG
13	TATGAATGAGTATGGTATAAACTGCAACCAATATAAGAATGCTCATCAAAGAACTGCATG
14	TATGAATGAGTATGGTATAAACTGCAACCTATATAAGAATGCTCATCAAAGAACTGCATG
35	TATGAATGAGTATGGTATAAACTGCAACCTATATAAGAATGCTCATCAAAGAACTGCATG
111	TATGAATGAGTATGGTATAAACTGCAACCTATATAAGAATGCTCATCAAAGAACTGCATG
157	TATGAATGAGTATGGTATAAACTGCAACCAATATAAGAATGCTCATCAAAGAACTGCATG
31	TATGAATGAGTATGGTATAAACTGCAACCAATATAAGAATGCTCATCAAAGAACTGCATG
47	TATGAATGAGTATGGTATAAACTGCAACCAATATAAGAATGCTCATCAAAGAACTGCATG
33	TATGAATGAGTATGGTATAAACTGCAACCAATATAAGAATGCTCATCAAAGAACTGCATG
51	TATGAATGAGTATGGTATAAACTGCAACCAATATAAGAATGCTCATCAAAGAACTGCATG
85	TATGAATGAGTATGGTATAAACTGCAACCAATATAAGAATGCTCATCAAAGAACTGCATG
54	TATGAATGAGTATGGTATAAACTGCAACCAATATAAGAATGCTCATCAAAGAACTGCATG

96	TATGAATGAGTATGGTATAAACTGCAACCAATATAAGAATGCTCATCAAAGAACTGCATG
61	TATGAATGAGTATGGTATAAACTGCAACCAATATAAGAATGCTCATCAAAGAACTGCATG
80	TATGAATGAGTATGGTATAAACTGCAACCAATATAAGAATGCTCATCAAAGAACTGCATG
82	TATGAATGAGTATGGTATAAACTGCAACCAATATAAGAATGCTCATCAAAGAACTGCATG
129	TATGAATGAGTATGGTATAAACTGCAACCAATATAAGAATGCTCATCAAAGAACTGCATG
46	TATGAATGAGTATGGTATAAACTGCAACCAATATAAGAATGCTCATCAAAGAACTGCATG
74	TATGAATGAGTATGGTATAAACTGCAACCAATATAAGAATGCTCATCAAAGAACTGCATG
101	TATGAATGAGTATGGGATAAACTGCAACCAATATAAGAATGCTCATCAAAGAACTGCATG
66	TATGAATGAGTATGGTATAAACTGCAACCAATATAAGAATGCTCATCAAAGAACTGCGTG
104	TATGAATGAGTATGGTATAAACTGCAACCAATATAAGAATGCTCATCAAAGAACTGCGTG
118	TATGAATGAGTATGGTATAAACTGCAACCAATATAAGAATGCTCATCAAAGAACTGCGTG
21	TATGAATGAGTATGGTATAAACTGCAACCAATATAAGAATGCTCATCAAAGAACTGCATG
77	TATGAATGAGTATGGTATAAACTGCAACCAATATAAGAATGCTCATCAAAGAACTGCATG
124	TATGAATGAGTATGGTATAAACTGCAACCAATATAAGAATGCTCATCAAAGAACTGCATG
20	TATGAATGAGTATGGTATAAACTGCAACCAATATAAGAATGCTCATCAAAGAACTGCATG
110	TATGAATGAGTATGGTATAAACTGCAACCAATATAAGAATGCTCATCAAAGAACTGCATG
70	TATGAATGAGTATGGTACAAACTGCAACCAATATAAGAATGCTCATCAAAGAACTGCATG
8	TATGAATGAGTATGGTACAAACTGCAACCAATATAAGAATGCTCATCAAAGAACTGCATG
55	TATGAATGAGTATGGTACAAACTGCAACCAATATAAGAATGCTCATCAAAGAACTGCATG
64	TATGAATGAGTATGGTACAAACTGCAACCAATATAAGAATGCTCATCAAAGAACTGCATG
93	TATGAATGAGTATGGTACAAACTGCAACCAATATAAGAATGCTCATCAAAGAACTGCATG
202	TATGAATGAGTATGGTACAAACTGCAACCAATATAAGAATGCTCATCAAAGAACTGCATG
10	TATGAATGAGTATGGTATAAACTGCAACCAATATAAGAATGCTCATCAAAGAACTGCATG
16	TATGAATGAGTATGGTATAAACTGCAACCAATATAAGAATGCTCATCAAAGAACTGCATG
76	TATGAATGAGTATGGTATAAACTGCAACCAATATAAGAATGCTCATCAAAGAACTGCATG
116	TATGAATGAGTATGGTATAAACTGCAACCAATATAAGAATGCTCATCAAAGAACTGCATG
220	TATGAATGAGTATGGTATAAACTGCAACCAATATAAGAATGCTCATCAAAGAACTGCATG
234	TATGAATGAGTATGGTATAAACTGCAACCAATATAAGAATGCTCATCAAAGAACTGCATG
2	TATGAATGAGTATGGTATAAACTGCAACCAATATAAGAATGCTCATCAAAGAACTGCATG
38	TATGAATGAGTATGGTATAAACTGCAACCAATATAAGAATGCTCATCAAAGAACTGCATG
73	TATGAATGAGTATGGTATAAACTGCAACCAATATAAGAATGCTCATCAAAGAACTGCATG
87	TATGAATGAGTATGGTATAAACTGCAACCAATATAAGAATGCTCATCAAAGAACTGCATG
92	TATGAATGAGTATGGTATAAACTGCAACCAATATAAGAATGCTCATCAAAGAACTGCATG
98	TATGAATGAGTATGGTATAAACTGCAACCAATATAAGAATGCTCATCAAAGAACTGCATG
137	TATGAATGAGTATGGTATAAACTGCAACCAATATAAGAATGCTCATCAAAGAACTGCATG
19	TATGAATGAGTATGGTATAAACTGCAACCAATATAAGAATGCTCATCAAAGAACTGCATG
171	TATGAATGAGTATGGTATAAACTGCAACCAATATAAGAATGCTCATCAAAGAACTGCATG
205	TATGAATGAGTAGGGTATAAACTGCAACCAATATAAGAATGCTCATCAAAGAACTGCATG
75	TATGAATGAGTAGGGTATAAACTGCAACCAATATAAGAATGCTCATCAAAGAACTGCATG
62	TATGAATGAGTATGGTATAAACTGCAACCAATATAAGAATGCTCATCAAAGAACTGCATG
4	TATGAATGAGTATGGTATAAACTGCAACCAATATAAGAATGCTCATCAAAGAACTGCATG
7	TATGAATGAGTATGGTATAAACTGCAACCAATATAAGAATGCTCATCAAAGAACTGCATG
25	TATGAATGAGTATGGTATAAACTGCAACCAATATAAGAATGCTCATCAAAGAACTGCATG
28	TATGAATGAGTATGGTATAAACTGCAACCAATATAAGAATGCTCATCAAAGAACTGCATG
36	TATGAATGAGTATGGTATAAACTGCAACCAATATAAGAATGCTCATCAAAGAACTGCATG
49	TATGAATGAGTATGGTATAAACTGCAACCAATATAAGAATGCTCATCAAAGAACTGCATG
145	TATGAATGAGTATGGTATAAACTGCAACCAATATAAGAATGCTCATCAAAGAACTGCATG
181	TATGAATGAGTATGGTATAAACTGCAACCAATATAAGAATGCTCATCAAAGAACTGCATG
188	TATGAATGAGTATGGTATAAACTGCAACCAATATAAGAATGCTCATCAAAGAACTGCATG
59	TATGAATGAGTATGGTATAAACTGCAACCAATATAAGAATGCTCATCAAAGAACTGCATG
121	TATGAATGAGTATGGTATAAACTGCAACCAATATAAGAATGCTCATCAAAGAACTGCATG
127	TATGAATGAGTATGGTATAAACTGCAACCAATATAAGAATGCTCATCAAAGAACTGCATG
135	TATGAATGAGTATGGTATAAACTGCAACCAATATAAGAATGCTCATCAAAGAACTGCATG
136	TATGAATGAGTATGGTATAAACTGCAACCAATATAAGAATGCTCATCAAAGAACTGCATG
29	TATGAATGAGTATGGTATAAACTGCAACCAATATAAGAATGCTCATCAAAGAACTGCATG
89	TATGAATGAGTATGGTATAAACTGCAACCAATATAAGAATGCTCATCAAAGAACTGCATG
216	TATGAATGAGTATGGTATAAACTGCAACCAATATAAGAATGCTCATCAAAGAACTGCATG

164 TATGAATGAGTATGGTATAAACTGCAACCAATATAAGAATGCTCATCAAAGAACTGCATG
63 TATGAATGAGTATGGTATAAACTGCAACCAATATAAGAATGCTCATCAAAGAACTGCATG
161 TATGAATGAGTATGGTATAAACTGCAACCAATATAAGAATGCTCATCAAAGAACTGCATG
183 TATGAATGAGTATGGTATAAACTGCAACCAATATAAGAATGCTCATCAAAGAACTGCATG
18 TATGAATGAGTATGGTATAAACTGCAACCAATATAAGAATGCTCATCAAAGAACTGCATG
90 TATGAATGAGTATGGTATAAACTGCAACCAATATAAGAATGCTCATCAAAGAACTGCATG
162 TATGAATGAGTATGGTATAAACTGCAACCAATATAAGAATGCTCATCAAAGAACTGCATG
177 TATGAATGAGTATGGTATAAACTGCAACCAATATAAGAATGCTCATCAAAGAACTGCATG
97 TATGAATGAGTATGGTATAAACTGCAACCAATATAAGAATGCTCATCAAAGAACTGCATG
231 TATGAATGAGTATGGTATAAACTGCAACCAATATAAGAATGCTCATCAAAGAACTGCATG
143 TATGAATGAGTATGGTATAAACTGCAACCAATATAAGAATGCTCATCAAAGAACTGCATG
11 TATGAATGAGTATGGTATAAACTGCAACCAATATAAGAATGCTCATCAAAGAACTGCATG
153 TATGAATGAGTATGGTATAAACTGCAACCAATATAAGAATGCTCATCAAAGAACTGCATG
138 TATGAATGAGTATGGTATAAACTGCAACCAATATAAGAATGCTCATCAAAGAACTGCATG
185 TATGAATGAGTATGGTATAAACTGCAACCAATATAAGAATGCTCATCAAAGAACTGCATG
194 TATGAATGAGTATGGTATAAACTGCAACCAATATAAGAATGCTCATCAAAGAACTGCATG
236 TATGAATGAGTATGGTATAAACTGCAACCAATATAAGAATGCTCATCAAAGAACTGCATG
32 TATGAATGAGTATGGTATAAACTGCAACCAATATAAGAATGCTCATCAAAGAACTGCATG
52 TATGAATGAGTATGGTATAAACTGCAACCAATATAAGAATGCTCATCAAAGAACTGCATG
60 TATGAATGAGTATGGTATAAACTGCAACCAATATAAGAATGCTCATCAAAGAACTGCATG
72 TATGAATGAGTATGGTATAAACTGCAACCAATATAAGAATGCTCATCAAAGAACTGCATG
112 TATGAATGAGTATGGTATAAACTGCAACCAATATAAGAATGCTCATCAAAGAACTGCATG
141 TATGAATGAGTATGGTATAAACTGCAACCAATATAAGAATGCTCATCAAAGAACTGCATG
155 TATGAATGAGTATGGTATAAACTGCAACCAATATAAGAATGCTCATCAAAGAACTGCATG
1 TATGAATGAGTATGGTATAAACTGCAACCAATATAAGAATGCTCATCAAAGAACTGCATG
6 TATGAATGAGTATGGTATAAACTGCAACCAATATAAGAATGCTCATCAAAGAACTGCATG
58 TATGAATGAGTATGGTATAAACTGCAACCAATATAAGAATGCTCATCAAAGAACTGCATG
68 TATGAATGAGTATGGTATAAACTGCAACCAATATAAGAATGCTCATCAAAGAACTGCATG
180 TATGAATGAGTATGGTATAAACTGCAACCAATATAAGAATGCTCATCAAAGAACTGCATG
81 TATGAATGAGTATGGTATAAACTGCAACCAATATAAGAATGCTCATCAAAGAACTGCATG
103 TATGAATGAGTATGGTATAAACTGCAACCAATATAAGAATGCTCATCAAAGAACTGCATG
113 TATGAATGAGTATGGTATAAACTGCAACCAATATAAGAATGCTCATCAAAGAACTGCATG
163 TATGAATGAGTATGGTATAAACTGCAACCAATATAAGAATGCTCATCAAAGAACTGCATG
40 TATGAATGAGTATGGTATAAACTGCAACCAATATAAGAATGCTCATCAAAGAACTGCATG
56 TATGAATGAGTATGGTATAAACTGCAACCAATATAAGAATGCTCATCAAAGAACTGCATG
218 TATGAATGAGTATGGTATAAACTGCAACCAATATAAGAATGCTCATCAAAGAACTGCATG
41 TATGAATGAGTATGGTATAAACTGCAACCAATATAAGAATGCTCATCAAAGAACTGCATG
42 TATGAATGAGTATGGTATAAACTGCAACCAATATAAGAATGCTCATCAAAGAACTGCATG
160 TATGAATGAGTATGGTATAAACTGCAACCAATATAAGAATGCTCATCAAAGAACTGCATG
5 TATGAATGAGTATGGTATAAACTGCAACCAATATAAGAATGCTCATCAAAGAACTGCATG
228 TATGAATGAGTATGGTATAAACTGCAACCAATATAAGAATGCTCATCAAAGAACTGCATG
17 TATGAATGAGTATGGTATAAACTGCAACCAATATAAGAATGCTCATCAAAGAACTGCATG
37 TATGAATGAGTATGGTATAAACTGCAACCAATATAAGAATGCTCATCAAAGAACTGCATG
91 TATGAATGAGTATGGTATAAACTGCAACCAATATAAGAATGCTCATCAAAGAACTGCATG
140 TATGAATGAGTATGGTATAAACTGCAACCAATATAAGAATGCTCATCAAAGAACTGCGTG
167 TATGAATGAGTATGGTATAAACTGCAACCAATATAAGAATGCTCATCAAAGAACTGCGTG
* * **** *   ** * *********** *************************** **

172 TGCGATCTAGATAAACAGAGCCATTATGAAGTAAAAAA
229 TGCGATCTAGATAAACAGAGCCATTATGAAGTAAAAAA
208 TGCGATCTAGATAAACAGAGCCATTATGAAGTAAAAAA
83 TGCAATCTAGATAAACAGAGCCATTATGAAGTAAAAAA
3 TGCAATCTAGATAAACAGAGCCATTATGAAGTAAAAAA
120 TGCAATCTAGATAAACAGAGCCATTATGAAGTAAAAAA
99 TGCAATCTAGATAAACAGAGCCATTATGAAGTAAAAAA
126 TGCAATCTAGATAAACAGAGCCATTATGAAGTAAAAAA
173 TGCAATCTAGATAAACAGAGCCATTATGAAGTAAAAAA

204	TGCAATCTAGATAAACAGAGCCATTATGAAGTAAAAAA
239	TGCAATCTAGATAAACAGAGCCATTATGAAGTAAAAAA
117	TGCAATCTAGATAAACAGAGCCATTATGAAGTAAAAAA
123	TGCAATCTAGATAAACAGAGCCATTATGAAGTAAAAAA
210	TGCGATCTAGATAAACAGAGCCATTATGAAGTAAAAAA
86	TGCGATCTAGATAAACAGAGCCATTATGAAGTAAAAAA
100	TGCGATCTAGATAAACAGAGCCATTATGAAGTAAAAAA
114	TGCGATCTAGATAAACAGAGCCATTATGAAGTAAAAAA
170	TGCGATCTAGATAAACAGAGCCATTATGAAGTAAAAAA
108	TGCAATCTAGATAAACAGAGCCATTATGAAGTAAAAAA
174	TGCAATCTAGATAAACAGAGCCATTATGAAGTAAAAAA
158	TGCGATCTAGATAAACAGAGCCATTATGAAGTAAAAAA
154	TGCAATCTAGATAAACAGAGCCATTATGAAGTAAAAAA
30	TGCAATCTAGATAAACAGAGCCATTATGAAGTAAAAAA
39	TGCAATCTAGATAAACAGAGCCATTATGAAGTAAAAAA
237	TGCAATCTAGATAAACAGAGCCATTATGAAGTAAAAAA
65	TGCAATCTAGATAAACAGAGCCATTATGAAGTAAAAAA
44	TGCAATCTAGATAAACAGAGCCATTATGAAGTAAAAAA
168	TGCAATCTAGATAAACAGAGCCATTATGAAGTAAAAAA
95	TGCAATCTAGATAAACAGAGCCATTATGAAGTAAAAAA
22	TGCGATCTAGATAAACAGAGCCATTATGAAGTAAAAAA
79	TGCAATCTAGATAAACAGAGCCATTATGAAGTAAAAAA
211	TGCAATCTAGATAAACAGAGCCATTATGAAGTAAAAAA
88	TGCGATCTAGATAAACAGAGCCATTATGAAGTAAAAAA
184	TGCGATCTAGATAAACAGAGCCATTATGAAGTAAAAAA
78	TGCAATCTAGATAAACAGAGCCATTATGAAGTAAAAAA
71	TGCAATCTAGATAAACAGAGCCATTATGAAGTAAAAAA
196	TGCAATCTAGATAAACAGAGCCATTATGAAGTAAAAAA
43	TGCAATCTAGATAAACAGAGCCATTATGAAGTAAAAAA
151	TGCAATCTAGATAAACAGAGCCATTATGAAGTAAAAAA
27	TGCAATCTAGATAAACAGAGCCATTATGAAGTAAAAAA
197	TGCAATCTAGATAAACAGAGCCATTATGAAGTAAAAAA
109	TGCAATCTAGATAAACAGAGCCATTATGAAGTAAAAAA
169	TGCAATCTAGATAAACAGAGCCATTATGAAGTAAAAAA
94	TGCGATCTAGATAAACAGAGCCATTATGAAGTAAAAAA
232	TGCGATCTAGATAAACAGAGCCATTATGAAGTAAAAAA
222	TGCAATCTAGATAAACAGAGCCATTATGAAGTAAAAAA
24	TGCAATCTAGATAAACAGAGCCATTATGAAGTAAAAAA
15	TGCAATCTAGATAAACAGAGCCATTATGAAGTAAAAAA
13	TGCAATCTAGATAAACAGAGCCATTATGAAGTAAAAAA
14	TGCAATCTAGATAAACAGAGCCATTATGAAGTAAAAAA
35	TGCAATCTAGATAAACAGAGCCATTATGAAGTAAAAAA
111	TGCAATCTAGATAAACAGAGCCATTATGAAGTAAAAAA
157	TGCAATCTAGATAAACAGAGCCATTATGAAGTAAAAAA
31	TGCAATCTAGATAAACAGAGCCATTATGAAGTAAAAAA
47	TGCAATCTAGATAAACAGAGCCATTATGAAGTAAAAAA
33	TGCAATCTAGATAAACAGAGCCATTATGAAGTAAAAAA
51	TGCAATCTAGATAAACAGAGCCATTATGAAGTAAAAAA
85	TGCAATCTAGATAAACAGAGCCATTATGAAGTAAAAAA
54	TGCAATCTAGATAAACAGAGCCATTATGAAGTAAAAAA
96	TGCAATCTAGATAAACAGAGCCATTATGAAGTAAAAAA
61	TGCAATCTAGATAAACAGAGCCATTATGAAGTAAAAAA
80	TGCAATCTAGATAAACAGAGCCATTATGAAGTAAAAAA
82	TGCAATCTAGATAAACAGAGCCATTATGAAGTAAAAAA
129	TGCAATCTAGATAAACAGAGCCATTATGAAGTAAAAAA
46	TGCAATCTAGATAAACAGAGCCATTATGAAGTAAAAAA
74	TGCAATCTAGATAAACAGAGCCATTATGAAGTAAAAAA

101	TGCAATCTAGATAAACAGAGCCATTATGAAGTAAAAAA
66	TGCAATCTAGATAAACAGAGCCATTATGAAGTAAAAAA
104	TGCAATCTAGATAAACAGAGCCATTATGAAGTAAAAAA
118	TGCAATCTAGATAAACAGAGCCATTATGAAGTAAAAAA
21	TGCAATCTAGATAAACAGAGCCATTATGAAGTAAAAAA
77	TGCAATCTAGATAAACAGAGCCATTATGAAGTAAAAAA
124	TGCAATCTAGATAAACAGAGCCATTATGAAGTAAAAAA
20	TGCAATCTAGATAAACAGAGCCATTATGAAGTAAAAAA
110	TGCAATCTAGATAAACAGAGCCATTATGAAGTAAAAAA
70	TGCAATCTAGATAAACAGAGCCATTATGAAGTAAAAAA
8	TGCAATCTAGATAAACAGAGCCATTATGAAGTAAAAAA
55	TGCAATCTAGATAAACAGAGCCATTATGAAGTAAAAAA
64	TGCAATCTAGATAAACAGAGCCATTATGAAGTAAAAAA
93	TGCAATCTAGATAAACAGAGCCATTATGAAGTAAAAAA
202	TGCAATCTAGATAAACAGAGCCATTATGAAGTAAAAAA
10	TGCAATCTAGATAAACAGAGCCATTATGAAGTAAAAAA
16	TGCAATCTAGATAAACAGAGCCATTATGAAGTAAAAAA
76	TGCAATCTAGATAAACAGAGCCATTATGAAGTAAAAAA
116	TGCAATCTAGATAAACAGAGCCATTATGAAGTAAAAAA
220	TGCAATCTAGATAAACAGAGCCATTATGAAGTAAAAAA
234	TGCAATCTAGATAAACAGAGCCATTATGAAGTAAAAAA
2	TGCAATCTAGATAAACAGAGCCATTATGAAGTAAAAAA
38	TGCAATCTAGATAAACAGAGCCATTATGAAGTAAAAAA
73	TGCAATCTAGATAAACAGAGCCATTATGAAGTAAAAAA
87	TGCAATCTAGATAAACAGAGCCATTATGAAGTAAAAAA
92	TGCAATCTAGATAAACAGAGCCATTATGAAGTAAAAAA
98	TGCAATCTAGATAAACAGAGCCATTATGAAGTAAAAAA
137	TGCAATCTAGATAAACAGAGCCATTATGAAGTAAAAAA
19	TGCAATCTAGATAAACAGAGCCATTATGAAGTAAAAAA
171	TGCAATCTAGATAAACAGAGCCATTATGAAGTAAAAAA
205	TGCAATCTAGATAAACAGAGCCATTATGAAGTAAAAAA
75	TGCAATCTAGATAAACAGAGCCATTATGAAGTAAAAAA
62	TGCAATCTAGATAAACAGAGCCATTATGAAGTAAAAAA
4	TGCAATCTAGATAAACAGAGCCATTATGAAGTAAAAAA
7	TGCAATCTAGATAAACAGAGCCATTATGAAGTAAAAAA
25	TGCAATCTAGATAAACAGAGCCATTATGAAGTAAAAAA
28	TGCAATCTAGATAAACAGAGCCATTATGAAGTAAAAAA
36	TGCAATCTAGATAAACAGAGCCATTATGAAGTAAAAAA
49	TGCAATCTAGATAAACAGAGCCATTATGAAGTAAAAAA
145	TGCAATCTAGATAAACAGAGCCATTATGAAGTAAAAAA
181	TGCAATCTAGATAAACAGAGCCATTATGAAGTAAAAAA
188	TGCAATCTAGATAAACAGAGCCATTATGAAGTAAAAAA
59	TGCAATCTAGATAAACAGAGCCATTATGAAGTAAAAAA
121	TGCAATCTAGATAAACAGAGCCATTATGAAGTAAAAAA
127	TGCAATCTAGATAAACAGAGCCATTATGAAGTAAAAAA
135	TGCAATCTAGATAAACAGAGCCATTATGAAGTAAAAAA
136	TGCAATCTAGATAAACAGAGCCATTATGAAGTAAAAAA
29	TGCAATCTAGATAAACAGAGCCATTATGAAGTAAAAAA
89	TGCAATCTAGATAAACAGAGCCATTATGAAGTAAAAAA
216	TGCAATCTAGATAAACAGAGCCATTATGAAGTAAAAAA
164	TGCAATCTAGATAAACAGAGCCATTATGAAGTAAAAAA
63	TGCAATCTAGATAAACAGAGCCATTATGAAGTAAAAAA
161	TGCAATCTAGATAAACAGAGCCATTATGAAGTAAAAAA
183	TGCAATCTAGATAAACAGAGCCATTATGAAGTAAAAAA
18	TGCAATCTAGATAAACAGAGCCATTATGAAGTAAAAAA
90	TGCAATCTAGATAAACAGAGCCATTATGAAGTAAAAAA
162	TGCAATCTAGATAAACAGAGCCATTATGAAGTAAAAAA

```
177              TGCAATCTAGATAAACAGAGCCATTATGAAGTAAAAAA
97               TGCAATCTAGATAAACAGAGCCATTATGAAGTAAAAAA
231              TGCAATCTAGATAAACAGAGCCATTATGAAGTAAAAAA
143              TGCAATCTAGATAAACAGAGCCATTATGAAGTAAAAAA
11               TGCAATCTAGATAAACAGAGCCATTATGAAGTAAAAAA
153              TGCAATCTAGATAAACAGAGCCATTATGAAGTAAAAAA
138              TGCAATCTAGATAAACAGAGCCATTATGAAGTAAAAAA
185              TGCAATCTAGATAAACAGAGCCATTATGAAGTAAAAAA
194              TGCAATCTAGATAAACAGAGCCATTATGAAGTAAAAAA
236              TGCAATCTAGATAAACAGAGCCATTATGAAGTAAAAAA
32               TGCAATCTAGATAAACAGAGCCATTATGAAGTAAAAAA
52               TGCAATCTAGATAAACAGAGCCATTATGAAGTAAAAAA
60               TGCAATCTAGATAAACAGAGCCATTATGAAGTAAAAAA
72               TGCAATCTAGATAAACAGAGCCATTATGAAGTAAAAAA
112              TGCAATCTAGATAAACAGAGCCATTATGAAGTAAAAAA
141              TGCAATCTAGATAAACAGAGCCATTATGAAGTAAAAAA
155              TGCAATCTAGATAAACAGAGCCATTATGAAGTAAAAAA
1                TGCAATCTAGATAAACAGAGCCATTATGAAGTAAAAAA
6                TGCAATCTAGATAAACAGAGCCATTATGAAGTAAAAAA
58               TGCAATCTAGATAAACAGAGCCATTATGAAGTAAAAAA
68               TGCAATCTAGATAAACAGAGCCATTATGAAGTAAAAAA
180              TGCAATCTAGATAAACAGAGCCATTATGAAGTAAAAAA
81               TGCAATCTAGATAAACAGAGCCATTATGAAGTAAAAAA
103              TGCAATCTAGATAAACAGAGCCATTATGAAGTAAAAAA
113              TGCAATCTAGATAAACAGAGCCATTATGAAGTAAAAAA
163              TGCAATCTAGATAAACAGAGCCATTATGAAGTAAAAAA
40               TGCAATCTAGATAAACAGAGCCATTATGAAGTAAAAAA
56               TGCAATCTAGATAAACAGAGCCATTATGAAGTAAAAAA
218              TGCAATCTAGATAAACAGAGCCATTATGAAGTAAAAAA
41               TGCAATCTAGATAAACAGAGCCATTATGAAGTAAAAAA
42               TGCAATCTAGATAAACAGAGCCATTATGAAGTAAAAAA
160              TGCAATCTAGATAAACAGAGCCATTATGAAGTAAAAAA
5                TGCAATCTAGATAAACAGAGCCATTATGAAGTAAAAAA
228              TGCAATCTAGATAAACAGAGCCATTATGAAGTAAAAAA
17               TGCAATCTAGATAAACAGAGCCATTATGAAGTAAAAAA
37               TGCAATCTAGATAAACAGAGCCATTATGAAGTAAAAAA
91               TGCAATCTAGATAAACAGAGCCATTATGAAGTAAAAAA
140              TGCAATCTAGATAAACAGAGCCATTATGAAGTAAAAAA
167              TGCGATCTAGATAAACAGAGCCATTATGAAGTAAAAAA
                 *** **********************************
```

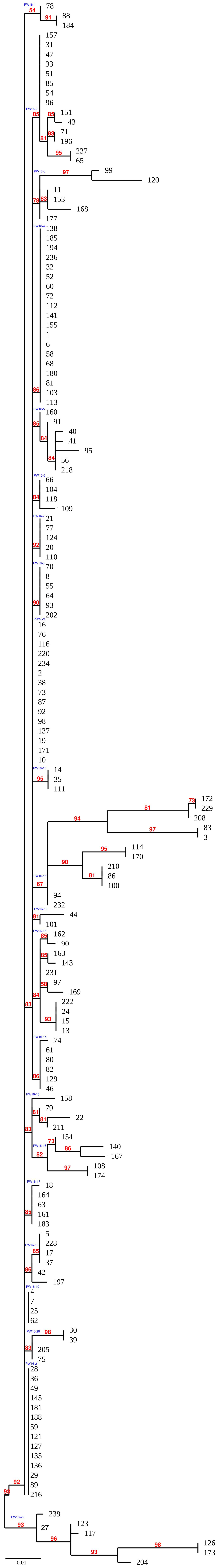

Figure S9. Neighbour-joining tree constructed with PW16 locus sequences from 162 *P. quinquefolius*samples from a commercial ginseng garden near Simcoe, Ontario. Sample number is shown in black, and cluster designation (PW16-1 to PW16-22) is shown in blue. Bootstrap support values from 1000 replicates are shown in red at the nodes. Scale bar is at bottom.

Table S1. Root parameters separated by root grade.

Root grade	Root fresh wt (g)	Root dry wt (g)	Water content (%)
Pencil	13.50 B[a]	3.85 B	28.17 A
Chunk	18.31 A	5.27 A	29.58 A
Fork	18.78 A	5.49 A	26.66 A
Spider	21.36 A	5.98 A	26.87 A

[a] Values within a column followed by a letter in common are not significantly different at p = 0.05 according to Fisher's protected Least Significant Difference test (LSD).

Table S2. Leaf parameters separated by root grade.

Root grade	Leaves per plant	Leaflets per plant	Leaf area (cm)	Leaf fresh wt (g)	Leaf dry wt (g)	Water content (%)
Pencil	3.04 A[a]	13.84 B	23.15 B	4.97 B	1.34 B	28.65 A
Chunk	3.00 A	13.56 A	28.81 A	6.47 A	1.60 A	25.80 B
Fork	3.09 A	13.50 A	29.80 A	6.35 A	1.58 A	25.99 B
Spider	3.03 A	13.39 A	29.70 A	6.52 A	1.61 A	26.45 AB

[a] Values within a column followed by a letter in common are not significantly different at p = 0.05 according to Fisher's protected Least Significant Difference test (LSD).

Table S3. Petiole and stem parameters separated by root grade.

Root grade	Petiole fresh wt (g)	Petiole dry wt (g)	Water content (%)	Stem length (cm)	Stem fresh wt (g)	Stem dry wt (g)	Water content (%)
Pencil	1.45 B[a]	0.19 B	13.14 A	29.23 B	4.16 B	0.58 B	13.50 A
Chunk	2.00 A	0.26 A	12.98 A	32.13 A	4.96 AB	0.71 AB	14.30 A
Fork	1.98 A	0.26 A	12.61 A	31.11 AB	5.53 A	0.78 A	13.87 A
Spider	1.94 A	0.28 A	14.01 A	30.10 AB	5.46 A	0.82 A	14.77 A

[a] Values within a column followed by a letter in common are not significantly different at p = 0.05 according to Fisher's protected Least Significant Difference test (LSD).

Table S4. Seed parameters separated b root grade.

Root grade	Seed petiole length		Seed petioles fresh wt (g)		Seed petioles dry wt (g)		Water content (%)		Seed petiolules per plant		Total seeds per plant		Seed fresh wt (g)		Seed dry wt (g)		Water content (%)	
Pencil	09.82	A[a]	0.42	B	0.10	B	23.64	A	42.59	A	0.52	A	1.98	A	0.43	A	19.43	A
Chunk	10.89	A	0.52	AB	0.14	A	27.58	A	39.94	A	0.66	A	2.06	A	0.44	A	21.33	A
Fork	11.33	A	0.50	AB	0.14	A	27.52	A	45.54	A	0.63	A	1.97	A	0.38	A	18.71	A
Spider	11.04	A	0.56	A	0.14	A	24.15	A	44.29	A	0.70	A	1.74	A	0.38	A	20.70	A

a Values within a column followed by a letter in common are not significantly different at p = 0.05 according to Fisher's protected Least Significant Difference test (LSD).

Table S5. Root, leaves, and leaf petiole growth parameters of *P. quinquefolius*. At 3-year old, root fresh weight, root dry weight, leaves per plant, and leaf petiole dry weight for different groups were recorded.

Group no.	Root fresh weight (g)	Root dry weight (g)	Leaves per plant	Leaf petiole dry weight (g)
1	18.73 A	5.38 A	2.67 A	0.26 A
2	19.27 A	5.44 A	2.79 A	0.27 A
3	18.33 A	5.52 A	2.67 A	0.21 A
4	17.08 A	4.73 A	3.32 A	0.24 A
5	20.32 A	5.88 A	2.86 A	0.30 A
6	18.01 A	4.94 A	3.00 A	0.23 A
7	21.03 A	6.44 A	3.00 A	0.30 A
8	12.50 A	3.20 A	3.33 A	0.21 A
9	14.06 A	3.76 A	3.00 A	0.21 A
10	13.77 A	3.79 A	2.67 A	0.17 A
11	22.14 A	6.60 A	3.42 A	0.29 A
12	17.11 A	5.26 A	3.00 A	0.25 A
13	14.31 A	4.20 A	3.09 A	0.19 A
14	13.24 A	3.40 A	3.00 A	0.18 A
15	12.50 A	3.10 A	3.25 A	0.21 A
16	15.27 A	4.44 A	3.00 A	0.18 A
17	17.61 A	5.44 A	3.20 A	0.22 A
18	15.89 A	4.61 A	3.00 A	0.19 A
19	15.18 A	4.59 A	3.00 A	0.21 A
20	14.98 A	4.14 A	3.00 A	0.20 A
21	17.28 A	4.87 A	2.93 A	0.23 A
22	12.80 A	3.72 A	3.00 A	0.18 A
